# Dynamics of bacterial recombination in the human gut microbiome

**DOI:** 10.1371/journal.pbio.3002472

**Published:** 2024-02-08

**Authors:** Zhiru Liu, Benjamin H. Good

**Affiliations:** 1 Department of Applied Physics, Stanford University, Stanford, California, United States of America; 2 Department of Biology, Stanford University, Stanford, California, United States of America; 3 Chan Zuckerberg Biohub–San Francisco, San Francisco, California, United States of America; University of Copenhagen Faculty of Health and Medical Sciences: Kobenhavns Universitet Sundhedsvidenskabelige Fakultet, DENMARK

## Abstract

Horizontal gene transfer (HGT) is a ubiquitous force in microbial evolution. Previous work has shown that the human gut is a hotspot for gene transfer between species, but the more subtle exchange of variation within species—also known as recombination—remains poorly characterized in this ecosystem. Here, we show that the genetic structure of the human gut microbiome provides an opportunity to measure recent recombination events from sequenced fecal samples, enabling quantitative comparisons across diverse commensal species that inhabit a common environment. By analyzing recent recombination events in the core genomes of 29 human gut bacteria, we observed widespread heterogeneities in the rates and lengths of transferred fragments, which are difficult to explain by existing models of ecological isolation or homology-dependent recombination rates. We also show that natural selection helps facilitate the spread of genetic variants across strain backgrounds, both within individual hosts and across the broader population. These results shed light on the dynamics of in situ recombination, which can strongly constrain the adaptability of gut microbial communities.

## Abstract 2

Recombination is a ubiquitous force in bacterial evolution, but its dynamics are poorly characterized in many natural populations. This study presents a metagenomic approach for quantifying the landscape of recombina0on in ~30 common species of human gut bacteria, revealing widespread variation both within and across species.

## Introduction

The horizontal exchange of genetic material—also known as horizontal gene transfer (HGT)—is a pervasive force in microbial ecology and evolution [1]. HGT is particularly important within the human gut microbiota, where hundreds of species coexist with each other in close physical proximity [2–4]. HGT is often associated with the acquisition of new genes or pathways, which can confer resistance to antibiotics [3–8] or enable novel metabolic capabilities [3,9–14]. Genetic material can also be transferred between more closely related strains, where it can overwrite existing regions of the genome via homologous recombination [15,16]. This more subtle form of horizontal exchange acts to reshuffle genetic variants within species, similar to meiotic recombination in sexual organisms. Homologous recombination plays a crucial role in microbial evolution, from the emergence of new bacterial species [17–20] to the transition between clonal and quasi-sexual evolution [21–23]. Homologous recombination can also serve as a scaffold for the incorporation of novel genetic material, which can facilitate the spread of accessory genes across different strain backgrounds [24]. However, while numerous studies have established the pervasiveness of bacterial recombination [21,25–28], the evolutionary dynamics of this process are still poorly understood in natural populations like the gut microbiota.

Multiple methods have been developed for inferring in situ recombination from the fine-scale diversity of natural bacterial isolates [26,27,29–33]. The key challenge lies in disentangling the effects of recombination from the other evolutionary forces (e.g., mutation, selection, and genetic drift) that shape genetic diversity over the same timescales. Existing studies often address this problem using an inverse approach, by fitting the observed data to simple parametric models from microbial population genetics. Examples range from simple summary statistics like linkage disequilibrium [26,27,34,35] and related metrics [21,28,32,33,36–40] to complete probabilistic reconstructions of the genealogies of the sampled genomes [29–31]. Previous applications of these methods have provided extensive evidence for ongoing recombination within the core genomes of many bacterial species [25,41]—including many species of human gut bacteria [27].

However, many of these existing methods rely on simplified evolutionary scenarios, which ignore the effects of natural selection, and make additional restrictive assumptions about the demographic structure of the population. Recent work has shown that these simplified models often fail to capture key features of microbial genetic diversity [26–28,42], which can strongly bias estimates of the underlying recombination parameters. Our limited understanding of these effects makes it difficult to answer key questions about the role of recombination in natural populations like the gut microbiota: Is recombination fast enough to allow local adaptations to persist within a host, e.g., during fecal microbiota transplants [43] or sudden dietary shifts [44]? Does natural selection tend to promote or hinder the spread of genetic variants across different strain backgrounds? And can the rates and lengths of transferred fragments shed light on the underlying mechanisms of recombination in situ?

Here, we show that the genetic structure of the human gut microbiome provides a unique opportunity to address these questions. Using strain-resolved metagenomics, we show that the large sample sizes and host colonization structure of this ecosystem enable systematic comparisons of strains across a broad range of distance and timescales, from the scale of individual hosts to the diversity of the broader global population. We show that some of these strains are closely related enough that one can resolve homologous recombination events directly, without requiring restrictive modeling assumptions or explicit phylogenetic inference. We use these observations to develop a nonparametric approach for identifying large numbers of recent recombination events within 29 prevalent species of human gut bacteria. This comparative data set allows us to systematically explore the landscape of homologous recombination in this host-associated ecosystem.

Our results reveal extensive heterogeneity in rates and lengths of transferred fragments—both among different species and between different strains of the same species—which are difficult to explain by ecological isolation or reduced efficiencies of recombination. We also find that natural selection can play an important role in facilitating the spread of transferred fragments into different strain backgrounds. Our results suggest that in situ recombination events are shaped by a combination of evolutionary processes, which may strongly depend on the ecological context of their host community.

## Results

### Partially recombined genomes underlie the broad range of genetic diversity in many species of gut bacteria

**Fig 1:**
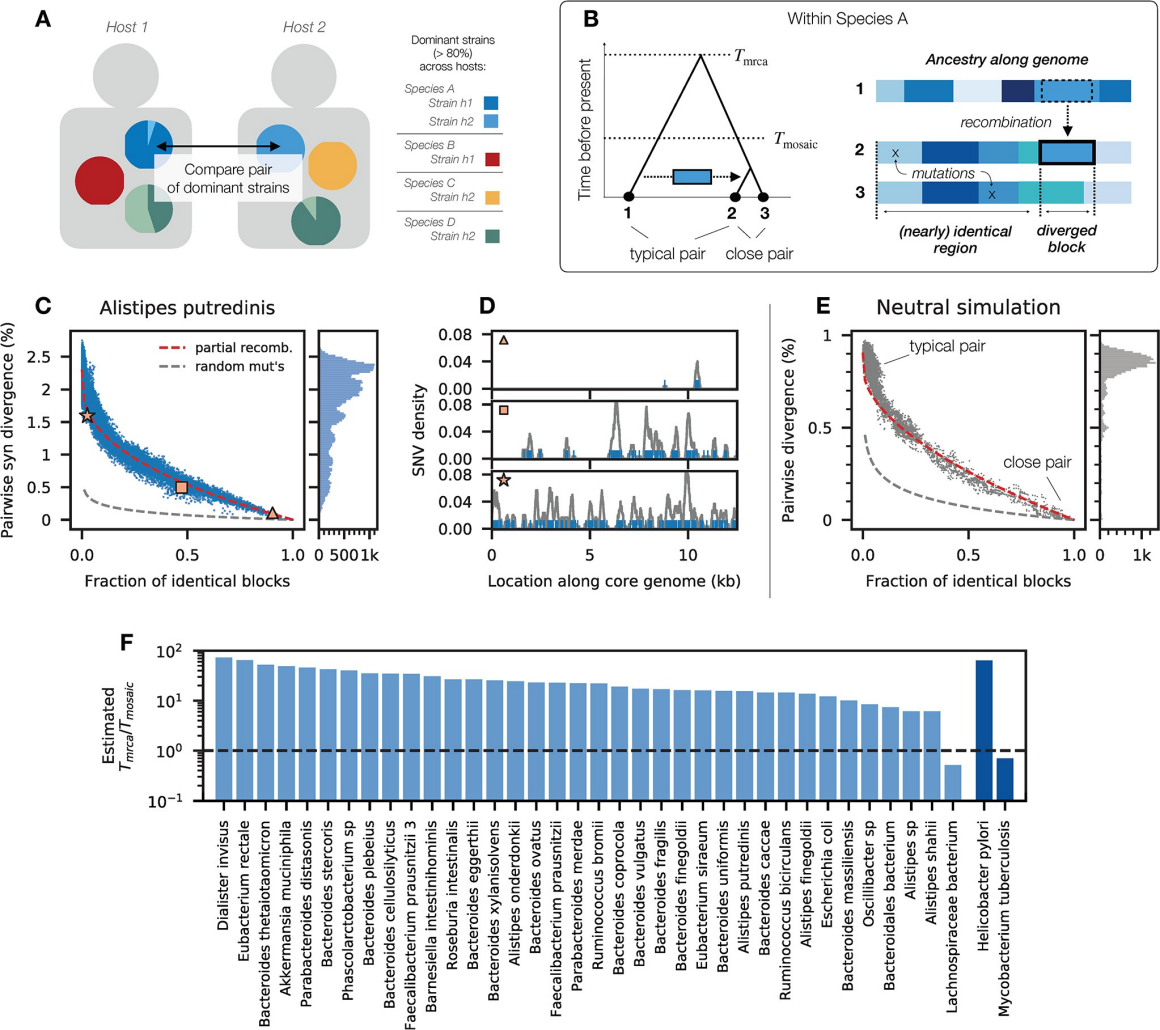
Partially recombined genomes underlie the broad range of genetic divergence in many species of gut bacteria. (A) Genetic differences between the core genomes of the dominant strain of a given species (>80% within-host frequency) are inferred from pairwise comparisons of metagenomes from unrelated hosts (Section 1 in S1 Text). (B) Timescales of recombination in a quasi-sexual bacterial population: most strains share a common ancestor ≫ *T*_mosaic_ generations ago, so their present-day genomes are completely overwritten by recombination; in large samples, some pairs of strains will share a common ancestor ≪ *T*_mosaic_ generations ago, and recombination events will be visible as blocks of local divergence against a shared clonal background. (C) Average synonymous divergence vs. fraction of identical blocks for pairs of *A*. *putredinis* strains from unrelated hosts (Section 2 in S1 Text). Points denote individual pairs, while the marginal distribution is shown on the right; red line shows the expectation from a simple model of accumulated transfers (Section 2 in S1 Text), while gray line shows the expectation when mutations are randomly distributed across the genome. (D) Spatial distribution of synonymous SNVs for 3 example pairs from panel C (symbols); only a portion of the core genome is shown. Points denote individual SNVs, while lines show the local divergence in sliding 300 bp windows. (E) Analogous version of C for neutral simulations (Section 5.3 in S1 Text, Fig B in S1 Text). (F) Inferred values of *T*_mrca_/*T*_mosaic_ for the partial recombination model in Section 2 in S1 Text; 2 additional species (*H*. *pylori* and *M*. *tuberculosis*) are shown on the right for comparison. The data underlying this figure can be found in https://doi.org/10.5281/zenodo.10304481.

To quantify the dynamics of homologous recombination across different timescales, we analyzed shotgun metagenomic data from a collection of healthy human gut microbiomes that we collated in a previous study [27]. This data set comprises 932 fecal samples from 693 subjects from North America, Europe, and China (S1 Table). We used a reference-based approach to identify single-nucleotide variants (SNVs) in the core genome of each species in each sample (Section 1 in S1 Text). These metagenomic variants reflect a complex mixture of the global genetic diversity within a given species, as well as the specific combination of lineages that are present within a given host. While it is difficult to resolve the underlying lineages in the most general case, we previously showed [27] that the lineage structure in many human gut metagenomes is simple enough that the core genome of the dominant strain can be inferred with a high degree of confidence (Fig 1A and Fig A in S1 Text). Using this approach, we obtained a total of 5,416 “quasi-phased” genomes from 43 different species in 541 unique hosts. The genetic differences between these strains provide a window into the long-term evolutionary forces that operate in these species over multiple host colonization cycles.

Previous work has shown that the genetic diversity within many species of gut bacteria spans a broad range of timescales [27,45]. For example, in *Alistipes putredinis* (a prominent gut commensal), the synonymous divergence between a typical pair of strains is *d*≈2%, but some pairs of strains are separated by just a handful of SNVs (Fig 1C). Similar pairs of closely related strains have been observed in other bacterial species, where they are often associated with clinical outbreaks or other local transmission processes [21,36,46]. In our case, the breadth of sampling of the human gut microbiome allows us to rule out many of these microepidemic factors, since closely related strains are frequently observed in unrelated hosts from different countries [27].

Population genetic theory predicts that similar patterns can also arise due to the local nature of bacterial recombination [28,39]. Since the length of a typical recombined segment (ℓ_*r*_) is usually much shorter than the total genome length (*L*), there is a broad range of timescales between the first recombination event (*T*_*r*_) and the time required for the genome to be completely overwritten by imported fragments ($T_{mosaic}∼T_{r}\cdot L/l_{r}$; Fig 1B).

In quasi-sexual bacterial populations, most pairs of strains will share a common ancestor *T*_mrca_≫*T*_mosaic_ generations ago, so that their present-day genomes comprise a mosaic of overlapping recombination events. However, in a large enough sample, some pairs of strains will inevitably share a common ancestor on timescales much shorter than *T*_mosaic_ (Fig 1A). Among these “closely related” strains, recombination will not have had enough time to completely cover the ancestral genome with DNA from other, more typically diverged strains. Rather, individual recombination events will be visible as “blocks” of typical genetic divergence against a backdrop of nearly identical DNA sequence [28,39]. These partially recombined genomes have previously been observed in other bacterial species—most notably in *Escherichia coli* [28,39] and other bacterial pathogens [42,47,48]. Fig 1D shows that similar examples can be observed within the *A*. *putredinis* population as well.

To test whether this pattern holds more broadly, we divided the core genome of each pair of strains into blocks of 1,000 synonymous sites and calculated the fraction of blocks with zero SNV differences within them. In a pair of partially recombined genomes, we would expect to see a negative correlation between the fraction of identical blocks (a proxy for the fraction of clonal ancestry) and the overall genetic divergence across the genome (Fig 1E and Fig B in S1 Text). One can observe such a trend in *A*. *putredinis* (Fig 1C)—well beyond that expected from the randomness of individual mutations. Instead, we find that a simple model of accumulated transfers (red line; Section 2 in S1 Text) can account for a large fraction of the spread in genome-wide divergence in *A*. *putredinis*, consistent with the partial recombination model in Fig 1B.

Similar patterns can be observed in many other gut commensals (Figs C and D in S1 Text). Some species exhibit some variation in the divergence of the most distantly related strains (e.g., *Bacteroides vulgatus* and *Eubacterium rectale*), consistent with the presence of subspecies or other forms of population structure [18,27,49]. Yet even in these cases, we find that partially recombined genomes can still account for much of the variation among more closely related strains. Across species, we find that our simple model of accumulated transfers can explain more than 50% of the weighted variation in pairwise divergence within 36 of the 43 species we examined (Fig F in S1 Text). The implied amounts of recombination are often quite large (*T*_mrca_/*T*_mosaic_≳10, Fig 1F) and are comparable to highly recombinant species like *Helicobacter pylori*. These estimates suggest that typical pairs of strains have been completely overwritten by recombination events (Fig 1B).

Despite the generality of this trend, the total number of closely related strains can vary substantially between species (S2 Table). For example, many *Alistipes* and *Bacteroides* species contain hundreds of closely related pairs, while other species like *Prevotella copri* have only a handful. While the causes of these differences are currently unclear, the simplified patterns of recombination among these strains suggest that we can use them to directly resolve individual recombination events within a range of different species.

### Measuring individual recombination events that accumulate between closely related strains in different hosts

To identify individual recombination events across a diverse range of human gut species, we turned to an automated approach for analyzing the spatial distribution of genetic differences along the core genomes of closely related pairs of strains. We chose to focus on the core genome to limit the impact of plasmids and other mobile genetic elements, which can be horizontally transmitted at much higher rates than normal chromosomal DNA [50–52]. By restricting our attention to core genes, we sought to infer the baseline rates of recombination that shape the evolution of the larger genome, which involve the permanent replacement of existing sequences in addition to successful transfers.

**Fig 2:**
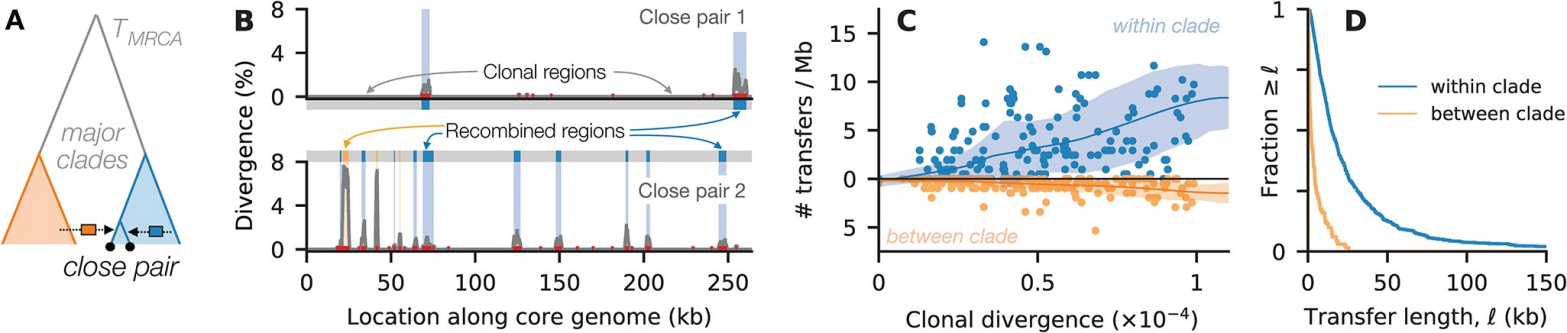
Measuring individual recombination events that accumulate between closely related strains. (A) Schematic illustration for *B*. *vulgatus*, which has a strong population structure with 2 major clades. (B) Our pairwise hidden Markov model (CP-HMM) classifies the core enome of each pair of closely related strains into clonal regions (gray) and recombined regions (blue = within-clade, orange = between-clade) based on their local synonymous divergence; points denote individual SNVs, while lines show the local divergence in sliding 1,000 bp windows. Data from 2 example pairs are shown. (C) The observed number of recombination events in all pairs of closely related *B*. *vulgatus* strains as a function of the synonymous divergence in their inferred clonal regions (Section 3.1 in S1 Text). These events are further partitioned into within-clade and between-clade transfers (top and bottom). Lines indicate the average trend computed using a local regression technique, while shaded regions indicate the local spread (Section 3.3 in S1 Text). (D) Distribution of the estimated transfer lengths for each of the recombination events in panel C. These data show that the rates and lengths of successful transfers strongly depend on the divergence of the imported fragments. The data underlying this figure can be found in https://doi.org/10.5281/zenodo.10304481.

Our pairwise model assumes that the genetic differences along the core genome arise through a mixture of 2 processes: (i) point mutations (which alter individual sites); and (ii) homologous recombination events (which replace longer stretches of DNA with a corresponding fragment sampled from another strain in the populations). For sufficiently close pairs, the mutation and recombination processes have a negligible chance of overlapping, which means that they can be captured by a simple hidden Markov model (HMM) that transitions between clonal and recombined regions at different locations along the genome (Fig 2B and Fig G in S1 Text; Section 3.1 in S1 Text). The corresponding transition rates between these states will vary between different pairs of strains, due to the differences in their time-aggregated rates of recombination. Since the genealogies of close pairs are particularly simple, these pairwise estimates can implicitly capture various forms of selection, non-equilibrium demography, and other deviations from the simplest neutral null models, even when there is insufficient data for a complete phylogenetic reconstruction.

In contrast to previous approaches [28–31,53], we used the empirical distribution of local divergence to model the number of SNVs imported by each recombined fragment (Section 3.1 in S1 Text). This allows us to capture the broad variation observed in different transfers (Fig H in S1 Text) in a way that is directly informed by the available data. We validated the performance of our algorithm (CP-HMM) through simulations and found that it can reliably identify individual recombination events across a range of genetic divergence scales (Figs I–L in S1 Text; Section 3.2 in S1 Text).

Fig 2 shows an example of this approach applied to *B*. *vulgatus*, one of the most abundant and prevalent species in the human gut. Previous work [27] has shown that this species possesses a strong population structure with 2 major clades (corresponding to the *vulgatus* and *dorei* subspecies [54]), such that the within-clade divergence is approximately 10-fold smaller than the divergence between clades (Fig 2A). We exploited this structure to further resolve the recombination events into within- and between-clade transfers based on their local sequence divergence (Fig 2B, Section 3.1 in S1 Text). By applying our HMM algorithm to the 210 pairs of closely related *B*. *vulgatus* strains in our cohort, we identified a total of ≈1,700 recombined regions with a mean length of ≈20 kb (S3 Table). We also applied our algorithm to a separate collection of *B*. *vulgatus* isolate genomes (Fig O in S1 Text; Section 3.10 in S1 Text) to verify that our conclusions were robust to the quasi-phasing approach employed in Fig 2.

We observed an overall trend toward larger numbers of recombination events in strains with higher clonal divergence (Fig 2C), consistent with the gradual accumulation of successful transfers over time. However, the larger sample reveals that this is not a simple linear relationship: Some strains have anomalously large numbers of transfers even at low clonal divergence, while others have anomalously few transfers even at high clonal divergence (Fig 2C). Similar results are also observed when considering the cumulative length of the recombined genome for each pair (Fig M in S1 Text), which confirms that this variation is not an artifact of the event detection algorithm. Instead, these data suggest that successful transfers in *B*. *vulgatus* do not accumulate at a fixed recombination rate, as assumed under the simplest models of neutral evolution.

We also found that recombination between the major *B*. *vulgatus* clades occurred much less frequently than recombination within clades, with a ~5-fold reduction in the total number of detected transfers as a function of their genetic divergence (Fig 2C and Fig J in S1 Text). This genetic isolation could arise from several factors, ranging from reduced opportunities for recombination (e.g., due to ecological isolation [2] or fewer homologous flanking regions for initiating strand invasion [55,56]) to greater downstream incompatibilities in the acquired fragments (e.g., epistatic interactions [57,58] or mismatch-repair-mediated proofreading [59,60]). In this case, the larger ensemble of detected transfers allows us to further distinguish between these scenarios. Beyond the reduction in the number of detected recombination events, we also observed a systematic difference in the lengths of the individual transfers, with a ~7-fold reduction in the median transfer length between clades (Fig 2D and Fig J in S1 Text). These differences indicate that the greater genetic isolation of the *B*. *vulgatus* clades cannot be captured by a simple rescaling of the recombination rate and that additional factors like epistasis or mismatch-repair-mediated proofreading are necessary to explain the data.

### Variation of recombination rates within and across gut species

**Fig 3:**
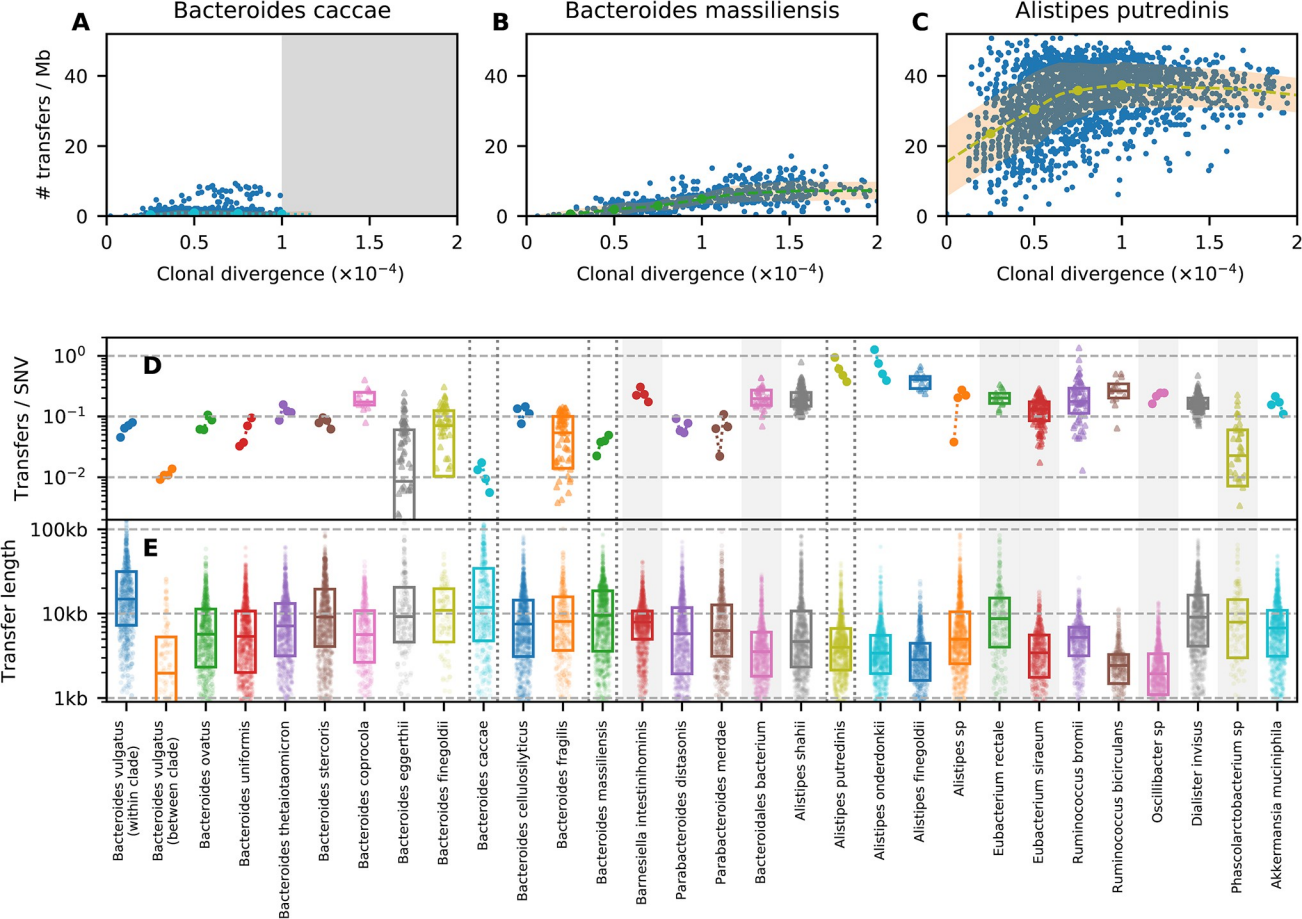
Heterogeneous recombination rates within and between prevalent gut species. (A–C) Analogous versions of Fig 2C for 3 example species, which were chosen to illustrate a range of characteristic behaviors. Gray regions denote the points that were excluded by our filtering steps (Section 3 in S1 Text). (D) Apparent recombination rates (number of transfers/clonal divergence/core genome length) for all species with a sufficient number of closely related strains (Section 3.3 in S1 Text). For species with > 100 close pairs, we plot the average recombination rate at 4 characteristic divergence times (*d*_*c*_ = 2.5,5,7,5,10×10^−5^, highlighted as points along the trend lines in panels A–C) using the trend lines in panels A–C; estimates are connected by lines to aid visualization. For species with < 100 close pairs, we plot the distribution of apparent recombination rates for all individual pairs; box plots indicate the median and inter-quartile range. (E) Lengths of recombined fragments for each of the species in panel D. Symbols show the lengths of all detected transfer events across all pairs of closely related strains; box plots indicate the median and inter-quartile range. The data underlying this figure can be found in https://doi.org/10.5281/zenodo.10304481.

To understand how these results for *B*. *vulgatus* extend to other members of the gut microbiome, we applied the same approach to the other species in our data set with a sufficient number of closely related strains. This pairwise analysis yielded a total of 228,078 recombined regions in 7,383 closely related pairs from 29 different species. These data revealed systematic variations in the rates and lengths of transferred fragments across many prevalent gut species (Fig 3 and Figs P–R in S1 Text), similar to *E*. *coli* and other bacterial pathogens [16,61–63].

We found that some of these trends were consistent with the phylogenetic relationships between species. For example, species in the Rikenellaceae family tended to have relatively frequent and short transfers, while Bacteroidaceae family tended to have lower rates and longer transfers. However, we also observed large differences within individual genera. For example, *Bacteroides massiliensis* has a relatively linear accumulation of transfers over time (Fig 3B), while most pairs of *Bacteroides caccae* strains have few detected recombination events (Fig 3A). The typical transfer length varies among *Bacteroides* species as well (6 to 35 kb), spanning a larger range than *Alistipes* (3 to 6 kb).

Zooming in further, we also observed considerable variation within individual species. Some of these differences could be attributed to the presence of strong population structure (similar to *B*. *vulgatus*), with a reduction in both the rates and lengths of successful transfers between highly diverged clades (e.g., *Alistipes Shahii*; Fig V in S1 Text). However, we also observed substantial variation even in the absence of population structure. For example, *A*. *putredinis* contains many closely related strains with an anomalously large number of transfers, as well as an excess of more diverged strains with few recombined segments (Fig 3C and Fig K in S1 Text). Other species (e.g., *B*. *caccae*; Fig 3A) exhibited bimodal distributions of transferred fragments. None of these behaviors can be captured by a single underlying recombination rate.

**Fig 4:**
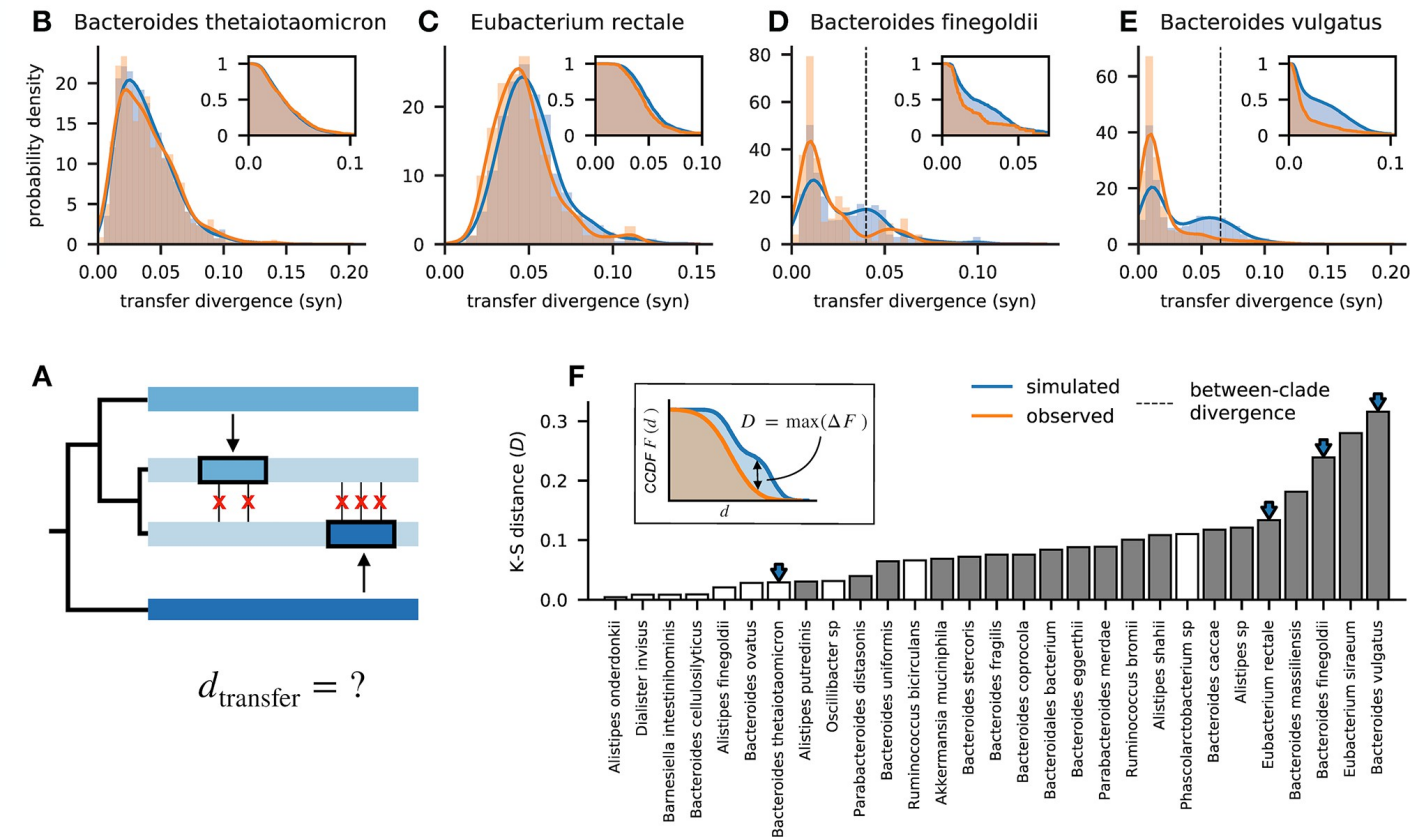
Quantifying the frequency of recombination as a function of the genetic divergence between donor and recipient DNA sequences. (A) Schematic illustration showing the genetic divergence of 2 recombined fragments relative to the focal pair of genomes. The synonymous divergence of each detected transfer is computed and aggregated across all closely related pairs within a species. (B–E) Distribution of donor-recipient divergence for all detected transfers in 4 example species. Orange lines show the observed data, while the blue lines show a null expectation obtained by randomly drawing segments from the observed collection of genomes (Section 3.6 in S1 Text). Insets show the corresponding complementary cumulative distribution functions. For species with a strong clade structure (D and E), the average between-clade divergence is indicated by dashed vertical lines. (F) Differences between the observed and simulated divergence distributions for all of the species in Fig 3, summarized by the Kolmogorov–Smirnov (K-S) distance (inset). Solid bars indicate statistically significant differences (*P*<10^−3^; one-sided K-S test), while arrows indicate the example species in panels B–E. Together, these data show that many species exhibit only small differences between their observed and expected divergence distributions (K-S distance ≲0.1), even when their overall sequence divergence is comparable to counterexamples like D and E (Figs W and X in S1 Text). The data underlying this figure can be found in https://doi.org/10.5281/zenodo.10304481.

Interestingly, apart from the handful of species with strong population structure, we observed no systematic trend between the frequency of recombination and the divergence of the transferred fragments (Fig 4 and Fig W in S1 Text), as expected under certain models of homologous recombination [19,64]. This observation, in combination with the large number of species in our data set, helps shed further light on the mechanisms that could be responsible for the lower recombination rates we observe between clades. For example, *B*. *thetaiotaomicron* and *B*. *stercoris* both maintain high recombination rates at synonymous divergences comparable to the genetically isolated clades observed in *B*. *vulgatus* and *B*. *finegoldii* (Fig 4 and Fig W in S1 Text). This suggests that the genetic isolation of these clades is not a product of their underlying recombination machinery (which should be similar in different *Bacteroides* species) but rather by genetic incompatibilities that have accumulated between the 2 clades, or related scenarios like incompatible restriction-modification systems [65–68]. Understanding the ecological and evolutionary forces that caused these incompatibilities to emerge within some *Bacteroides* species but not others is an interesting avenue for future work.

### Signatures of within-host recombination in co-colonized hosts

Our preceding analysis focused on the successful transfers that have accumulated between closely related strains in unrelated hosts. How do these long-term dynamics—which aggregate over multiple host colonization cycles—emerge from the local processes of competition and colonization within individual hosts?

**Fig 5:**
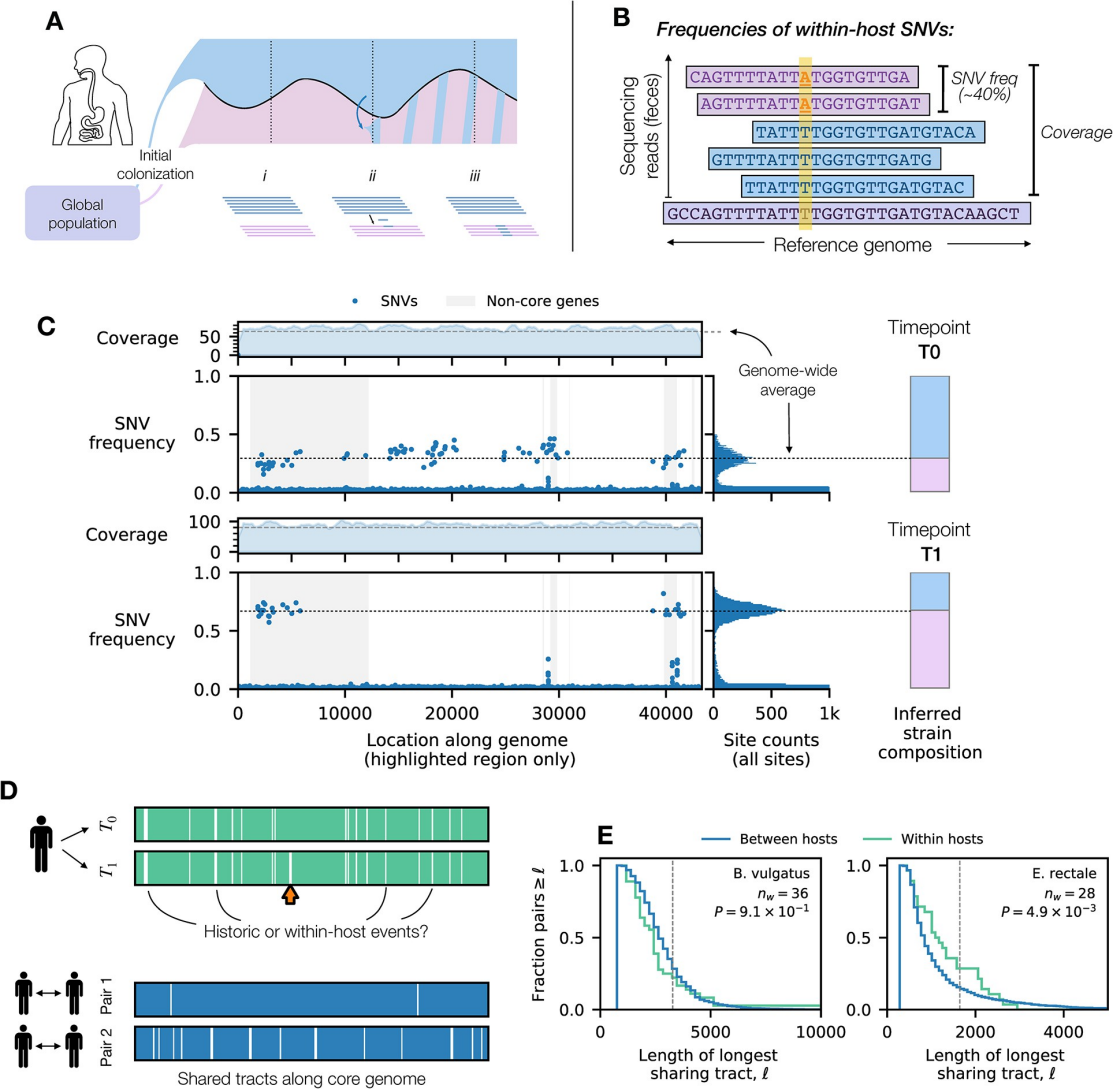
Signatures of within-host recombination among co-colonizing strains. (A) Schematic illustration of a potential recombination scenario: (i) a single host is colonized by a pair of diverged strains; (ii) recombination generates hybrid strains that initially reside at low frequencies; (iii) if a hybrid replaces its parent (e.g., due to a selective sweep), it will lead to a depletion of genetic diversity within the transferred region. (B) Within host SNVs are identified by aligning metagenomic sequencing reads to the reference genome. The frequencies and coverages of these SNVs can be used to identify gene-specific sweeps by hybrid strains (Fig OO in S1 Text). (C) An example of a hybrid sweep in a *B*. *vulgatus* population in a longitudinally sampled host. Top and bottom parts show metagenomic data collected from the same host at timepoints *T*_0_ (top) and *T*_1_ (bottom); Δ*t*~6 mo. In the top panel of each timepoint, solid lines denote the local coverage, estimated from a moving average of the local read depth. In the bottom panel of each timepoint, symbols denote the frequencies of within-host SNVs in the highlighted region of panel D (orange arrow), which are polarized such that the reference alleles have frequency >0.5 at *T*_0_; for comparison, the genome-wide distribution of SNV frequencies is shown on the right, illustrating the coexistence between 2 dominant strains at both timepoints (black dotted lines, bar plots). Gray regions denote non-core genes. These data show a sudden depletion of SNVs within a ~20 kb region. The consistent coverage around the genome-wide average (gray dashed lines) at both timepoints indicates that the depletion of SNVs in the highlighted regions is not caused by large deletion in one of the coexisting strains. (D) Tracts of shared ancestry between *B*. *vulgatus* strains. Top panels show the spatial distribution of within-host SNVs (green vertical lines) and tracts of shared ancetry (white regions of 0 SNV) from the host in (C); orange arrow highlights the putative within-host sweep event in (C). For comparison, the bottom 2 panels show analogous distributions computed for pairs of strains from different hosts. In these examples, long sharing tracts similar to the within-host sweep in (C) are visible along the genome. (E) Distribution of the longest sharing tract in each co-colonized host for 2 example species (Section 4.3 in S1 Text). Gray dashed lines indicate the mean transfer length inferred in Fig 3E. The total number of co-colonized samples and the *P*-value under the one-sided Kolmogorov–Smirnov test are shown. The *B*. *vulgatus* distribution is not significantly different from its between-host counterpart, while *E*. *rectale* displays a significantly higher rate of within-host sharing. The data underlying this figure can be found in https://doi.org/10.5281/zenodo.10304481.

Some of this recombination could occur when multiple strains of the same species are present within the same host [69]. While examples of co-colonization are less common in the human gut [27,45], we can still identify many individual hosts in our larger cohort in which 2 diverged strains were present at intermediate frequencies, based on the frequencies of SNVs within their corresponding metagenomes (Fig A in S1 Text). Recombination between these strains will generate hybrid genomes that contain a short fragment from their donor (Fig 5A). Each of these hybrid strains will originate as a single cell and will not be visible in a mixed sample unless they later rise to appreciable frequencies. Such a shift could occur through a single-cell bottleneck, e.g., if the hybrid strain is lucky enough to found a new population in naive host. Alternatively, if the transferred fragment provides a fitness benefit to the recipient strain, it can rapidly increase in frequency within its host and eventually displace its parent. These “gene-specific sweeps” will lead to a characteristic depletion of SNVs within the donated region in a mixed population sample, while preserving the remaining genetic variation elsewhere along the genome (Fig 5A). The higher frequencies of the resulting hybrids will make them substantially more likely to seed future colonization events in other hosts, suggesting that they could play an important role in generating the recombination events we observed in Figs 2 and 3.

Fig 5 shows an example of this scenario in a longitudinally sampled host who was co-colonized by a pair of typically diverged *B*. *vulgatus* strains (*d*≈1%). We observed a sudden depletion of within-host SNVs within a ~20 kb region during the ~6-month interval between samples (Fig 5B and 5C), while the SNV patterns across the rest of the genome were largely preserved. This local depletion of diversity cannot be explained by a large deletion event in one of the 2 strains, since the estimated copy number of the recombined region remained close to one at both timepoints (Fig 5C and Fig Y in S1 Text). This region spanned a total of 25 core and accessory genes on the reference genome, including a resistance-nodulation-division (RND) family efflux pump (S5 Table and Fig EE in S1 Text); at present, it is not clear which of these genes was responsible for driving the sweep, or if the recombined fragment was simply hitchhiking alongside a different causative mutation.

With limited longitudinal data from co-colonized hosts, it is difficult to find many contemporaneous examples like the one illustrated above. However, we reasoned that the remnants of these gene-specific sweeps would still be visible even in metagenomic data from a single timepoint. Previous work suggests that conspecific strains can coexist within their hosts for years at a time [27,70,71]. Any gene-specific sweeps that occur during this interval will produce an extended run of zero SNVs against the backdrop of an otherwise diverse metagenome. We identified many such runs of shared ancestry among the co-colonized hosts in our cohort (Section 4.2 in S1 Text), including several other examples in the *B*. *vulgatus* population above (Fig 5D). These runs can extend for thousands of base pairs and are significantly longer than we would expect if the mutations were randomly scattered across the genome (*P*<10^−10^, Fig Z in S1 Text). This suggests that they could be candidates for previous gene-specific sweeps that occurred within the host’s lifetime.

However, it is important to distinguish this scenario from older recombination events that were inherited by the strains before they colonized their current host (Fig 5A). Estimates suggest that a 10 kb fragment will require hundreds of years on average to accumulate its first mutation [72], which implies that any given run could be consistent with a broad range of possible ages. Consistent with this expectation, we also observed many long runs of shared ancestry when comparing strains from unrelated hosts—some of which extended for as long as the within-host examples above (Fig 5D and Fig Z in S1 Text).

This suggests that the true signal of within-host recombination must be distinguished from this baseline level of sharing. We reasoned that if within-host recombination was prevalent, we should still expect to see longer runs of shared ancestry in co-colonizing strains compared to random pairs of strains obtained from unrelated hosts. To test this idea, we used the length of the longest run as a test statistic, and asked how the distribution of this quantity differed between co-colonizing strains of the same species and random pairs of strains selected from unrelated hosts.

We observed a strong enrichment of long runs in co-colonizing strains of *E*. *rectale* (Fig 5E), which suggests that they were likely caused by previous within-host recombination events similar to the *B*. *vulgatus* example above. Similar results were obtained when we examined the total length of runs that exceeded a given length threshold (Fig AA in S1 Text).

In contrast, we found that some of the other species with high rates of recombination across hosts (e.g., *A*. *putredinis*; Fig 3C) did not show any enrichment in within-host sharing (Fig AA in S1 Text). This negative result could imply that co-colonizing strains recombine less frequently in these species or that fewer hybrid strains manage to sweep to high frequencies. It could also occur if the background levels of between-host sharing are sufficiently frequent that they overwhelm any signature of within-host sweeps. This scenario could be particularly relevant for species like *B*. *vulgatus* (Fig 5), in which nearly half of all random strain pairs share identical sequences longer than the typical transfer length in Fig 2. These results show how understanding the population genetic patterns between hosts can be important for resolving the evolutionary forces within individual host communities.

### Distribution of shared DNA segments across hosts reveals selection on recent transfers

The high levels of between-host sharing in species like *B*. *vulgatus* raise a natural question: Why do random pairs of strains share so many stretches of identical DNA within their core genomes? Population genetic theory predicts that such tracts of shared ancestry can emerge even in simple neutral scenarios due to the joint action of recombination, mutation, and genetic drift [73]. For a random pair of strains, the expected number of shared fragments longer than ℓ scales as $∼L/\bar{d}l^{2}(1+r/\mu {)}^{2}$, where $\bar{d}$ is the average divergence between typical pairs of strains (Fig CC in S1 Text; Section 5.1 in S1 Text). The slow decay with ℓ and *r* implies that this number will often be larger than one, even for tracts as long as ℓ~10 kb. This suggests that the presence of shared segments alone is not surprising.

**Fig 6:**
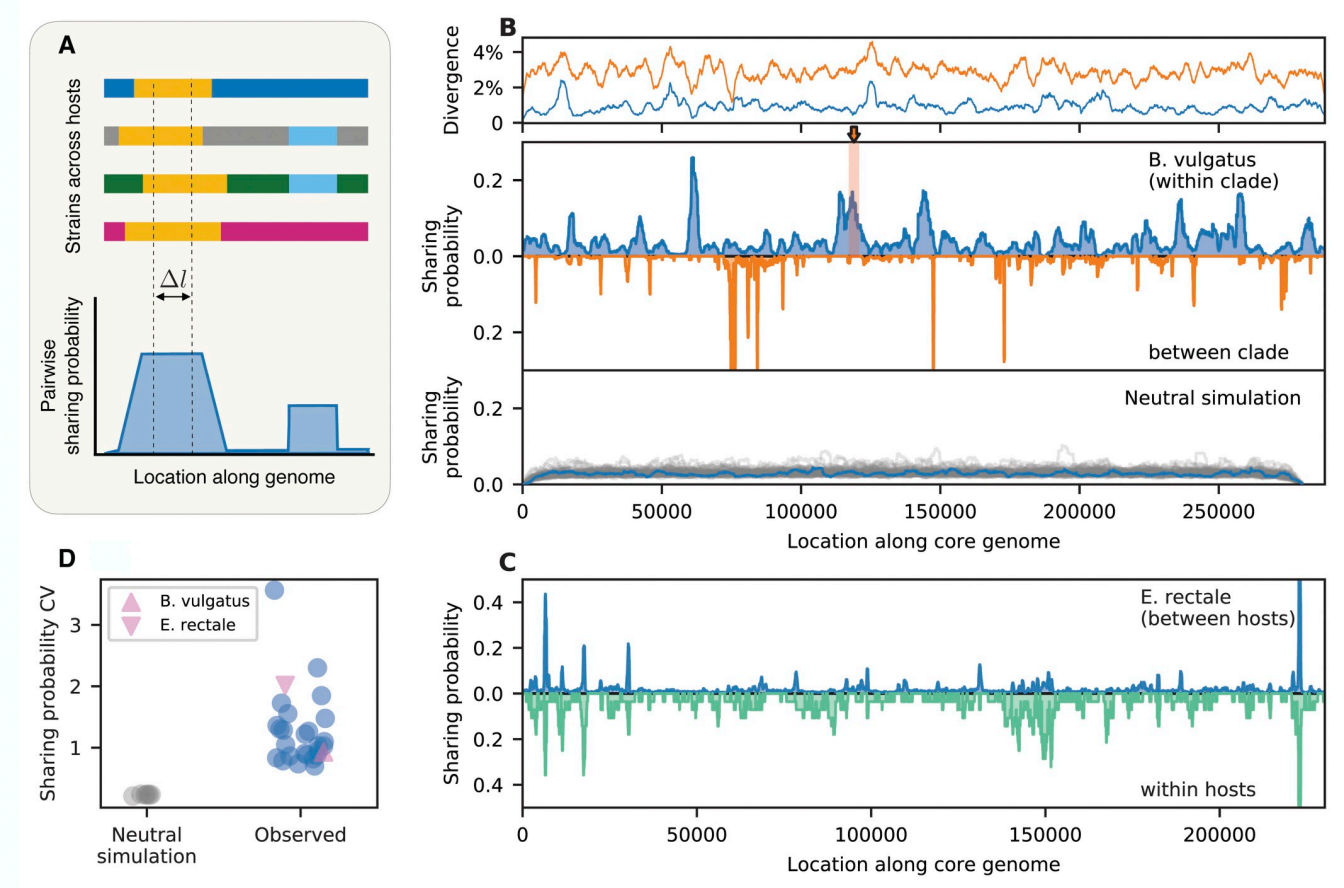
Global distribution of shared DNA segments reveals selection on recent transfers. (A) Schematic of pairwise haplotype sharing metric: For each position in the core genome, we compute the fraction of strain pairs from different hosts that have identical genotypes across a window of $\Delta l\gg 1/\bar{d}$ synonymous sites (Section 5.2 in S1 Text). (B) Observed sharing landscape for *B*. *vulgatus* (middle panel); separate comparisons are performed for strains from the same clade (blue, Δℓ≈1,500 synonymous sites ≈ 10 kb) or different clades (orange, Δℓ≈220 synonymous sites ≈ 1.5 kb). The top panel shows the average synonymous divergence computed in sliding windows of size Δ*l* = 3,000. These landscapes reveal regions of elevated sharing across hosts (e.g., shaded region) that cannot be explained by local reductions in diversity. Red shaded region indicates the within-host recombination event in Fig 5B and 5C. The bottom panel shows analogous sharing landscapes from neutral simulations (Section 5.3 in S1 Text), which display more even rates of sharing across the genome. Gray lines denote 100 simulation runs with the same parameters, while the blue line highlights 1 typical run. (C) Sharing landscape for *E*. *rectale*, computed for pairs of strains in different hosts (top) and co-colonizing strains from the same host (bottom). (D) Heterogeneous sharing landscapes across 27 species. Blue points show the coefficient of variation of the sharing probability across the genome for all species with sufficient between-host comparisons. *B*. *vulgatus* (within clade) and *E*. *rectale* are highlighted as pink triangles. Gray points show analogous values derived from neutral simulations across a range of parameter values (Section 5.3 in S1 Text); each point denotes the mean of 100 simulation runs, while lines show the standard deviation. The data underlying this figure can be found in https://doi.org/10.5281/zenodo.10304481.

However, this simple neutral scenario makes strong predictions about how often a given region is shared across multiple pairs of strains. To test whether this scenario could recapitulate our data, we scanned across the genome of each species and calculated the probability that each position was involved in a long shared segment ($l\cdot \bar{d}>15$, Fig 6A, Section 5.2 in S1 Text). This analysis revealed a systematic variation in the probability of shared segments at different genomic locations (Fig 6B–6D).

An example of this behavior is shown for *B*. *vulgatus* in Fig 6B. A typical site in the *B*. *vulgatus* genome has a 3% chance of being shared in a segment longer than approximately 10 kb. However, we observed that many local regions were shared much more frequently than the genome-wide average, despite having comparable levels of genetic diversity (Fig 6B and Fig DD in S1 Text). Some of these peaks are driven by the expansion of a single dominant haplotype, while others correspond to multiple distinct haplotypes that are shared by different sets of strains (Figs FF and GG in S1 Text). Similar “sharing hotspots” can be observed in other prevalent gut species as well (Fig 6C and 6D).

This high degree of heterogeneity is inconsistent with simple neutral models of bacterial evolution. Simulations show that neutral models generate significantly tighter correlations between the average and maximum levels of sharing across the genome (*P*<10^−8^; Student’s *t* test; Fig 6B and 6D and Fig HH in S1 Text). We also asked whether this heterogeneity could be explained by varying recombination rates along the genome [51,74,75]. However, our simulations showed that the sharing hot spots in Fig 6 are qualitatively distinct from traditional recombination hot spots. Local increases in the recombination rate actually decreased the probability of sharing longer segments (Fig II in S1 Text), since recombination tends to produce larger numbers of haplotypes with different combinations of mutations. Consistent with this finding, we observe few systematic correlations between the haplotype sharing landscapes in Fig 6 and the recombination hot spots inferred from Fig 3 (Fig JJ in S1 Text).

These analyses suggest that the heterogeneous sharing probabilities in Fig 6B are likely driven by positive selection on fragments that are spreading through the population via recombination. Consistent with this hypothesis, we found that the regions with the highest levels of sharing are statistically enriched for certain functional genes (e.g., glycosyltransferases) that have previously been shown to be under selection in the gut [72] (Section 5.5 in S1 Text). We also found that the sharing landscape qualitatively differs for fragments that are shared within versus between the major *B*. *vulgatus* clades (Fig 6B). This provides further evidence that the selection pressures are specific to the identities of the donated and recipient DNA sequences.

Finally, we asked how these global selection pressures were related to the within-host sweeps we detected in Fig 5. For example, we found that the within-host sweep event in Fig 5C occurred within one of the most prominent sharing hotspots in *B*. *vulgatus* (Fig 6B), which is peaked around 3 RND efflux pump genes (Fig EE in S1 Text). This suggests that both events were likely driven by a common set of selection pressures. However, this parallelism did not arise through selection of the same DNA sequences: while the sweeping haplotype in Fig 5C was also present in a few other hosts in our panel, we found that several other distinct haplotypes contributed to the global sharing hotspot at this location (Fig FF in S1 Text). This suggests that natural selection has promoted the transfer of multiple genetic variants at these loci—similar to a soft selective sweep [76].

Even larger differences were observed within the *E*. *rectale* populations in Fig 5E. In this case, while we observed some overlap in the sharing hotspots within versus between hosts, we also identified several new hotspots that were only present among co-colonizing strains (Fig 6C, Fig KK in S1 Text). These significant differences in the locations of the within-host sharing events (*P*<0.001, permutation test; Section 5.4 in S1 Text) provide further evidence that they were likely driven by selection on recent transfers within their hosts. More broadly, these results show that within-host sweeps are not always local versions of ongoing global sweeps, but may reflect distinct and repeatable selection pressures that are specific to the within-host environment (e.g., competition- versus colonization-related traits [77]). Understanding the tradeoffs that give rise to these different selection pressures is an interesting topic for future work.

## Discussion

Recombination is a ubiquitous force in bacterial evolution, but dynamics of this process are still poorly understood in many natural microbial populations. Here, we sought to quantify these dynamics by leveraging the broad range of timescales inherent in the human gut microbiome ecosystem. By analyzing recent recombination events within a panel of 29 gut commensals, we were able to identify general trends across diverse bacterial species that inhabit a common host-associated environment.

At a birds-eye view, the rates of recombination we observed across hosts (Fig 3) are comparable to other bacterial species [25,33,47] and are consistent with the strong decay in linkage disequilibrium that has been observed in global samples of gut bacteria [27,78]. Across species, we found that recombination is responsible for introducing >10-fold as much variation as mutation (*T*_mrca_/*T*_mosaic_≳10; Fig R in S1 Text), which implies that the genomes of typical circulating strains are almost completely overwritten by recombination. These values are broadly consistent with previous observations in bacterial pathogens, though their different sampling strategies can make it difficult to perform detailed numerical comparisons (Fig LL in S1 Text; Section 3.10.1 in S1 Text). The observation of such high rates of genetic exchange in commensal gut bacteria poses challenges for efforts to identify signals of parallel evolution in strains sampled from different hosts [72], or signals of codiversification across host populations [79,80], since they imply that individual variants can frequently decouple from the genome-wide phylogeny. In this case, more elaborate methods like the haplotype sharing metric in Fig 6 could be useful for resolving common selection pressures across hosts.

Although the long-term recombination rates in Fig 3 represent an average over multiple host colonization cycles, it is useful to consider their implications when extrapolated down to the scale of a single host community. If we assume the recombination events in Fig 3 accumulate largely neutrally (or via neutral hitchhiking [81]), then the rates implied by these data suggest that every site in the genome will be involved in more than a thousand recombination events within a single day (Section 3.8 in S1 Text). These ballpark estimates suggest that there will be numerous opportunities for adaptive mutations to spread between co-colonizing strains within a host (e.g., during a fecal microbiota transplant), even if the donor or recipient strain is present at a low frequency (e.g., approximately 0.1%). However, since each recombination event originates in a single cell, it can still take tens of thousands of generations (approximately 5 to 50 years) before a typical ancestral lineage will be involved in a single de novo recombination event. The large gap between these timescales can help explain why recombination can be an important driver of adaptation in the gut (Fig 5) [27], while also preserving the largely clonal structure observed in individual host populations [27,45,70,82]. We emphasize that these extrapolations should be treated with a degree of caution, since they assume that most of the recombination events in Fig 3 are effectively neutral. If the vast majority of these events were locally adaptive, then the true rate of recombination could be smaller than the apparent rates in Fig 3 (Section 3.8 in S1 Text).

In addition to the overall rates, the enhanced resolution of our approach also provided new insights into the dynamics of recombination within the gut microbiota. Extending previous findings in other bacterial species [28,61,83–85] (see [16] for a review), we observed widespread strain-level variation in recombination rates within many commensal gut species—at least some of which could be attributed to existing population structure (e.g., “subspecies” [49] or “ecotypes” [86]). In these handful of examples, the comparative nature of our data set helps illuminate the potential causes of this genetic isolation. By comparing the rates and lengths of successful transfers in species with different levels of genetic diversity, we obtained new evidence that the barriers to recombination are likely driven by negative selection on the recombined fragments (e.g., due to genetic incompatibilities), or related scenarios like incompatible restriction-modification systems [65–68], rather than passive mechanisms like ecological isolation or homology-dependent recombination rates (Fig 4). Our results suggest that understanding the causes and extent of these incompatibilities will be important for predicting the genetic cohesion and structure of bacterial species.

While our underlying approach relied on the presence of closely related strains to resolve individual recombination events, the widespread occurrence of these partially recombined genomes is still an interesting evolutionary puzzle. We previously showed [27] that the ecological structure of the human gut microbiome allows us to rule out common sampling biases (e.g., microepidemics or clonal blooms) that have been conjectured to play a role in other microbial species [21,36,46]. We also observed considerable variation across different commensal gut bacteria, with more than a quarter of the species in our panel containing just a handful of closely related strains from unrelated hosts. How could the same sample of hosts generate such a broad range of closely related strains in different species? The simplest neutral models predict a characteristic relationship between the mosaic timescale (*T*_mosaic_/*T*_mrca_) and the fraction of partially recombined genome pairs in the sample (Fig MMB in S1 Text) [42]. However, we found that the observed fractions are often much higher than this baseline expectation and show little correlation with the estimated recombination rates (Fig MMA in S1 Text). This suggests that new evolutionary models will be necessary to understand this puzzling feature of many natural bacterial populations.

Our results suggest that at least some of the long-term recombination dynamics across hosts arise from within-host sweeps of transferred fragments in hosts with multiple co-colonizing strains. This could provide a potential mechanism for the strain-level variation in recombination rates we observed in many species, since both the colonization structure and propensity for sweeps can vary dramatically in different hosts [27,70,82,87]. It remains unclear whether non-sweeping transfers could also play an important role in generating the long-term rates of recombination across hosts. Our results highlight the challenges involved in detecting these events, since we found that even unrelated strains can frequently share long stretches of DNA that are likely spreading through the global population via natural selection. These scenarios could potentially be distinguished with denser longitudinal sampling or larger samples of clonal isolates (e.g., using single-cell techniques [88]), which would allow us to distinguish between preexisting and in situ transfers [69].

While our present data do not provide direct information about the underlying mechanisms of horizontal DNA exchange in these species, our findings impose some interesting constraints on the potential mechanisms that might be involved. Many of the species in our panel (e.g., *Bacteroides*) are not known to be naturally competent [89], but still have long-term recombination rates that are as high as other species that are (e.g., *Streptococcus pneumoniae* [47,90]). Many gut commensals are known to engage in conjugative transfer, both in vitro and in vivo [91]. However, the time required for bacterial conjugation carries a substantial opportunity cost in the high growth regimes of the large intestine, and would need to be ameliorated by a corresponding fitness benefit or residence in a privileged spatial location [92]. Moreover, we observe little correlation between the overall rates of recombination in different species and their frequency of apparent multi-colonization (Fig NN in S1 Text). This suggests that these and other mechanisms that require physical proximity between strains are not the major driver of the long-term recombination rates we observed across hosts. It is possible that other species (e.g., phage or another commensal bacterium in the larger gut community) could serve as intermediate vectors for horizontal transfer between strains that are physically segregated in different hosts. Such inter-species transfer events have recently been observed within individual gut microbiomes [3,11,14]. It remains to be seen whether the rates of this process are sufficient to generate the long-term recombination rates we observe within species.

An important limitation of our metagenomic approach is that it is primarily restricted to recombination events within the core genome. While this provides important information about the long-term rates of recombination within gut commensal species, it is possible that much of this core-genome hybridization could be driven by positive selection on linked accessory genes (e.g., antibiotic resistance genes). Future applications of our methods on growing collections of clonal isolates [93] could shed light on these functional targets of horizontal transfer [94], and thereby provide a fuller picture of the landscape of bacterial recombination within the gut microbiota.

## Supporting information

S1 Table Metadata of metagenomic samples used in this study. We analyzed a collection of 932 samples from 693 individuals, collated in a previous study [27]. This included samples from 250 individuals from the Human Microbiome Project (HMP) [95,96], 185 individuals from [97], 250 individuals from [98], and 8 individuals from [99]. Listed are the subject identifiers, sample identifiers, run accessions, country of the study, continent of the study, visit number, and study.
(TXT)

S2 Table Number of close pairs across species. This table contains statistics of closely related strains across 43 species in our cohort. For each species, we computed the fraction of identical genome blocks for all pairs of genomes from unique hosts and recorded the number of pairs with >20%, >50%, >80% identical blocks. This table also contains the number of genomes in each species (“num_qp_samples”). Some species (e.g., *Prevotella copri*, *Roseburia inulinivorans*) have substantially fewer closely related pairs than others with comparable number of genomes.
(CSV)

S3 Table Detected transfers in the closely related pairs of 29 species. This table contains all the locations and divergences of recombination transfers shown in Figs 2 and 3. Listed are the species names, sample identifiers for each pair of strains, if the transfer is between-clade (“Y,” “N,” “NA”), if the transfer is included in Fig 2 (“TRUE,” “FALSE”), divergences (all sites or synonymous sites only), locations of transferred regions, and if the transfer is a potential duplicate of other detected transfers (“TRUE,” “FALSE”) (see Section 3.9 in S1 Text).
(CSV)

S4 Table Species with high-quality dual-colonized samples. Listed are species with >5 high-quality dual-colonized samples that passed the filters described in Section 4.1 in S1 Text.
(CSV)

S5 Table Annotations for genes in the within-host sweep example of *Bacteroides vulgatus*. Listed here are genes involved in the within-host sweep example in Fig 5 that have within-host SNVs at the first time point. Gene annotations are downloaded from PATRIC [100].
(CSV)

S6 TableClonal divergence thresholds d* and clonal fraction thresholds $f_{c}^{*}$.Clonal fraction thresholds d* and clonal fraction thresholds $f_{c}^{*}$ for selecting close pairs in certain species (Section 3.3 in S1 Text).(CSV)

S7 Table Metadata of isolate genomes used in Section 3.10 in S1 Text. Listed are the species names, species types (commensal or pathogen), genome accessions, and other information compiled in the Unified Human Gastrointestinal Genome (UHGG) collection [93].
(CSV)

S1 Text Methods and supplemental information. (PDF)

## Abbreviations

HGT: horizontal gene transfer
HMM: hidden Markov model
RND: resistance-nodulation-division
SNV: single-nucleotide variant
