# Supplementary material for: Dynamics of bacterial recombination in the human gut microbiome
Source: PLoS Biol. 2024 Feb 8;22(2):e3002472. doi: 10.1371/journal.pbio.3002472 (PMC10852326; doi:10.1371/journal.pbio.3002472)
Supplement: S1 Text — (PDF) [file pbio.3002472.s008.pdf]

S1 Text: Supplemental Methods

Contents

Page

1 Metagenomic pipeline **2**

2 Identifying partially recombined genomes from pairwise diversity statistics **3**
2.1 Estimating the the ratio between T_{mrca} and T_{mosaic} . . . 5

3 Identifying individual recombination events from pairs of closely related strains **7**
3.1 Description of the CP-HMM method . . . 7
3.1.1 Relation to previous work. . . . 9
3.2 Validation on simulated data. . . . 9
3.3 Application to data from the gut microbiome . . . 10
3.3.1 Evaluating the effects of different sample sizes . . . 11
3.4 Correspondence between the number of transfers and the fraction of recombined genome . . 11
3.5 Comparing the recombination dynamics of *B. vulgatus* and *A. putredinis* to simulated data . 12
3.6 Estimating the divergence distribution of donor and recipient DNA sequences . . . 13
3.6.1 Estimating the reduction in the realized recombination rate as a function of sequence divergence . . . 14
3.7 Estimating the ratio between T_{mrca} and T_{mosaic} . . . 14
3.8 Estimating the number of recombination events in a single gut microbiome . . . 15
3.9 Deduplicating detected recombination events . . . 16
3.10 Application to isolate genomes . . . 17
3.10.1 Comparisons with previous studies of bacterial pathogens. . . . 17

4 Identifying within-host recombination among co-colonizing strains **18**
4.1 Sample selection . . . 18
4.2 Identifying shared genomic regions among co-colonizing strains . . . 20
4.3 Distinguishing within-host recombination from pre-existing sharing . . . 20

5 Identifying signatures of selection from the global distribution of recent transfers **21**
5.1 Neutral expectation for the probability of observing long shared fragments . . . 21
5.2 Quantifying parallelism in recent transfers . . . 22
5.2.1 Connections to selection tests based on haplotype homozygosity. . . . 23
5.3 Comparison to simulated data from simple neutral models . . . 23
5.4 Quantifying the differences in the sharing landscapes of co-colonizing strains . . . 24
5.5 Enrichment analysis of functional classes of genes . . . 25

1 Metagenomic pipeline

We utilized a collection of metagenomic data that was collated in a previous study [1]. This collection consists of 932 fecal samples from 693 subjects from North America, Europe, and China, some of whom were sequenced at 2-3 timepoints roughly 6 months apart. We analyzed these data using the same reference-based pipeline described in Ref. 1. In short, we used the MIDAS software package [9] to align the raw sequencing reads from each sample to a large collection of reference genomes representing different bacterial species. The distribution of coverage across each genome was used to estimate the relative abundances of each species in each sample, as well as the presence and absence of individual genes. Following Ref. 1, we defined the core genome of each species to be the subset of protein coding genes that were present in >90% of the samples in which the species could be reliably detected. We chose to focus on the core genome to limit the impact of plasmids and other mobile genetic elements, which can be horizontally transmitted at much higher rates than typical chromosomal DNA. By restricting our attention to core genes, we aimed to infer the baseline rates of homologous recombination across the entire genome, which are critical for understanding the genetic structure of bacterial populations [10].

We used MIDAS's `snps` module to identify single nucleotide variants (SNVs) in each species based on the raw read pileups in each sample. These initial SNVs were subsequently filtered based on their absolute and relative coverage, as well as their location along the genome, using the same procedures and parameters described in Ref. 1; to reduce the effects of reference bias, no additional filtering is done using the allele counts as this stage (see below). The end result of this pipeline is a list of coverage values (D_i) and alternate (non-reference) allele frequencies (f_i) at each genomic position in the reference genomes that are detected in a given sample. Filtered sites and other missing data are assigned a coverage value of $D = 0$. These lightly processed SNV frequencies served as the basis for all of our downstream analysis.

The frequencies of the SNVs within each sample provide information about the lineage structure of the within-host population (Fig A). Ref. 1 showed that the lineage structure in many samples is sufficiently simple that the genotype of the dominant lineage can be inferred with a high degree of confidence. We used this "quasi-phasing" approach to infer the genotypes of 5416 strains from 43 different species, using the same procedures and parameters described in Ref. 1. These quasi-phased genotypes served as the basis for all of our subsequent between-host comparisons. Importantly, our downstream analyses only consider sites with high quality genotype calls, regardless of their polarization. Treating the remaining sites as missing data (rather than reference alleles) ensures that our between-host comparisons do not sensitively depend on the choice of the reference genome used for read mapping.

In S1 Text 4, we describe the additional methods we developed for analyzing non-quasi-phaseable samples to quantify recent recombination events within hosts.

Note on species names: the analysis in this work was based on version 1.2 of the MIDAS reference genome database. More recent taxonomic conventions have split the *Bacteroides* genus into two genera, *Bacteroides* and *Phocaeicola* [11], which alters some of the species names in the MIDAS database. For consistency, we have continued to use the original MIDAS species names, but have listed the relevant translations in the table below for completeness:

MIDAS DB v1.2	NCBI Taxonomy Browser
Bacteroides coprocola	*Phocaeicola coprocola*
Bacteroides massiliensis	*Phocaeicola massiliensis*
Bacteroides plebeius	*Phocaeicola plebeius*
Bacteroides vulgatus	*Phocaeicola vulgatus*
Eubacterium eligens	*Lachnospira eligens*

2 Identifying partially recombined genomes from pairwise diversity statistics

To test whether the local recombination model in Fig 1A could explain the broad range of diversity in many gut species, we used a lightweight approach similar to Refs. [12, 13], which compares the joint distributions of two diversity statistics that can be directly estimated from pairwise comparisons between quasi-phaseable samples.

For each species, we first filtered the data to retain at most one quasi-phased strain per household. We then considered all pairwise comparisons between the strains in these unrelated hosts. For each pair of strains, we identified the subset of four-fold degenerate (4D) synonymous sites in the core genome that were covered in both samples. We used this subset of sites to compute the average divergence across the genome (d) and the fraction of multi-site "blocks" that contained zero SNVs (f). Based on the divergence scales in our data, we chose a block size of $l = 1000$ 4D sites, so that a typical gut bacterial genome contains 200-400 such blocks.

We compared these data to a null model in which SNVs accumulated independently and uniformly along the genome. This would be the case, e.g., in an asexual model with a uniform mutation rate along the genome. In this case, the independent accumulation of SNVs leads to a simple relationship between the average divergence (d) and expected fraction of identical blocks (f). The probability that a block of length l contains no SNVs is given by

$$P\{\text{no snvs}\} = (1-d)^l \approx e^{-dl}\,, \tag{S1}$$

where we have used the fact that $d \ll 1$ and $l \gg 1$. Since an individual genome usually contains many blocks, we can equate $P\{\text{no snps}\}$ with the expected fraction of identical blocks f, yielding

$$d_A(f) = -\frac{1}{l}\log f\,. \tag{S2}$$

This expression was used to plot the dashed lines in Fig. 1B, C, B.

Deviations from this null expectation can naturally arise in simple models of bacterial recombination (Fig 1A). For example, in the simplest model where we assume that each block is replaced by a recombination event at a constant rate R per generation, the probability that a block has not been modified by recombination is given by

$$P\{\text{no recomb.}\} = e^{-2RT}\,, \tag{S3}$$

where T is the coalescence time of the given pair of strains. Similarly, the probability that a block has not been modified by mutation is given by

$$P\{\text{no mut.}\} = e^{-2\mu l T}\,, \tag{S4}$$

where μ is the mutation rate per site per generation. Then, the expected fraction of identical blocks is given by $f = P\{\text{no recomb.}\}\cdot P\{\text{no mut.}\}$, while the fraction of recombined blocks is given by $f_r = 1 - P\{\text{no recomb.}\}$. If we additionally assume that the recombination events import segments of the same characteristic divergence, $\overline{d}$ (corresponding to the average pairwise divergence within the species), then a typical transfer event will introduce many more SNVs than mutation in the clonal regions. This suggests that we can approximate the genome-wide divergence d by the recombined regions alone:

$$\begin{aligned} d_{PR}(f) &\approx \overline{d} f_r = \overline{d}(1-e^{-2RT}) \\ &= \overline{d}(1-f^{\frac{R}{R+\mu l}}) \end{aligned} \tag{S5}$$

This provides a simple alternative model connecting the fraction of clonal blocks with the total genome-wide divergence. In practice, $\overline{d}$ can be measured directly from the average pairwise diversity of the species, while the compound parameter $\alpha \equiv \frac{R}{R+\mu l}$ can be fit using the estimator

$$\hat{\alpha} = \frac{\sum_i \log(1 - d_i/\overline{d})}{\sum_i \log(f_i)} , \tag{S6}$$

where d_i and f_i are the observed values for each pair that has nonzero f.

To examine the usefulness of these expressions, we simulated well-mixed, neutral populations with mutation and localized recombination (5.3), and calculated the same pairwise statistics across a range of parameter values (Fig B). We found that for small recombination rates or short transferred fragments, the joint distribution of d and f is well-described by Eq. (S2), while increasing either parameter will lead to larger deviations from this null model of random mutations. In simulations with higher recombination rates, the majority of pairs have a small fraction of identical blocks and cluster around the average pairwise divergence $\overline{d}$ (Fig B). We observed similar patterns in most of the gut bacterial species in our cohort, consistent with the picture of "quasi-sexual" species in Fig 1. For these parameters, we found that the recombination model in Eq. (S5) accurately captures the results of the simulations, with α reflecting the relative strength of recombination vs mutation. These results suggests that our theoretical expressions Eqs. (S2) and (S5) can be used in combination to determine whether the pairwise diversity of a population is better described by an asexual model or a partial recombination model.

Consistent with our simulations, we found that the smallest divergence values in many species were well-predicted by their corresponding fraction of identical blocks. To quantify how well this additional variable explained the variation in pairwise divergence within species, we fit the joint distribution of pairwise statistics with a modified version of Eq. (S5),

$$\hat{d}_{PR}(f) = \begin{cases} c_0(1 - f^{\alpha}) & \text{if } f > 0, \\ c_1 & \text{else,} \end{cases} \tag{S7}$$

where c_1 is the average divergence across all pairs with $f = 0$, and c_0 is the average divergence across all *within-clade* pairs with $f = 0$. The reason we treated the $f = 0$ case separately is that some species exhibit significant variation in pairwise divergence at $f = 0$ due to population structure, which is not included in the simple model above. After fitting the partial recombination model in Eq. (S7), we estimated the goodness of fit (R^2) using the standard formula,

$$1 - R^2 \equiv \frac{\sum_i (d_i - \hat{d}_{PR}(f_i))^2}{\sum_i (d_i - \overline{d})^2} , \tag{S8}$$

where d_i and f_i are the observed values for each pair, and $\overline{d}$ is the average divergence across all pairs.

We found that for some species (e.g. *Eubacterium rectale*), the large range of variation among close pairs is well explained by partial recombination model (Fig C), but R^2 is sometimes overwhelmed by the large number of points at high divergence values (whose variation arises from population structure rather than the amount of clonal inheritance). To better capture the power of this variable in explaining the *range* of divergences, we also computed a weighted goodness-of-fit statistic, R_Y^2, that gives each range of y values an equal weight. More specifically, we divide the entire y range into 500 bins (B_i), count the number of points in each y bin (n_b), and use the $1/n_b$ as the weight of each data point:

$$1 - R_Y^2 \equiv \frac{\sum_b \sum_{i \in B_b} \frac{1}{n_b}(d_i - \hat{d}_{PR}(f_i))^2}{\sum_b \sum_{i \in B_b} \frac{1}{n_b}(d_i - \overline{d})^2} . \tag{S9}$$

We found that this modified metric successfully captures the explanatory power of f for species like *E. rectale*. For most species, however, R_Y^2 is approximately the same as R^2.

Finally, the pairwise divergence distribution revealed that some species (e.g. *Bacteroides vulgatus*) had a strong population structure of two or more clearly separable clades (Fig C). For these species, we used the pairwise divergence matrix to cluster the strains into two major clades using the `linkage` and `fcluster` functions in the SciPy hierarchical clustering library [14]. We then computed analogous versions of R_Y^2 and R^2 that were restricted to strains from the largest clade (Fig F). Again, we found that explicitly removing the population structure in this way was essential for revealing the explanatory power of f in cases where our naive metrics would be overwhelmed by the large divergences that have accumulated between the major clades.

2.1 Estimating the the ratio between T_{mrca} and T_{mosaic}

The compound parameter α in the partial recombination model provides a simple way to roughly estimate the ratio between T_{mrca} and T_{mosaic}. Recall that T_{mosaic} is the time it takes for the genome of a strain to be completely overwritten by recombination events, while T_{mrca} is the average coalescence time between a pair of genomes. The ratio between these two timescales characterizes the degree of clonality or quasi-sexuality of a given bacterial population.

It is useful to connect this ratio to basic biological parameters in the context of a simple neutral model with a well-mixed population. Let μ and r be the per site per generation mutation and recombination rate, l_r the typical recombination length, L the genome length, and $\overline{d}$ is the typical pairwise divergence. Then, we can express T_{mrca} and T_{mosaic} in those parameters:

$$l_r r L T_{\text{mosaic}} \sim L \Rightarrow T_{\text{mosaic}} = \frac{1}{l_r \cdot r}\,, \tag{S10}$$

$$\mu T_{\text{mrca}} \sim \overline{d} \Rightarrow T_{\text{mrca}} \sim \frac{\overline{d}}{\mu}\,. \tag{S11}$$

Therefore, in this simplest neutral model, we have

$$\frac{T_{\text{mrca}}}{T_{\text{mosaic}}} = \frac{r \cdot \overline{d} \cdot l_r}{\mu}\,. \tag{S12}$$

One can also interpret this quantity as a ratio between the number of mutations obtained through recombination in a given generation vs the number of mutations obtained through mutation (often referred to as "r/m" in previous work [15, 16]). This quantity differs from the bare ratio r/μ by the additional factor $d \cdot l_r$, which represents the typical number of mutations introduced per recombination event.

Under the simple model above, we can use the definition of α to obtain an expression for estimating $T_{\text{mrca}}/T_{\text{mosaic}}$,

$$\frac{T_{\text{mrca}}}{T_{\text{mosaic}}} = \frac{r \cdot \overline{d} \cdot l_r}{\mu} \approx \frac{R \cdot \overline{d}}{\mu} = \frac{l\overline{d}}{1/\alpha - 1}\,. \tag{S13}$$

For each species where the partial recombination model explains the bulk of the variation in pairwise divergence ($R_Y^2 > 0.5$), we computed the estimated $T_{\text{mrca}}/T_{\text{mosaic}}$ using the fitted parameter α. The resulting estimates are shown in Fig 1F.

Application to *H. pylori* and *M. tuberculosis*. To provide reference points for the range of $T_{\text{mrca}}/T_{\text{mosaic}}$

values observed in prevalent gut commensals, we performed the same analysis on two well-studied pathogen species, *H. pylori* and *M. tuberculosis*. *H. pylori* is known to be highly recombinant [17], while *M. tuberculosis* is widely believed to be evolving mostly clonally [18].

We downloaded 2871 *H. pylori* genomes and 2000 *M. tuberculosis* genomes (downsampled from 6879) from RefSeq (`https://www.ncbi.nlm.nih.gov/refseq/`) using the command

```
datasets download genome taxon TAXON_ID --annotated --exclude-atypical \
    --assembly-source 'RefSeq'
```

We then computed pairwise ANI estimates for all pairs of genomes using FastANI [19] (Fig EA-B). Since computing the pairwise diversity statistics above (e.g. fraction of identical blocks) requires pairwise alignment, which is computationally more expensive than FastANI estimates, we sampled a subset of genome pairs that were uniformly distributed in their ANI values (Fig EA-B, inset). This sampling step ensures that the downsampled data still contains some closely related (and therefore partially recombined) pairs, which provide the greatest signal for our partial recombination model. Finally, we used "nucmer" in the MUMmer 4 package [20] to perform pairwise alignment of sequences and computed pairwise diversity statistics analogous to Fig 1C (Fig EC-D).

We inferred α and $T_{\mathrm{mrca}}/T_{\mathrm{mosaic}}$ values for *H. pylori* using the same procedure as for the gut commensals above. Since the genome-wide divergence in *M. tuberculosis* is no longer dominated by the recombined regions but rather by mutation, we first subtracted the expected divergence from mutation alone (Eq. S2) and then applied the same procedure to infer α. We found that *H. pylori* has an effective $T_{\mathrm{mrca}}/T_{\mathrm{mosaic}}$ much larger than 1, and *M. tuberculosis* has a $T_{\mathrm{mrca}}/T_{\mathrm{mosaic}}$ smaller than 1, consistent with previous estimates of their "r/m" (Fig 1F). This result shows that all but one gut bacterial species in our cohort are highly recombinant, more akin to *H. pylori* than to *M. tuberculosis*.

3 Identifying individual recombination events from pairs of closely related strains

To compare recombination dynamics across different species of gut bacteria, we developed a hidden Markov model method (CP-HMM) to automatically identify recombination events from the spatial divergence profiles of closely related strains (Fig 2B). This HMM approach is conceptually similar to the one employed in Ref. 13, but with important technical modifications that we describe in more detail below. These pairwise HMMs can be viewed as a lightweight version of existing phylogenetic approaches like ClonalFrameML [21] or Gubbins [22]. Since the genealogies of closely related pairs are particularly simple (Fig 1B), this pairwise approach can implicitly capture various forms of selection, non-equilibrium demography, and other deviations from the simple neutral models assumed in previous work, even when there is insufficient data for a complete phylogenetic reconstruction. This additional flexibility will be important for our analysis of the gut bacterial species below.

3.1 Description of the CP-HMM method

CP-HMM is designed to model the spatial divergence profiles observed between pairs of closely related strains. As in Fig 1, we focus on the subset of 4D synonymous sites in the core genome that are covered in both samples. To minimize the effects of correlated mutations (e.g. due to mapping artifacts or multi-nucleotide mutations [23]), we first coarse-grain the genome into blocks of $b = 10$ 4D sites and assign a binary label to each block (0 =“no SNVs in block”, 1=“one or more SNVs in block”). Since all species in our study have a typical heterozygosity less than 10%, we expect that normal regions of the genome will usually have fewer than one SNV per block, and thus will be minimally impacted by this coarse-graining scheme.

We assume that these coarse-grained divergence profiles can be described by a hidden Markov model with two classes of hidden states: a clonal state C and a finite number of recombined states $\{R_i\}$, each of which is associated with a corresponding pair of parameters, (λ_i, θ_i), representing the average length and average divergence of the recombined fragment. The transitions between the hidden states are governed by the sparse transition matrix:

$$P\{q_{t+1}|q_t\} = \begin{cases} \rho_c \cdot p_i & \text{if } q_t = C, q_{t+1} = R_i, \\ 1-\rho_c & \text{if } q_t = C, q_{t+1} = C, \\ 1-1/\lambda_i & \text{if } q_t = R_i, q_{t+1} = R_i, \\ 1/\lambda_i & \text{if } q_t = R_i, q_{t+1} = C, \\ 0 & \text{otherwise,} \end{cases} \tag{S14}$$

where ρ_c is the overall probability of transitioning to a recombined segment from a clonal region, and p_i is the probability that the recombined segment will be of type R_i (Fig G). In cases where the reference genome of the species contains multiple contigs, we assume that the hidden states in each contig are obtained from independent draws from the same transition matrix in Eq. (S14). Finally, given a sequence of hidden states $\{q_t\}$, the corresponding emission probabilities are

$$P\{e_t = 1|q_t\} = \begin{cases} 1-(1-\theta_c)^b & \text{if } q_t = C, \\ 1-(1-\theta_i)^b & \text{if } q_t = R_i, \end{cases} \tag{S15}$$

where θ_c can be interpreted as the average divergence within the clonal region.

These assumptions define a hidden Markov model for the coarse-grained divergence bins as a function of the underlying parameters ρ_c, θ_c, and $\{p_i, \lambda_i, \theta_i\}$. In practice, we found that the exact numerical values

of these parameters did not affect our main findings as long as they had the correct order of magnitude. Since different pairs of strains have diverged from each other for different amounts of time, the ρ_c and θ_c parameters will generally vary widely across strains, and must be inferred for each pair using the iterative approach described below. For simplicity, we assume that the remaining parameters, $\{p_i, \lambda_i, \theta_i\}$, are shared by all strains from the same species. We fixed these parameters using the following considerations.

We assumed that the average lengths were similar for all recombined states ($\lambda_i \approx \lambda$), with a few exceptions described below. Since this length scale is initially unknown, we developed an iterative approach to infer the relevant value of λ for each species in a self-consistent manner. Starting from an initial guess of $\lambda \approx 1000$, we ran the CP-HMM algorithm described below to identify an initial collection of recombination events from the data. We then updated λ based on the average length of the detected transfers in the first iteration. We repeated this process several times for each species until the λ estimates converged to a steady value. In practice, we found that these estimates converged relatively rapidly ($< 10\%$ change after two iterations).

The remaining parameters, $\{p_i, \theta_i\}$, can be viewed as a discrete approximation to the underlying probabilities of importing fragments with different levels of divergence. Given our large sample sizes, we assumed that this distribution could be approximated by the *empirical distribution* of local divergence values observed in random pairs of strains from our larger sample. Specifically, we divided the core genome into blocks of $\Delta\ell = 1000$ 4D sites, and calculated the frequency of local divergence values (in 40 equally spaced bins) across all pairs of strains in our sample. We note that these empirical distributions of local divergence are much broader than a Poisson distribution with the same mean (e.g. Fig H), which would be the null expectation assuming a constant local coalescence time. This discrepancy illustrates the complex genealogical history along a bacterial genome, as well as the necessity of using an empirically-derived estimate of $p(\theta)$.

For species with a strong population structure, such as *Bacteroides vulgatus*, $p(\theta)$ naturally separates into distinct distributions when conditioned on sampling pairs from the same clade or different clades (Fig H). In these cases, we utilized this clean separation to further divide the set of recombined states into two types, representing within-clade and between-clade recombination events, whose corresponding values of (p_i, θ_i) are given by one of the two empirical histograms. We also allowed the length scales λ_i to vary for within-clade vs between-clade events, and used the same iterative approach described above to estimate these parameters for each clade.

With the above information, the hidden Markov model is fully specified. For each pair of strains, CP-HMM infers a sequence of hidden states $\{\hat{q}_t\}$ and model parameters $(\hat{\rho}_c, \hat{\theta}_c)$ using standard HMM algorithms [24]. In principle, the ρ_c and θ_c parameters are related to the per-base pair recombination and mutation rates for the pair of strains, respectively. However, for reasons described below, we did not work with these global parameters directly, but instead sought to identify individual recombination events and clonal divergence values directly from the sequence of $\{\hat{q}_t, e_t\}$ values.

For example, we identified individual recombination events from this sequence by computing all continuous stretches of recombined states. To eliminate potential mapping artifacts, we excluded all events containing ≤ 50 synonymous sites, which are much shorter than the typical recombined segments (Fig 3). Such events could originate from misaligned indels that generate enough SNVs that would cause them to be mis-identified as short recombined segments. For each of the recombination events that passed this filter, we estimated the local synonymous divergence of the recombined fragment based on the total number of SNVs that it contains (Figs 4 & W).

Similarly, we defined the "clonal fraction" of the genome to be the total fraction of sites with $\hat{q}_t = C$. Since undetected recombination events can massively inflate the number of SNVs in the clonal regions, we estimated the clonal divergence using an approach that filters out these regions with anomalously high local SNV density. Specifically, we coarse-grained the clonal regions into blocks of 1000 synonymous sites, and computed the average clonal divergence using only those blocks with ≤ 2 SNVs. We found that this procedure

greatly improved the accuracy of estimated clonal divergence in our validation analysis below.

3.1.1 Relation to previous work.

Our CP-HMM model is most similar to the HMM used in the pairwise analysis in Ref. 13, but with a few important distinctions. First, our model utilizes a range of recombined states as opposed to a single one. A negative binomial distribution is used in Ref. 13 to account for the fact that recombined regions consist of mosaics of Poisson distributions with different rates. However, for species like *B. vulgatus*, a negative binomial distribution cannot capture the underlying bimodal distribution caused by population structure. We sought to overcome this problem by explicitly introducing a large number of recombined states, each of which corresponds to a different (Poisson) divergence rate. We took advantage of our large data set to fit these rates empirically from the non-closely related pairs as described above. This non-parametric approach provides a more flexible route for inferring the underlying divergence distribution of the transferred segments (Fig W), and can be naturally generalized to account for clades and other forms of population structure (Figs 2 & V).

Second, instead of coarse-graining the alignment into large (a few kb) blocks, our model can achieve nearly site-level classification. This level of resolution is important for estimating not only transfer lengths, but also clonal divergence times. In fact, there will be an artificial bias had we used much larger blocks. Consider a long transfer spanning multiple blocks, where blocks on the two ends are only partially covered by the transfer. The divergence of the two end blocks will typically be lower than the mean divergence in the transfer. These end blocks will have some probability of being classified as clonal sequence, which will drive up the estimated clonal divergence. Since every transfer is susceptible to this bias, the clonal divergence estimation can be artificially correlated with the number of detected transfers. The relationship between the number of transfers and the clonal divergence – a central piece of our analysis – can therefore appear to be artificially linear, masking the high degree of variation that we actually observed. This subtle mode of error necessitates our more fine-grained approach.

3.2 Validation on simulated data.

To validate our algorithm's performance, we designed a simple computational model to simulate mutations and recombination transfers accumulated between a pair of closely related strains. Similar to [15], our model simulates the spatial distribution of SNVs along the genome. To better match the format of the real data, we simulate only the synonymous sites on the core genome. Let L be the number of sites and T_{div} be the divergence time between the pair. As a simple model of recombination, we assumed a constant mutation rate μ and recombination rate r at each site along the genome, so that the total number of mutations and transfers will follow Poisson distributions with means $2\mu T_{div} L$ and $2r T_{div} L$, respectively.

After drawing the total number of mutations and transfers to introduce, we first introduced the mutations randomly along the genome, and then introduced the transfers. Since real SNV densities can vary along the genome due to local variation of mutation rates, selective pressure and recombination history, preserving this variation is crucial to evaluating the performance of the HMM. To account for such patterns, we sampled the sequence of the recombined fragment from one of the real genomes in our cohort. More specifically, we randomly chose one strain at the beginning of the simulation to serve as the fixed focal strain. Next, for each recombination event, a random other strain is chosen as the donor, a random site is chosen as the starting point, and a transfer length is drawn from an Exponential distribution with mean λ. The corresponding sequence of the donor strain will then overwrite the focal genome, introducing additional SNVs alongside the mutation events above. For simplicity, we do not model a circular genome, so a transfer stretching beyond the alignment's end will be truncated. Transfers and mutations are allowed to overlap.

Using the genomes of *B. vulgatus*, we first generated 256 simulated pairs in total for a range of 16 different values of T_{div}. To closely mimic the patterns of real data, we matched the recombination length and overall recombination rate to the inferred values from the within-clade pairs of *B. vulgatus* (which corresponds to $\lambda = 2600$ and $r/\mu = 0.65$). We applied our CP-HMM method to this simulated data and recorded the statistics of detected transfers and clonal divergences. In Fig I we compared our algorithm's results with ground truth. Panel (A-C) plot the CP-HMM results for the inferred clonal divergence, the number of detected transfers, and the inferred clonal fraction, all of which appear to be in excellent agreement with the ground truth. We also pooled the lengths of all detected transfers and verified that the distribution follows the input exponential distribution almost exactly (Fig ID).

The slight under-detection of recombination events at high divergence times (i.e., in more recombined genomes) can arise from two sources: (i) extremely closely related transfers that have few mutations and (ii) merging of overlapping transfers. Both sources will be become more significant for more diverged pairs as the number of sampled transfers increases. While our current divergence-based approach cannot detect the first class of events, it is possible that variation in the accessory genome could provide further insight into these close transfers; we leave such an analysis for future work. The second class of events can be alleviated by filtering highly diverged pairs. Any residual merged transfers will bias the distribution of transfer lengths. However, the close agreement in Fig ID suggests that the second issue is not significant over the range of divergence times we have considered. Based on these validation results, we decided to restrict our downstream analysis to pairs of strains in which the clonal fraction was $\gtrsim 75\%$ (see below). This restriction ensures that the CP-HMM algorithm should have similar detection efficiencies across species, even when the species have significantly different levels of average pairwise divergence.

By using the empirical distribution of local divergence as a prior, we have implicitly assumed that the donor individuals are uniformly sampled from the (sequenced) population. To evaluate whether our results were sensitive to this assumption, we artificially increased the prior probability for between-clade transfers in *Bacteroides vulgatus* by 5 fold and reran the CP-HMM algorithm. We found that the signal in the data was sufficiently strong to overwhelm this change in prior, yielding quantitatively similar results compared to what we observed before. In particular, the median transfer lengths differed by only $\approx 10\%$, and we still detected ~ 5 times more within-clade transfers than between-clade transfers. Some within-clade transfers are no longer detected because of the lowered prior probability. Our method for estimating clonal divergence has taken this potential under-detection into account, and the resulting $T_{\mathrm{mrca}}/T_{\mathrm{mosaic}}$ estimates only decreased by $\approx 10\%$. This result indicates that CP-HMM's performance does not strongly rely on the strict adherence to the uniform sampling assumption.

3.3 Application to data from the gut microbiome

For each species, we applied our CP-HMM model to all pairs of strains where the fraction of identical blocks was $> 50\%$. We then refined this pool to focus on a subset of pairs where the clonal fraction was sufficiently high. This second step accounts for the fact that the clonal fraction is not always equivalent to the fraction of identical blocks, because the clonal region still allows mutations to occur at rate θ_c. To obtain the relevant subset of pairs, we used the scatterplots of clonal fraction vs clonal divergence in each species (Figs M & Q) to manually identify a critical divergence value d^*, above which the typical clonal fraction first dropped below $f_c^* \approx 75 - 85\%$. We then excluded all pairs of strains in which d or f_c exceeded these critical values. The d^* and f_c^* values for each species are illustrated in Figs M & Q and are listed in Table S6. The inferred recombination events and clonal divergence values the remaining pairs were used to create the scatter plots in Figures 2, 3, and P.

Due to the discreteness in the number of mutation and recombination events, these scatter plots often have

many overlapping points. To better visualize the underyling trend in data, we computed trend lines for each species using LOWESS (locally weighted scatterplot smoothing), a common local regression technique [25]. We chose an implementation provided by the Python package statsmodels [26], using the tri-cube weight function and $1/3$ as the fraction of the data used for each estimation.

To quantify the local spread around these trend lines, we developed a procedure for computing quantiles of weighted residuals. For any given x value, we found k nearest neighboring points and computed their weights given by the tri-cube weight function. After sorting the points by the their absolute residuals, we obtained the normalized cumulative weight as a function of the residual. Then, we inverted the function numerically to obtain the residual sizes corresponding to desired quantiles. In the scatterplots, we chose k to be $1/5$ of the total number of points and plotted the residuals corresponding to 66% quantile.

3.3.1 Evaluating the effects of different sample sizes

To evaluate how the sample size affects our CP-HMM results, we performed a series of downsampling experiments with the *A. putredinis* dataset (as low as 1/10 of the original size), and examined how the inferred transfer length and recombination strength ($T_{\mathrm{mrca}}/T_{\mathrm{mosaic}}$) varies with the number of metagenomic samples (Fig L). While the variance among bootstrap replicates increases slightly as the dataset size decreases, the inferred parameters remain very close to the values obtained for the full dataset.

However, while the raw size of the dataset seems to play a relatively minor role for this range of parameters, the uniformity of the sample (i.e. how well it represents the broader population of a given species) can be much more important. For example, skewed sampling can lead to technical issues: *Lachnospiraceae bacterium* has no fully recombined pairs (Fig C), so our CP-HMM algorithm cannot obtain a prior for the divergence of the transferred fragments, and has trouble identifying potential transfer events. Skewed sampling can also overrepresent a specific set of closely related strains, e.g. when sampling clinical outbreaks in bacterial pathogens [27–29] (see our analysis of *Salmonella enterica* in S1 Text 3.10). In these cases, the inferred recombination parameters can be biased by the recent evolutionary history of the clonal bloom, rather than the long-term dynamics of the broader species.

3.4 Correspondence between the number of transfers and the fraction of recombined genome

The crucial assumption that individual transfers do not overlap breaks down for more diverged pairs. These overlapping transfers are difficult to resolve into individual events, causing the number of transfers to be less representative of the overall rate of recombination. However, since overlapping transfers contain many more SNVs than the clonal regions, the non-clonal regions can still be reliably inferred by our model. This suggests that the *total fraction* of recombined regions can be used as a complementary metric to test if our previously observed patterns are robust to the effect of overlapping transfers.

Figure M shows an example of this metric for *B. vulgatus*, which complements the number of transfers shown in Fig 2. Similar to Fig 2C, we observed an overall trend of higher recombined fraction at longer divergence times. However, we once again observed pairs with large amounts of recombination at low divergence, and small amounts of recombination at high divergence. In addition, we saw that the vast majority of recombined regions are due to within-clade transfers, reflecting both the higher rates and longer transfer lengths we previously observed. This metric therefore confirmed the robustness of results reported in the main text. Similar results are observed when we apply this recombined fraction metric to other species with sufficient number of close pairs (Fig Q).

This alternative metric also allowed us to ask if recombined regions tend to consist of many transfers of typical lengths, or a smaller number of anomalously long transfers. To distinguish these scenarios,

we considered a simple model where the lengths of each transfer were drawn from the same exponential distribution, with a mean length given by our previous analysis (Fig 2D). We used this model to compute a 95% confidence interval for the fraction of recombined genome for different numbers of transferred fragments. By comparing the observed data with this null distribution, we identified many pairs of *B. vulgatus* strains where a small number of transfers covered an anomalously large fraction of the genome (Fig M).Further examination of these outlier pairs revealed extremely long recombination events that are hard to explain with this simple exponential model (Fig N). Long recombination events with similar length scales have previously been observed for several other bacterial species [15, 30, 31]. Intriguingly, some of the longest recombination events in our cohort appear to have multiple peaks in their associated divergence profiles, suggesting that they might actually be composed of several smaller transferred fragments. However, these non-contiguous transfers would still have to be strongly correlated with each other to cluster within the same localized regions, suggesting that they are best regarded as a single compound recombination event. We also observed long homozygous regions in a pair of *B. vulgatus* isolate genomes sampled from the same person (S1 Text 3.10, Fig O). This pattern is most likely generated by a few very long within-host transfer events, rather than correlated transfers that have compounded over multiple hosts. Understanding the mechanisms behind these long transfers is an interesting avenue for future work.

3.5 Comparing the recombination dynamics of *B. vulgatus* and *A. putredinis* to simulated data

***B. vulgatus* simulations.** To evaluate whether the recombination dynamics in *B. vulgatus* (Fig 2) can be explained by different clade sizes, counting noise, or other potential detection biases in the CP-HMM algorithm, we repeated our analysis using the simulated data from S1 Text 3.2 (Fig J). Although we observed some degree of variation in the number of transfers due to counting noise, the simulated relationship between the number of transfers and clonal divergence was mostly linear. In contrast, the observed *B. vulgatus* data show a marked excess of pairs that accumulated a large number of transfers at low clonal divergence. This shows that the heterogeneities observed in the data cannot be explained by counting noise alone. We also find that the simulated data show few differences between within- and between-clade transfers when their underlying recombination parameters are the same Fig 2. This indicates that the dramatic difference we observed in the *B. vulgatus* data cannot be explained by differences in the relative clade sizes, or a detection bias for transfers with different levels of divergence.

***A. putredinis* simulations.** To test how well a single underlying recombination rate can capture the broad variation in the realized transfer frequency in *A. putredinis* (Fig 3C), we again leveraged the computational model we developed for validating our HMM. We set the genome length and recombination length to be identical to those of *A. putredinis* and simulated three recombination rates $r/\mu = 5.2,\ 3.4,\ 2.1$, which roughly span the range of realized recombination frequency in data. Since we need to compare the size of variation at different divergence times with real data, it is crucial to control how the number of data points changes over divergence. For example, the apparently large variation at small clonal divergences in Fig 3C might be caused by a higher density of points. To account for this effect, we fitted an underlying distribution of divergence times to the distribution of observed clonal divergences in *A. putredinis*. We assumed that the true divergence time for each pair was a hidden random variable, which could take one of 20 discrete values covering the whole range of observed divergences. This hidden variable parameterized a corresponding Poisson random variable for the number of clonal mutations. The probability distribution for the hidden variables can be inferred from the observed distribution of clonal mutations in data, using the standard

expectation–maximization (EM) algorithm [24]. From this inferred distribution, we sampled the same total number of pairs as in *A. putredinis* and proceeded with the rest of the simulation steps.

The result of applying our CP-HMM algorithm to this set of simulations is shown in Fig K. We found that no single recombination rate can capture the full range of variation in *A. putredinis*. High recombination rates only reproduced the points in the upper-left corner (corresponding to pairs that accumulate a large number of transfers at short divergence times), while lacking the points in the lower-right corner. The low recombination rate simulation produces the opposite pattern. This indicates that a combination of low and high recombination rates are needed to explain the pattern observed in *A. putredinis*.

3.6 Estimating the divergence distribution of donor and recipient DNA sequences

One of the most common explanations for the reduced gene flow between diverged clades (e.g. Fig 2) is that the recombination rate is reduced for DNA segments with higher sequence divergence [32]. To test if this divergence dependent recombination rate plays a role for the gut bacteria we have analyzed here, we computed the distribution of donor-recipient divergence of all detected transfers within each of the species in Fig 3. For comparison, we obtained a null expectation of this distribution by simulating transfers with our collection of observed genomes, similar to our approach in S1 Text 3.2. If the effect of sequence divergence was significant, we would expect to observe a depletion of transfers with higher divergence (or an excess of transfers with low divergence) when compared to this null distribution.

In practice, the distribution of donor-recipient divergence can be influenced by other factors, such as the uneven distribution of transfers along the genome (e.g. recombination hotspots) or potential biases in the transfer detection step. To account for these effects, we simulated transfers with the genomic locations as the observed transfers, and applied the CP-HMM algorithm to the simulated data. The detailed simulation algorithm is as follows:

1. For each pair of closely related strains with observed transfers, we randomly chose one of the two strains as the focal genome, and the other as the reference genome.

2. For each observed transfer between this pair, we sampled a genome segment with the same start and end positions from a random strain of the same species. We then overwrote the corresponding regions of the focal genome with sampled segments. If the donor segment was completely identical to the recipient sequence (e.g. from another closely related strain), we repeated the sampling step.

3. The SNV differences between the focal genome and the reference genome now contain a mixture of SNVs in the clonal regions and SNVs imported from simulated transfers. We then applied our CP-HMM algorithm to the simulated SNV profile to obtain a corresponding set of inferred transfers. The divergence of each of these inferred transfers were then added to the null distribution.

4. We repeated steps 1-3 for 5 repetitions per close pair to ensure that the null distribution was sufficiently sampled.

5. We then repeated steps 1-4 for all close pairs across all species in Fig 3.

The comparisons between the observed distribution of donor-recipient divergences and the simulated null model are shown in Figs 4 & W. We found that the observed distribution closely tracks the simulated distribution in the majority of species, with only a few notable exceptions discussed in the main text. These results indicate that the typical donor-recipient divergence in our panel is not sufficiently high to suppress the recombination rate on its own, and that other incompatibilities are likely needed to explain the observed genetic isolation between clades in *B. vulgatus* and *B. finegoldii*.

3.6.1 Estimating the reduction in the realized recombination rate as a function of sequence divergence

To gain more intuition for the divergence distributions in Figs 4 & W, we fit these data to a simple model of a recombination barrier, where the realized recombination rate decays exponentially with the sequence divergence of the transferred segment,

$$P\{\text{transfer}|d\} \propto e^{-d\cdot\beta}\,. \tag{S16}$$

To estimate the β parameter from the distribution of observed transfers and simulated transfers in Fig W, we trained a standard logistic model to classify each transfer as *observed* or *simulated* based on its divergence,

$$P\{\text{obs}|d\} = \frac{e^{\beta_0 - d\cdot\beta_1}}{1 + e^{\beta_0 - d\cdot\beta_1}}\,, \tag{S17}$$

$$P\{\text{sim}|d\} = 1 - P\{\text{obs}|d\}\,. \tag{S18}$$

In the limit that the number of simulated transfers is much larger than the number of observed transfers, β_1 will approach the recombination barrier parameter, β, while β_0 fits the total fraction of observed transfers. We used the fitted values of β_1 for each species to predict the recombination rate reductions in Figure X.

3.7 Estimating the ratio between T_{mrca} and T_{mosaic}

One of the immediate consequences of being able to identify recombined regions is that we can estimate the ratio $T_{\text{mrca}}/T_{\text{mosaic}}$ while making fewer assumptions about the underlying population. In S1 Text 2, both the partial recombination model (Eq. S5) and the estimation formula (Eq. S13) rely on a simple neutral model to connect $T_{\text{mrca}}/T_{\text{mosaic}}$ with the underlying biological parameters, e.g. the recombination rate r, the recombination length l_r, and the divergence of transferred fragment d.

Empirically, the connection between $T_{\text{mrca}}/T_{\text{mosaic}}$ and underlying biological parameters is less straightforward. Part of the reason arises from our finding that in the presence of population structure, l_r, r and d are no longer a single set of numbers. Formally, we would need to sum over all these heterogeneous sources of recombination transfers to calculate T_{mosaic}.

However, in practice, our understanding of the extent of this heterogeneity is limited. For species like *B. vulgatus*, we could infer two sets of these parameters to account for within- and between-clade transfer separately, as demonstrated in the main text. But for the majority of species with complex population structures, such as *A. putredinis*, it is much harder to even enumerate the types of transfers we need to consider separately. Potentially, there could be a continuum distribution of these recombination parameters, as argued in [13].

Nevertheless, the $T_{\text{mrca}}/T_{\text{mosaic}}$ ratio can still be studied empirically without explicit inference of the above biological parameters. The idea is that we can estimate the actual time it takes for a strain to be fully covered by recombination transfers, irrespective of the underlying heterogeneity. This time, T_{mosaic}, will vary for different strains and could be highly random, but on average it should reflect the characteristic level of recombination of a given species.

Concretely, for each closely related pair, our CP-HMM algorithm can identify the total fraction of recombined genome, f_r, as well as the clonal divergence, d_c. If we assume that both f_r and d_c increase linearly in divergence time T, then

$$\frac{f_r(T)}{d_c(T)} = \frac{f_r(T_{\text{mosaic}})}{d_c(T_{\text{mosaic}})} = \frac{1}{d_c(T_{\text{mosaic}})}\,, \tag{S19}$$

where we have used the fact that $f_r(T_{\rm mosaic}) = 1$. This implies that

$$\frac{T_{\rm mrca}}{T_{\rm mosaic}} = \frac{d_c(T_{\rm mrca})}{d_c(T_{\rm mosaic})} = \frac{f_r(T)\overline{d}}{d_c(T)}\,, \tag{S20}$$

where $\overline{d}$ is the average pairwise divergence in the population. This allows us to estimate $T_{\rm mrca}/T_{\rm mosaic}$ for each pair of closely related strains.

In Fig R, we plot the distribution of these $T_{\rm mrca}/T_{\rm mosaic}$ estimates for all close pairs across a panel of different species. These estimates can also be viewed as a distribution of "r/m" values. We see that these estimates can vary substantially within certain species; this spread will reflect a mixture of the Poisson noise in the accumulation of mutation and recombination events (Fig 1E), as well as potential variations in the underlying recombination rate (Fig K) or other deviations from the simple neutral model above. However, this broad distribution within species does not overshadow the systematic differences between species. We can obtain an average $T_{\rm mrca}/T_{\rm mosaic}$ ratio for each species by calculating an average $d_c(T_{\rm mosaic})$:

$$\hat{d}_c(T_{\rm mosaic}) = \left(\frac{1}{n}\sum_i^n \frac{f_r(T_i)}{d_c(T_i)}\right)^{-1}\,, \tag{S21}$$

and substituting this value into Eq. (S20):

$$\frac{T_{\rm mrca}}{T_{\rm mosaic}} = \left(\frac{1}{n}\sum_i^n \frac{f_r(T_i)\overline{d}}{d_c(T_i)}\right)\,. \tag{S22}$$

The resulting estimates for each species are shown in Fig R, which broadly agree with the coarse-grained estimates in Fig 1F (Fig S).

3.8 Estimating the number of recombination events in a single gut microbiome

We can gain additional intuition for the recombination rate estimates in Fig 3 by extrapolating them to the scale of an individual gut microbiome. For example, one important quantity is the total number of recombinants that are produced within a given species population each day. This quantity can be estimated from the product

$$n_{r,\rm tot} \sim (N \cdot f) \cdot \left(\frac{r}{\mu} \cdot \mu \cdot L\right) \cdot \Delta t\,, \tag{S23}$$

where N is the total number of bacterial cells in the gut microbiome, f is the relative abundance of the species, μ is the per site mutation rate, r is the corresponding recombination rate, L is the total length of the genome, and Δt is the number of generations that take place per day. If we assume that successful recombination events accumulate largely neutrally (or via neutral hitchhiking), then the relevant r/μ values can be estimated from the apparent rates of accumulation in Fig 3D; these range from 0.01−1, with most species clustering near $r/\mu \sim 0.1$. Using estimates of the remaining parameters from the existing literature [$N \sim 10^{13}$−10^{14} [33], $\mu \sim 10^{-10}$−10^{-9} [34], $L \sim 10^{6.5}$ [35], $\Delta t \sim 1$−10 [36, 37]], we expect that a moderately abundant species ($f \sim 3$–30%) will produce anywhere from 10^6 to 10^{12} recombinant offspring each day.

To convert this estimate to a per-site rate, we note that each site will have a probability ℓ_r/L of being covered by a given recombination event, where ℓ_r is the typical length of a transferred fragment. Using the typical lengths inferred from Fig 3E ($\ell_r \sim 10^{3.5} - 10^{4.5}$), we conclude that each site in the genome will be involved in 10^3–10^{10} unique transfers each day.

To convert these population-level estimates to a per-lineage rate (e.g. to estimate the total number of recombination events that are expected within a single isolate genome), we need to drop the factor of Nf in Eq. (S23). This implies that a typical genome will accumulate homologous recombination events at a total rate

$$\lambda_r \sim \left(\frac{r}{\mu} \cdot \mu \cdot L\right) \cdot \Delta t \sim 10^{-5.5}\text{–}10^{-1.5}\,\text{days}^{-1}\,, \tag{S24}$$

with most genomes clustered near the middle of this range (λ_r^{-1}~3000 days, or ~ 10 years).

All of these extrapolations assume that recombination events accumulate largely neutrally (or via neutral hitchhiking) when averaged over multiple host colonization cycles. If this assumption is violated, than the apparent rates of accumulation in Fig 3D will differ from the true value of r/μ by an additional factor of $\gamma = N\overline{p}_{\text{est}}$, which represents the average "establishment probability" of a newly acquired fragment. If the between-host patterns are driven by widespread positive selection ($N\overline{p}_{\text{est}} \gg 1$), then the true values of r/μ will be much smaller than the apparent values in Fig 3D. Conversely, if most recombination events are negatively selected ($N\overline{p}_{\text{est}} \gg 1$), then the true values of r/μ will tend to be larger than the apparent values in Fig 3D. Understanding the relative contributions of these selective forces is an interesting topic for future work.

3.9 Deduplicating detected recombination events

One limitation of our pairwise detection method is that a single recombination event could be detected in multiple pairs. As a concrete example, consider three closely related strains, A, B and C, that have diverged very recently $T_{ABC} \ll T_{\text{mosaic}}$. Suppose A and B have diverged more recently from a common ancestor at $T_{AB} < T_{ABC}$ and this common ancestor acquired a recombined fragment between T_{AB} and T_{ABC}. This recombination event would then show up *twice* when we compared the genomes of pairs AC or BC. On the other hand, if both A and B acquired different recombined fragments after they diverged, we would also see two events when we compared AC and BC.

For most of our pairwise analyses, we do not need to distinguish these two scenarios as long as we average across pairs. However, if we are interested in non-pairwise statistics, such as how frequently one region is transferred among many strains, we need to identify unique recombination events to minimize the impact of sampling bias. For example, if the common ancestor of A and B in the example above happened to have many more descendants in the sampled dataset, the same recombination event would be vastly over-represented if we naively retained all detected events.

In some cases, it is possible to infer the clonal relationships between strains using regions not affected by recombination and leverage the genealogical tree to identify unique events along the branches (e.g. Gubbins [22] or RecHMM [38]). Here, we adopt a simpler but approximate approach to deduplicate detected events using their genomic locations and sequence identity. The basic idea is that genomes that share the same transfer event by descent will show up in different pairs at approximately the same start and end locations with approximately the same sequence identity. This approach is conservative in that it will merge separate transfer events of the same region of the genome with the same donated haplotype. However, we assume that it is unlikely for two independent transfer events to have nearly identical start and end locations, and thus we expect these cases will have a small impact on the overall patterns of recombination that we detect.

More specifically, we first group together all transfers by their start and end locations, treating them as candidate duplicates if their start and end locations are in the same genome bin of 1000 sites. Next, we examine all the imported SNVs within the transfer region, and compute the fraction of SNVs that are identical between detected events. We consider two events to be duplicates if they have similar start and end locations (defined by the first step) and share > 98% of imported SNVs. This criterion is stricter than simply requiring

a high sequence identity between the transferred fragments, since the sequences are always only a few percent diverged within a particular species.

We applied this deduplication procedure to all the detected events in Fig 3 and found a total of 57,179 approximate unique events. We have included the uniqueness of each event in Table S3 and used only unique events in the analysis of transfer lengths (Fig 2D and Fig 3E). These unique events allowed us to study whether some genomic locations are biased toward more frequent recombination events (Fig JJ). We found that while recombination events can be detected throughout the genome, the landscape of detected transfers varies significantly along the genome, similar to the sharing landscape Fig 6. Additional examination suggests that at least some of the peaks in the landscape coincide with putative recombination hot spots (Fig LLC, [39, 40]). Interestingly however, when aggregated across the genome, we observed only weak correlations between the transfer landscape and the sharing landscapes across species (Fig JJC). This lack of correlation suggests that these two landscapes can provide complementary information about the complex interplay between recombination and natural selection.

3.10 Application to isolate genomes

We obtained alignments of high quality, isolate genomes of nine bacterial species from the Unified Human Gastrointestinal Genome (UHGG) collection [8] (Table S7), including five gut commensals in our metagenomic panel and four additional pathogens. We applied our CP-HMM model in the same fashion as our analysis of quasi-phased metagenomic genomes and repeated a number of analyses on the inferred transfer events analogous to Figures 1, 2, and 3.

Many of our results are reproduced using isolate genomes. We showed that the patterns of recombination barrier in B. vulgatus (Fig 2) is quantitatively reproduced in the isolate analysis (Fig OAB) – among 40 close pairs, we detected 239 within-clade transfers and 52 between-clade transfers, consistent with the 5-fold reduction observed in the main text analysis. Other quantitative validations include the rate of accumulation of recombination events (Fig Q and Fig OF-I) and the median transfer lengths (Fig OK). We also reproduced qualitative results such as the significant variation in the sharing landscape (Fig 6, Fig OJ); and interesting examples of a putative within-host recombination event and a large recombination event (Fig OC), suggesting the generality of the events in Fig 5 and Fig N.

3.10.1 Comparisons with previous studies of bacterial pathogens.

Our analysis of the pathogen species in Fig LL allows for a more direct comparison of our methods with the results of previous studies. For example, by analyzing the locations of the detected transfers in *Campylobacter jejuni* (Fig LLBC), the most deeply sampled pathogen species in our dataset, we were able to reproduce the exact locations of recombination hot spots previously identified by the orderedPainting algorithm in Ref. 39. We also used the data from Ref. 41 to obtain a pairwise recombination fraction vs clonal divergence plot (analogous to Fig Q), and found these data to be broadly consistent with our CP-HMM results (Fig LLB).

Comparing species-level summaries of recombination parameters like r/m is more challenging, however. We compiled r/m estimates (or close equivalents) from two previous studies [16, 42] for the subset of pathogen species we analyzed in Fig LL (Table A). These data show that the estimated values of r/m can differ by orders of magnitude in different studies — even the rank ordering of r/m for different species can vary widely. For example, Ref. 16 reported an r/m value for *Helicobacter pylori* that is ¿40 times larger than *Klebsiella pneumoniae*, but Ref. 42 estimated this value to be ¿20 times smaller than the *K. pneumoniae* rate. Similarly, our CP-HMM analysis suggests that the r/m value for *C. jejuni* is ¿30 times greater than that of *Salmonella enterica*, while Ref. 16 reported it to be ∼13 times smaller.

Table A. Comparison of inferred recombination rates for several pathogen species.

Species name	r/m [16]	$\rho = \phi_{pool}\overline{f}$ [42]	T_{mrca}/T_{mosaic}
Vibrio parahaemolyticus	39.8	-	18.3
Salmonella enterica	30.2	-	0.9
Helicobacter pylori	13.6	148	-
Campylobacter jejuni	2.2	200	39.5
Klebsiella pneumoniae	0.3	3248	23.1
Staphylococcus aureus	0.1	23.1	-

Some of these discrepancies are expected due to the heterogeneity in recombination rate within species, as demonstrated both by our current results as well as a large number of previous studies [13, 43–46]). For example, in the case of *S. enterica*, our pairwise estimates reveal that some of the discrepancies might be caused by the fact that a small number of pairs with low clonal divergence acquired a large amount of transferred DNA, while a large number of pairs showed no evidence of recombination (Fig LLG, H). This heterogeneity is consistent with previous findings that the r/m of *S. enterica* can differ by 10-fold between sub-lineages [46]. Summarizing such wide variation with a single value of r/m is inherently difficult, and will sensitively depend on the composition of the sample and the averaging scheme employed. In more sophisticated inference methods, this average scheme is not defined explicitly, but emerges through a complex process of likelihood optimization of an underlying parametric model. A potential benefit of semi-parametric approaches like CP-HMM (and related methods [41, 47]) is that they decouple the detection of individual recombination events from the averaging step at the end. By directly examining scatter plots like Fig 2 and Fig 3, it is possible to gain more insight into how different features of the data contribute to a given r/m estimate, and to more easily compare results across studies (e.g. Fig LLB).

4 Identifying within-host recombination among co-colonizing strains

The within-host diversity of a given species can vary widely from host-to-host, and also within the same host over time [1]. In the previous two sections, we focused on the simplest subset of samples, where a single strain is present at high frequencies. The site frequency spectra (SFS) of these "quasi-phaseable" (QP) samples contain very few alleles at intermediate frequencies (top left of Fig A), so that the haplotypes of the dominant strains can be inferred with a high degree of confidence [1].

In this section, we revisit a subset of the remaining non-QP samples – which by definition contain multiple co-colonizing strains – to identify signatures of recent or ongoing recombination within hosts. Frequently, a non-QP sample features a single intermediate peak in the SFS, indicating the coexistence of two diverged strains. The basic signature of recombination between these strains will look like the inverse of the close-pair analysis above: rather than looking for regions of high divergence among closely related pairs, we search for regions of low divergence (i.e. identical DNA sequence) within a normally diverged background ($d{\sim}1\%$). These regions of shared DNA sequence will manifest as a local depletion of genetic diversity in the larger metagenomic sample (Fig 5).

4.1 Sample selection

In the idealized scenario of two coexisting clonal populations, all fixed differences between the two strains should be found at a certain intermediate frequency corresponding to the relative frequency of the two strains. Thus, in principle, we should be able to readily identify all fixed differences between two strains in these

dual-colonized samples.

However, as shown on the left of Fig A, the observed SNVs usually form a broader distribution around the peak frequency. Sampling noise from finite sequencing coverage is likely a major contributor to this spread, but the large widths observed in some high coverage samples suggests additional processes might be involved. In either case, when the shoulder of the intermediate frequency peak merges with the main peak at 100% frequency, we can no longer reliably detect all fixed differences between the two co-colonizing strains. This could lead to spurious signals of recombination where none truly exist.

To avoid this difficulty, we restricted our attention to a particularly simple subset of dual-colonized samples which were identified by two criteria: (i) the SFS should have a single, pronounced peak at intermediate frequency, and (ii) the intermediate peak should be clearly separated from the main peak. We can then use this clear separation to reliably identify fixed differences (or the lack of fixed differences) between the pair of co-colonizing strains. To implement these criteria programmatically, we first smoothed the SFS with a Savitzky-Golay filter and detected peaks using the SciPy signal processing library [14]. We next quantified the quality of separation between peaks by computing the trough-to-peak ratio. Only samples with a ratio smaller than 0.2 were deemed to be simple enough for further analysis. The location of the trough was then used as a cutoff to classify sites as fixed differences between two strains (or not). Examples of simple and complex co-colonized samples are shown in Fig A.

We note, however, that this frequency-based classification will only for sites that are present in both of the co-colonizing strains. If a site is present in only one strain (e.g. due to a gene loss event), then a mutation can appear to be fixed within the remaining read alignments without actually being fixed in the population. To reliably filter such gene loss events, we restricted our attention to an even smaller subset of high quality samples where the noise in gene copy numbers was also minimal. Fig Y illustrates an example of this additional processing step for an example *B. vulgatus* population. We first computed the median read depth for each of the core genes ($\overline{D}_i$, where i is the gene index), and then calculated a moving average of 500 genes to estimate the large-scale depth variation due to bacterial growth dynamics [48] (Fig Y top). Next, we used this average variation ($\langle \overline{D} \rangle_i$) to compute the relative copy number of each gene, defined as $c = \overline{D}_i / \langle \overline{D} \rangle_i$. If a gene is present in both strains, its copy number should remain close to one; if the gene is present in only one of the co-colonizing strains, its copy number should be close to the corresponding strain frequency (as inferred from the peak of the SFS, Fig Y middle). We computed the standard deviation of the copy number across all genes (σ_c) to quantify the expected variation due to sequencing noise, and we used this estimate to compute a corresponding Z-score for each gene,

$$Z = \frac{c-1}{\sigma_c}\,, \tag{S25}$$

which quantifies the magnitude of the deviation from unit copy number. Genes with $|Z| > 2$ are filtered from our downstream analysis. In cases where the sequencing noise is large enough that a gene deletion event cannot possibly be distinguished from noise ($1 - f_{\text{peak}} < 2\sigma_c$), we discard the entire sample from our downstream analysis. This procedure yields a set of co-colonized samples where we can confidently identify the set of genes that are present in both strains. We then classified the fixed differences and shared sites between the strains using the same frequency thresholds defined above.

Applying this procedure to our cohort yielded a total of 8 species (from 4 bacterial families) with ≥ 5 high-quality dual-colonization examples (Table S4). The two species with the largest sample sizes (*B. vulgatus* and *E. rectale*) are highlighted in Fig 5.

4.2 Identifying shared genomic regions among co-colonizing strains

The fixation of a successful recombination event will leave an extended run of zero fixed differences in the genomes of co-colonizing strains. To look for enrichment of such signals, we computed the lengths of all continuous stretches of 4D sites in the core genome that did not possess any fixed differences (a *homozygous run*). Filtered genes or other low coverage sites were omitted from this calculation. It is worth noting that any underlying recombination event could be longer than the measured run length if the transferred fragment also contained some accessory genes.

To interpret these measured run lengths, we first compared them to a null model in which the SNVs were randomly distributed across the high coverage sites in the genome. Let d be the pairwise divergence between a pair of strains. Then, the distance between two consecutive SNVs (x) follows a geometric distribution with mean $(1-d)/d \approx 1/d$, which can be approximated by a continuous exponential distribution when the distance is large. Thus, the probability of observing a homozygous run longer than ℓ under this null model is given by

$$\Pr[x > \ell] = e^{-\ell d}, \tag{S26}$$

which was used to plot the null distributions in Figs. Z and CC. This comparison revealed that co-colonizing strains can frequently share long homozygous runs that cannot be explained by the stochastic distribution of mutations along the genome ($\ell d > 25$, or equivalently $P \lesssim 10^{-11}$). These shared genomic segments constitute potential candidates for within-host recombination events.

4.3 Distinguishing within-host recombination from pre-existing sharing

To determine whether the shared genomic segments observed in co-colonizing strains were caused by previous within-host recombination events, we compared them to the baseline rates of sharing observed in random pairs of strains from unrelated hosts. We found that these random pairs can sometimes share segments that are just as long as those observed in co-colonizing strains (Fig Z; see S1 Text 5.1 for a theoretical explanation). This suggests that it will be difficult to determine whether any *particular* shared segment was transferred before or after colonization. We therefore sought to quantify the global signatures of within-host recombination by examining the statistical enrichment of sharing among co-colonizing strains.

To perform this comparison, we took advantage of our large collection of QP strains and generated an empirical null distribution of runs of shared sequence between unrelated strains. We kept only one sample per host/household to approximate a random set of strains from the broader population. Since we are interested in recent recombination events between these strains, we want to minimize runs of shared sequence that arise through clonal inheritance of the entire genome (Fig 1A). We therefore only analyzed "typically diverged" pairs with $< 10\%$ identical blocks in Fig 1B. These pairs share a common ancestor long enough ago that imported fragments should have overwritten most of the clonal fraction, and any long sharing regions should be mostly due to subsequent recombination events between the ancestors of the pair. We then sampled a random subset of 5,000 between-host pairs for each species and computed the homozygous run lengths for each pair as described above; in cases where a species had fewer than 5,000 qualified pairs, we used all available pairs. There were two species that required additional consideration:

1. Anticipating the potential impact of the strong clade structure in *B. vulgatus*, we analyzed between-clade pairs separately from within-clade comparisons. To do so, we first clustered all the strains into two clades as described in S1 Text 2, and obtained the resulting within- vs between-clade strain combinations. Out of the 372 strains of *B. vulgatus* in our cohort, we identified 12,935 between-clade pairs and 16,711 within-clade pairs with "typical" levels of divergence.

2. *Eubacterium rectale* has a geographically structured population, which suggests that co-colonizing strains are sampled from a different distribution than the worldwide population. To account for this issue, we also need to control for geographical structure when selecting the between-host pairs for our null distribution. In our cohort, there are 15 co-colonized samples collected in the United States, and 13 samples collected in the United Kingdom. To reflect this geographical distribution, we sampled a subset of pairs from the US pool of *E. rectale* strains and another subset of pairs from the UK pool. We combined these subsets in a 15:13 ratio, yielding a total of 3000 strain pairs that reflect the geographical distribution of our co-colonized samples.

To compare these empirical null distributions with the observed within-host data, we used a test statistic based on the length of the longest homozygous region for each pair. The reverse cumulative distributions of these "max run" statistics for *B. vulgatus* and *E. rectale* are shown in Fig 5D in the main text, and analogous distributions for the other species are shown in Fig BB. We calculated P-values for the deviation between within-host and between-host distributions using a one-sided Kolmogorov-Smirnov test using the `ks_2samp` function in the SciPy package [14].

We also analyzed a complementary metric that sums all the runs longer than a given threshold ℓ^*. This metric is more suitable to detect the enrichment of *multiple* transfer events between a given pair of strains. We choose the length threshold ℓ^* such that $\ell^* \cdot \overline{d} = 30$, where $\overline{d}$ is the average pairwise divergence between the typically diverged pairs of that species (e.g. equivalent to $\sim$ 20kb in the case of *B. vulgatus*). This ensures that the sum is dominated by runs that likely reflect recent transfer events, rather than the random spacing of SNVs along the genome. Within- vs between-host comparisons of this new metric yielded results that were similar to the "max run" statistic above, with only *E. rectale* showing an enrichment of long runs in co-colonizing strains Fig AA.

5 Identifying signatures of selection from the global distribution of recent transfers

5.1 Neutral expectation for the probability of observing long shared fragments

Using the results in Ref. 49, one can derive an approximate formula for the expected number of long shared fragments in a simple neutral model with a constant population size. Consider a genome comparison between a random pair of strains. Let $H(l)$ be the probability that a randomly chosen site will be a SNV and followed on the right by l identical sites. In general, $H(l)$ can not be computed analytically because we need to consider all possible recombination histories within this shared segment. However, since recombination typically brings in SNVs that break a run of identical sites, we can expand $H(l)$ in terms of the number of recombination break points it contains, under the assumption that contributions from shared segments spanning multiple ancestral recombination events become increasingly small [49]. For simplicity, here we only consider the contribution from the lowest order term, in which the shared segment contains zero ancestral recombination events (Ref. 49 computed this quantity for up to two crossover recombination events). In this

case, the $H(l)$ function can be approximated by

$$H(l) \approx \int_0^\infty (1 - e^{-2\mu T}) \cdot (e^{-2(\mu+r)T})^l \cdot \frac{1}{T_c} e^{-T/T_c} dT \, , \tag{S27}$$

$$= \frac{2\mu T_c}{(2(\mu + r)T_c l + 1)^2} \, , \tag{S28}$$

$$= \frac{\overline{d}}{((r/\mu + 1)l\overline{d} + 1)^2} \, , \tag{S29}$$

where μ and r are the per site, per generation mutation and recombination rates, T_c is the average coalescence time in the population, and $\overline{d} = 2\mu T_c$ is the average pairwise divergence. Since we are interested in long shared fragments ($l \cdot \overline{d} \gg 1$), we can further approximate the above formula as

$$H(l) \approx \frac{1}{\overline{d}l^2(r/\mu + 1)^2} \, . \tag{S30}$$

Using this formula, the expected number of shared fragments with length exactly l in a genome of length L is given by

$$n_L(l) = (L - l - 1)(H(l) - H(l + 1)) \approx \frac{2L}{\overline{d}l^3(r/\mu + 1)^2} \, . \tag{S31}$$

Finally, the total probability of observing a shared fragment longer than l^* is given by

$$\Pr[l > l^*] \propto \sum_{l > l^*} n_L(l) \approx \int_{l^*}^\infty \frac{2L}{\overline{d}(r/\mu + 1)^2} l^{-3} dl = \frac{L}{\overline{d}l^2(r/\mu + 1)^2} \, . \tag{S32}$$

Note that this distribution has a heavier tail that the exponential distribution of run lengths expected from random mutations alone (Eq. S26). This shows that it is not surprising to observe long shared fragments between random pairs of strains in neutral, recombining populations. We verified this intuition using computer simulations (Fig CC).

5.2 Quantifying parallelism in recent transfers

While neutral models like Eq. (S32) predict that a given pair of genomes can share several long segments, they place strong constraints on the probability that a particular fragment will be shared across multiple independent pairs (see S1 Text 5.3 below). We quantified this signature of parallelism using the haplotype sharing metric illustrated in Fig 6A. For each 4D site in the core genome, we recorded the fraction of pairs in which that site was contained within a run of length $\gtrsim \ell^*$. We call this normalized quantity the "sharing probability." As above, we chose the threshold such that $\ell^* \cdot \overline{d} = 15$, in order to distinguish likely recombination events from random fluctuations in SNV spacing.

The resulting "sharing landscapes" in Fig 6 were calculated using the same set of between-host pairs that were previously used as the null distribution in Figs. 5D and BB (S1 Text 4.3). In particular, we (1) used only typically diverged pairs ($< 10\%$ identical blocks), (2) analyzed between- and within-clade pairs of *B. vulgatus* separately, and (3) controlled the geographical structure of *E. rectale* as before.

Compared to neutral expectations (Fig B), a greater fraction of closely related pairs is observed in some species (e.g. *Bacteroides caccae*, Fig C), potentially reflecting certain sampling biases in the dataset. To minimize the impact of the over-representation of these closely related strains, we clustered strains with fraction of identical blocks $> 95\%$ and keep only one representative from each cluster.

We used a similar approach to quantify parallel transfers in pairs of co-colonizing strains as well (Fig 6C), using the homozygous runs identified in S1 Text 4.2. For hosts with multiple time points (e.g. the within-host sweep example in Fig 5C), we only included the comparison from the last time point in order to avoid counting the same pair twice.

5.2.1 Connections to selection tests based on haplotype homozygosity.

Our sharing probability metric is closely analogous to statistics based on haplotype homozygosity [50, 51], with a few key differences. For bacteria, two random genomes can share a haplotype either through clonal inheritance or a recent recombination event containing the haplotype. Haplotype homozygosity (and its close relatives) measures the total probability of these two modes of sharing. In contrast, our sharing probability statistic focuses on the second mode of sharing by only examining pairs with minimal clonal regions (i.e. typically diverged pairs as in Fig 5B, rather than close pairs as in Fig 2B). When none of the genome pairs share clonal regions, we expect the sharing probability to be equivalent to haplotype homozygosity (e.g. Fig 6A), where the length threshold above, ℓ^*, serves the role of the window size in identifying the local haplotype spectra. However, if some of the pairs share large fractions of clonal ancestry (¿10%), then the sharing probability will generally differ from the local haplotype spectrum. We therefore expect our pairwise sharing probability statistic to be better suited for detecting recent transfers than haplotype homozygosity alone.

To better illustrate the connection with existing statistics, we compared the within-clade sharing landscape of *B. vulgatus* with genome-wide scans of haplotype homozygosity (Fig GG). As expected, the variations of haplotype homozygosity closely follow the sharing landscape along the genome, and all statistics are strongly elevated at regions with a single, abundant haplotype (Fig GGD,E). However, the sharing landscape also contains peaks at regions where multiple haplotypes are circulating at lower frequencies (Fig GGF), which are harder to detect by simple versions of haplotype homozygosity statistics (Fig GGB). It is worth noting that by itself, the sharing probability statistic does not distinguish elevated values caused by one haplotype or multiple. Explicit analysis of haplotypes, or alternatively, statistics that account for the contribution of the most dominant haplotype (Fig GGB), can help distinguish these scenarios.

5.3 Comparison to simulated data from simple neutral models

To calculate the expected sharing landscapes of a neutral population, we performed simulations using the simulator FastSimBac [52]. FastSimBac simulates a recombining population and is capable of generating haplotype sequences for a large number of genomes. This allows us to compute statistics involving multiple strains (e.g. how frequent a region is shared by multiple pairs), which cannot be obtained by our simpler pairwise simulations in S1 Text 3.2. The simulations in Fig 6B were run with parameters that were chosen to match the observed data from *B. vulgatus*. We set the genome length to be 2.8×10^5 (which corresponds to the synonymous core genome length of *B. vulgatus*) and simulated a sample size of 200 genomes. We set the scaled mutation rate θ, to be equal to the within-clade diversity of *B. vulgatus* ($\bar{d}_{within} \approx 0.008$). We chose the recombination length to be approximately equal to the average recombination length inferred for *B. vulgatus* in Fig 2 ($\lambda = 2000$), and scanned over a range of scaled recombination rates ($\rho/\theta \in \{0, 0.1, 0.2, 0.5, 1, 2, 4\}$). Each parameter was simulated for 100 replicate populations with a constant population size. Each of these simulation runs generated a sample of 200 genomes, which we analyzed in exactly the same way as the between-host analysis above. In particular, we only used typically diverged pairs (i.e. those with an identical fraction $< 10\%$) when calculating the resulting sharing landscapes. This filter is important because some parameter combinations tended to produce a much larger fraction of pairs that inherited large clonal regions.

We computed the sharing landscape of these simulated populations for a range of threshold lengths ($\ell^* \cdot \overline{d} = 10, 12, \ldots 34$). For each sharing landscape, we computed the median sharing probability as well as the coefficient of variation (CV) in sharing probability (Fig HH). We found that adjusting the threshold length has roughly equivalent effects as adjusting ρ/θ: increasing both parameters would decrease the median sharing probability while leaving the CV almost unchanged. Since higher values of ρ/θ were more computationally expensive to simulate, we used this empirical scaling relation to cover a wider range of median sharing probabilities than would be feasible to simulate directly.

To compare the sharing distribution in simulations with that of *B. vulgatus* (Fig 6C), we found the combination of effective ℓ^* and ρ/θ values that matched the median sharing probability of the observed data (Fig HHA). The resulting sharing landscape were shown in Fig 6B.

To test the effects of a locally elevated recombination rate, we used the `-R` option of FastSimBac to specify regions of modified recombination rates. Figure II shows that elevated recombination rates actually *lower* the local sharing probability, which is consistent with the intuition that faster recombination generates more unique haplotypes. Conversely, Fig II shows that lower recombination rates slightly increase the sharing probability, but the overall levels still remain much lower than the "hotspots" observed in *B. vulgatus*. Curiously, we also observe a non-monotonic increase of the local sharing probability as we lower the local recombination rates: a small decrease in recombination rate initially increases the local sharing probability, but larger decreases eventually eliminate this pattern. Understanding the origins of this non-monotonic behavior would be an interesting topic for future work.

5.4 Quantifying the differences in the sharing landscapes of co-colonizing strains

The within-host sharing landscape of *E. rectale* deviates from its between-host counterpart at multiple locations along the genome (Fig 6C). To quantify the significance of this trend, we compared the observed data to a null model in which the within- and between-host labels were randomly permuted across pairs. For each of these bootstrapped datasets, we computed the sharing landscape as above and recorded the *excess sharing* (defined as the difference between the sharing probabilities of within vs between host pairs) at each location along the core genome. By comparing the observed data with the null distribution of $n = 5000$ bootstrapped datasets, we obtained an array of (uncorrected) P-values for each location.

To adjust these P-values for multiple comparisons, we performed a second level of bootstrapping. For each of the permuted datasets, we computed an analogous array of P-values using the other $n - 1$ permutations as the null distribution. To obtain a global metric of the deviation from this null model, we computed the total number of sites with $P < 0.01$ in both the observed and simulated datasets. We found that the total number of sites with excess sharing was significantly longer in the observed data than in any of the bootstrapped datasets ($P < 0.001$).

This second level of bootstrapping also allowed us to identify local deviations from the null model. To do so, we took the observed and simulated P-value arrays from our analysis above and created smoothed versions using a sliding window of 1000 sites. We then recorded the *minimum* smoothed P-value across the genome for each simulated dataset. We selected the 5th percentile of this distribution as a threshold for assessing the smoothed P-values observed in the data: a smoothed P-value below this threshold is unlikely to have occurred anywhere in the genome under the null model ($P < 0.05$). This local metric enabled us to identify many individual regions along the genome where the within-host sharing probabilities were significantly larger than the corresponding between host distribution ($P < 0.05$; Fig KK). Some enriched regions coincided with regions of moderate between-host sharing (e.g. near location 150,000), while others were entirely new (e.g. near location 90,000).

To further validate our approach, we repeated the above process using an additional permutated dataset

that was not in the n simulated datasets above. As expected, this negative control showed no region in the genome that had significantly more excess sharing than the simulated datasets (Fig KK). This result confirms that our two-level bootstrapping approach provides an adequate correction for multiple-hypothesis testing.

5.5 Enrichment analysis of functional classes of genes

To test if certain classes of genes were enriched for frequent sharing across hosts, we compared the observed data to a null model in which the gene annotations were randomly permuted across genes. To preserve potential co-occurrence of gene clusters, we permuted annotations in groups of 10 consecutive genes. For each permutation, we recorded the genes in regions of frequent sharing (sharing probability > 10%), and computed the number of genes in the functional class of interest. We repeated this procedure for 10^5 bootstrapped datasets, and defined a corresponding P-value based on the fraction of bootstrapped datasets that exceeded the observed value.

We applied this test to two candidate functional classes, glycosyltransferases and ribosomal proteins, which frequently appeared in the sharing hotspots of *B. vulgatus*. We found that for *B. vulgatus*, the glycosyltransferases were enriched for within-clade sharing ($p < 10^{-2}$) while the ribosomal proteins were enriched for between-clade sharing ($p \approx 10^{-3}$). The spatial distribution of genes belonging to these two classes coincide with some of the most prominent sharing hotspots in *B. vulgatus*, suggesting that selection on these genes could play a major role in shaping the overall patterns of the sharing landscape. This observation is consistent with previous work in *B. fragilis*, which found that glycosyltransferases were frequent targets of selection within individual hosts [53]. Interestingly, ribosomal proteins have previously been shown to be associated with recombination "cold spots" in a number of other bacterial species [40]. This observation could be consistent with the scenario of recent local sweeps, which leads to a shorter coalescence time and less time to recombine. Further analysis is needed to establish the differences between the sharing hotspots examined here and the recombination "hot/cold regions" inferred by existing chromosome painting algorithms [39, 54]. Combining the information encoded in these different statistics could be an interesting avenue for future work.

References

1. Garud NR, Good BH, Hallatschek O, Pollard KS. Evolutionary dynamics of bacteria in the gut microbiome within and across hosts. PLOS Biology. 2019;17(1):e3000102. doi:10.1371/journal.pbio.3000102.

2. Consortium HMP. A framework for human microbiome research. Nature. 2012;486(74027402):215–221. doi:10.1038/nature11209.

3. Lloyd-Price J, Mahurkar A, Rahnavard G, Crabtree J, Orvis J, Hall AB, et al. Strains, functions and dynamics in the expanded Human Microbiome Project. Nature. 2017;550(7674):61–66.

4. Xie H, Guo R, Zhong H, Feng Q, Lan Z, Qin B, et al. Shotgun metagenomics of 250 adult twins reveals genetic and environmental impacts on the gut microbiome. Cell systems. 2016;3(6):572–584.

5. Qin J, Li Y, Cai Z, Li S, Zhu J, Zhang F, et al. A metagenome-wide association study of gut microbiota in type 2 diabetes. Nature. 2012;490(7418):55–60.

6. Korpela K, Costea P, Coelho LP, Kandels-Lewis S, Willemsen G, Boomsma DI, et al. Selective maternal seeding and environment shape the human gut microbiome. Genome research. 2018;28(4):561–568.

7. Davis JJ, Wattam AR, Aziz RK, Brettin T, Butler R, Butler RM, et al. The PATRIC Bioinformatics Resource Center: expanding data and analysis capabilities. Nucleic Acids Research. 2020;48(D1):D606–D612. doi:10.1093/nar/gkz943.

8. Almeida A, Nayfach S, Boland M, Strozzi F, Beracochea M, Shi ZJ, et al. A unified catalog of 204,938 reference genomes from the human gut microbiome. Nature Biotechnology. 2021;39(11):105–114. doi:10.1038/s41587-020-0603-3.

9. Nayfach S, Rodriguez-Mueller B, Garud N, Pollard KS. An integrated metagenomics pipeline for strain profiling reveals novel patterns of bacterial transmission and biogeography. Genome Research. 2016;doi:10.1101/gr.201863.115.

10. Hanage WP. Not So Simple After All: Bacteria, Their Population Genetics, and Recombination. Cold Spring Harbor Perspectives in Biology. 2016;8(7):a018069. doi:10.1101/cshperspect.a018069.

11. García-López M, Meier-Kolthoff JP, Tindall BJ, Gronow S, Woyke T, Kyrpides NC, et al. Analysis of 1,000 Type-Strain Genomes Improves Taxonomic Classification of Bacteroidetes. Frontiers in Microbiology. 2019;10.

12. Dixit PD, Pang TY, Maslov S. Recombination-Driven Genome Evolution and Stability of Bacterial Species. Genetics. 2017;207(1):281–295. doi:10.1534/genetics.117.300061.

13. Sakoparnig T, Field C, van Nimwegen E. Whole genome phylogenies reflect the distributions of recombination rates for many bacterial species. eLife. 2021;10:e65366. doi:10.7554/eLife.65366.

14. Virtanen P, Gommers R, Oliphant TE, Haberland M, Reddy T, Cournapeau D, et al. SciPy 1.0: Fundamental Algorithms for Scientific Computing in Python. Nature Methods. 2020;17:261–272. doi:10.1038/s41592-019-0686-2.

15. Dixit PD, Pang TY, Studier FW, Maslov S. Recombinant transfer in the basic genome of Escherichia coli. Proceedings of the National Academy of Sciences. 2015;112(29):9070–9075. doi:10.1073/pnas.1510839112.

16. Vos M, Didelot X. A comparison of homologous recombination rates in bacteria and archaea. The ISME Journal. 2009;3(22):199–208. doi:10.1038/ismej.2008.93.

17. Suerbaum S, Smith JM, Bapumia K, Morelli G, Smith NH, Kunstmann E, et al. Free recombination within Helicobacter pylori. Proceedings of the National Academy of Sciences. 1998;95(21):12619–12624. doi:10.1073/pnas.95.21.12619.

18. Vultos TD, Mestre O, Rauzier J, Golec M, Rastogi N, Rasolofo V, et al. Evolution and Diversity of Clonal Bacteria: The Paradigm of Mycobacterium tuberculosis. PLOS ONE. 2008;3(2):e1538. doi:10.1371/journal.pone.0001538.

19. Jain C, Rodriguez-R LM, Phillippy AM, Konstantinidis KT, Aluru S. High throughput ANI analysis of 90K prokaryotic genomes reveals clear species boundaries. Nature Communications. 2018;9(11):5114. doi:10.1038/s41467-018-07641-9.

20. Marçais G, Delcher AL, Phillippy AM, Coston R, Salzberg SL, Zimin A. MUMmer4: A fast and versatile genome alignment system. PLOS Computational Biology. 2018;14(1):e1005944. doi:10.1371/journal.pcbi.1005944.

21. Didelot X, Wilson DJ. ClonalFrameML: Efficient Inference of Recombination in Whole Bacterial Genomes. PLOS Computational Biology. 2015;11(2):e1004041. doi:10.1371/journal.pcbi.1004041.

22. Croucher NJ, Page AJ, Connor TR, Delaney AJ, Keane JA, Bentley SD, et al. Rapid phylogenetic analysis of large samples of recombinant bacterial whole genome sequences using Gubbins. Nucleic Acids Research. 2015;43(3):e15–e15. doi:10.1093/nar/gku1196.

23. Harris K, Nielsen R. Error-prone polymerase activity causes multinucleotide mutations in humans. Genome Research. 2014;24(9):1445–1454. doi:10.1101/gr.170696.113.

24. Durbin R, Eddy SR, Krogh A, Mitchison G. Biological Sequence Analysis: Probabilistic Models of Proteins and Nucleic Acids. Cambridge University Press; 1998.

25. Cleveland WS. Robust Locally Weighted Regression and Smoothing Scatterplots. Journal of the American Statistical Association. 1979;74(368):829–836. doi:10.1080/01621459.1979.10481038.

26. Seabold S, Perktold J. statsmodels: Econometric and statistical modeling with python. In: 9th Python in Science Conference; 2010.

27. Smith JM, Smith NH, O'Rourke M, Spratt BG. How clonal are bacteria? Proceedings of the National Academy of Sciences. 1993;90(10):4384–4388. doi:10.1073/pnas.90.10.4384.

28. Fraser C, Hanage WP, Spratt BG. Neutral microepidemic evolution of bacterial pathogens. Proceedings of the National Academy of Sciences. 2005;102(6):1968–1973. doi:10.1073/pnas.0406993102.

29. Roach DJ, Burton JN, Lee C, Stackhouse B, Butler-Wu SM, Cookson BT, et al. A Year of Infection in the Intensive Care Unit: Prospective Whole Genome Sequencing of Bacterial Clinical Isolates Reveals Cryptic Transmissions and Novel Microbiota. PLOS Genetics. 2015;11(7):e1005413. doi:10.1371/journal.pgen.1005413.

30. Robinson DA, Enright MC. Evolution of Staphylococcus aureus by Large Chromosomal Replacements. Journal of Bacteriology. 2004;186(4):1060–1064. doi:10.1128/JB.186.4.1060-1064.2004.

31. Brochet M, Rusniok C, Couvé E, Dramsi S, Poyart C, Trieu-Cuot P, et al. Shaping a bacterial genome by large chromosomal replacements, the evolutionary history of Streptococcus agalactiae. Proceedings of the National Academy of Sciences. 2008;105(41):15961–15966. doi:10.1073/pnas.0803654105.

32. Fraser C, Hanage WP, Spratt BG. Recombination and the Nature of Bacterial Speciation. Science. 2007;315(5811):476–480. doi:10.1126/science.1127573.

33. Sender R, Fuchs S, Milo R. Revised Estimates for the Number of Human and Bacteria Cells in the Body. PLOS Biology. 2016;14(8):e1002533. doi:10.1371/journal.pbio.1002533.

34. Sung W, Ackerman MS, Miller SF, Doak TG, Lynch M. Drift-barrier hypothesis and mutation-rate evolution. Proceedings of the National Academy of Sciences. 2012;109(45):18488–18492. doi:10.1073/pnas.1216223109.

35. Nayfach S, Pollard KS. Average genome size estimation improves comparative metagenomics and sheds light on the functional ecology of the human microbiome. Genome Biology. 2015;16(1):51. doi:10.1186/s13059-015-0611-7.

36. Savageau MA. Escherichia coli Habitats, Cell Types, and Molecular Mechanisms of Gene Control. The American Naturalist. 1983;122(6):732–744. doi:10.1086/284168.

37. Ghosh OM, Good BH. Emergent evolutionary forces in spatial models of luminal growth and their application to the human gut microbiota. Proceedings of the National Academy of Sciences. 2022;119(28):e2114931119. doi:10.1073/pnas.2114931119.

38. Zhou Z, McCann A, Weill FX, Blin C, Nair S, Wain J, et al. Transient Darwinian selection in Salmonella enterica serovar Paratyphi A during 450 years of global spread of enteric fever. Proceedings of the National Academy of Sciences. 2014;111(33):12199–12204. doi:10.1073/pnas.1411012111.

39. Yahara K, Didelot X, Ansari MA, Sheppard SK, Falush D. Efficient Inference of Recombination Hot Regions in Bacterial Genomes. Molecular Biology and Evolution. 2014;31(6):1593–1605. doi:10.1093/molbev/msu082.

40. Yahara K, Didelot X, Jolley KA, Kobayashi I, Maiden MCJ, Sheppard SK, et al. The Landscape of Realized Homologous Recombination in Pathogenic Bacteria. Molecular Biology and Evolution. 2016;33(2):456–471. doi:10.1093/molbev/msv237.

41. Calland JK, Pascoe B, Bayliss SC, Mourkas E, Berthenet E, Thorpe HA, et al. Quantifying bacterial evolution in the wild: A birthday problem for Campylobacter lineages. PLOS Genetics. 2021;17(9):e1009829. doi:10.1371/journal.pgen.1009829.

42. Lin M, Kussell E. Inferring bacterial recombination rates from large-scale sequencing datasets. Nature Methods. 2019;16(22):199–204. doi:10.1038/s41592-018-0293-7.

43. Didelot X, Méric G, Falush D, Darling AE. Impact of homologous and non-homologous recombination in the genomic evolution of Escherichia coli. BMC Genomics. 2012;13(1):256. doi:10.1186/1471-2164-13-256.

44. Yang C, Pei X, Wu Y, Yan L, Yan Y, Song Y, et al. Recent mixing of Vibrio parahaemolyticus populations. The ISME Journal. 2019;13(1010):2578–2588. doi:10.1038/s41396-019-0461-5.

45. Arevalo P, VanInsberghe D, Elsherbini J, Gore J, Polz MF. A Reverse Ecology Approach Based on a Biological Definition of Microbial Populations. Cell. 2019;178(4):820–834.e14. doi:10.1016/j.cell.2019.06.033.

46. Didelot X, Bowden R, Street T, Golubchik T, Spencer C, McVean G, et al. Recombination and Population Structure in Salmonella enterica. PLOS Genetics. 2011;7(7):e1002191. doi:10.1371/journal.pgen.1002191.

47. Cui Y, Yang X, Didelot X, Guo C, Li D, Yan Y, et al. Epidemic Clones, Oceanic Gene Pools, and Eco-LD in the Free Living Marine Pathogen Vibrio parahaemolyticus. Molecular Biology and Evolution. 2015;32(6):1396–1410. doi:10.1093/molbev/msv009.

48. Korem T, Zeevi D, Suez J, Weinberger A, Avnit-Sagi T, Pompan-Lotan M, et al. Growth dynamics of gut microbiota in health and disease inferred from single metagenomic samples. Science. 2015;349(6252):1101–1106. doi:10.1126/science.aac4812.

49. Harris K, Nielsen R. Inferring Demographic History from a Spectrum of Shared Haplotype Lengths. PLoS Genetics. 2013;9(6):e1003521. doi:10.1371/journal.pgen.1003521.

50. Sabeti PC, Reich DE, Higgins JM, Levine HZP, Richter DJ, Schaffner SF, et al. Detecting recent positive selection in the human genome from haplotype structure. Nature. 2002;419(69096909):832–837. doi:10.1038/nature01140.

51. Garud NR, Messer PW, Buzbas EO, Petrov DA. Recent Selective Sweeps in North American Drosophila melanogaster Show Signatures of Soft Sweeps. PLOS Genetics. 2015;11(2):e1005004. doi:10.1371/journal.pgen.1005004.

52. De Maio N, Wilson DJ. The Bacterial Sequential Markov Coalescent. Genetics. 2017;206(1):333–343. doi:10.1534/genetics.116.198796.

53. Zhao S, Lieberman TD, Poyet M, Kauffman KM, Gibbons SM, Groussin M, et al. Adaptive Evolution within Gut Microbiomes of Healthy People. Cell Host & Microbe. 2019;25(5):656–667.e8. doi:10.1016/j.chom.2019.03.007.

54. Lawson DJ, Hellenthal G, Myers S, Falush D. Inference of Population Structure using Dense Haplotype Data. PLOS Genetics. 2012;8(1):e1002453. doi:10.1371/journal.pgen.1002453.

55. Zou Y, Xue W, Luo G, Deng Z, Qin P, Guo R, et al. 1,520 reference genomes from cultivated human gut bacteria enable functional microbiome analyses. Nature Biotechnology. 2019;37(22):179–185. doi:10.1038/s41587-018-0008-8.

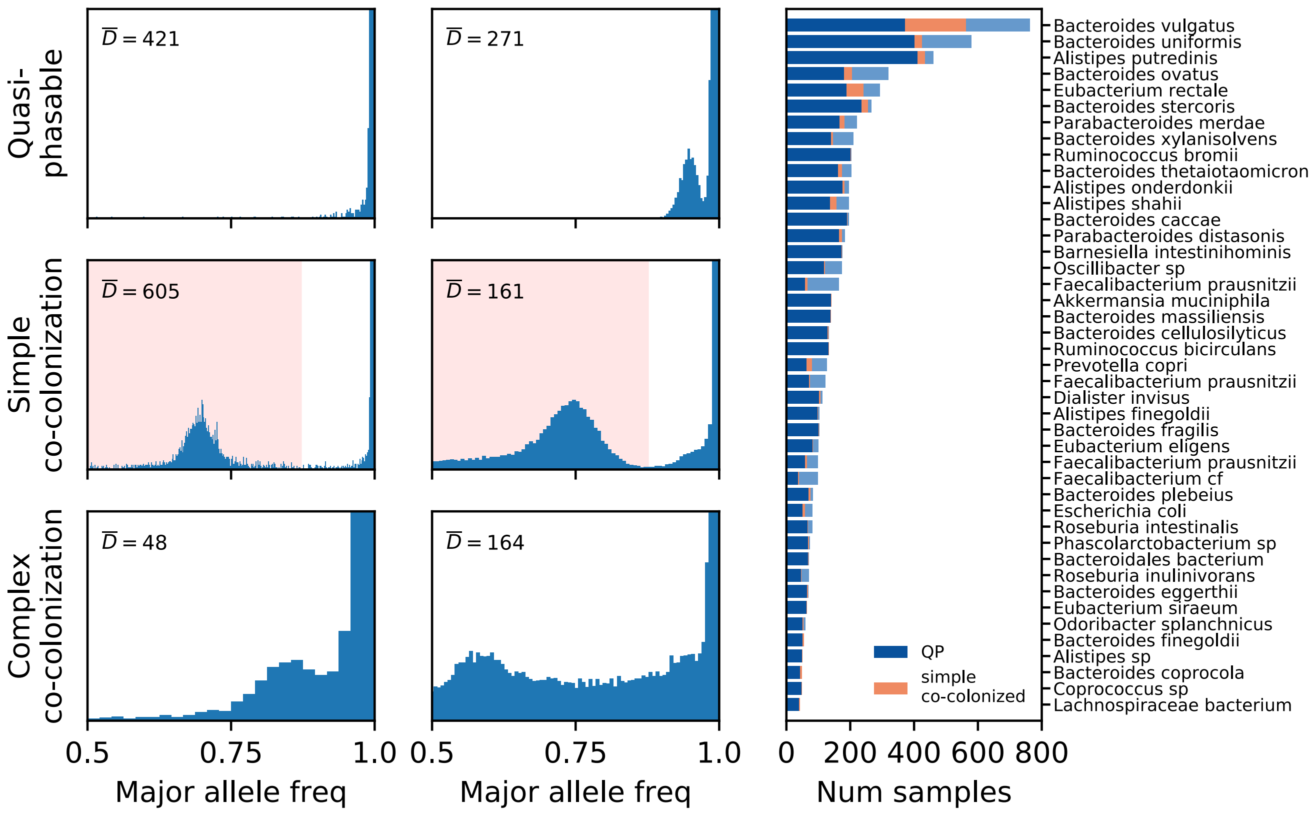

Fig A. Inferring host colonization structure from the within-host site frequency spectrum. Left: Example site frequency spectra of 6 *B. vulgauts* samples from 6 unrelated hosts, showing only synonymous sites in the core genome. Quasi-phaseable (QP) samples (top) are dominated by a single strain of a given species [1]. Simple co-colonized samples (middle) are colonized by two major strains at intermediate frequencies. In this case, genetic differences between the two major strains can be reliably inferred (sites highlighted in pink regions; S1 Text 4.1). More complicated examples that do not fall into these two categories (bottom) were discarded from further analysis. In each of the panels, the vertical axis is scaled by an arbitrary constant and truncated to emphasize the peak at intermediate frequencies; the median read depth ($\overline{D}$) is also listed for reference. Right: The distribution of QP samples and simple co-colonized samples among the 43 species studied. Species are sorted by the total number of samples. The data underlying this Figure can be found in https://doi.org/10.5281/zenodo.10304481

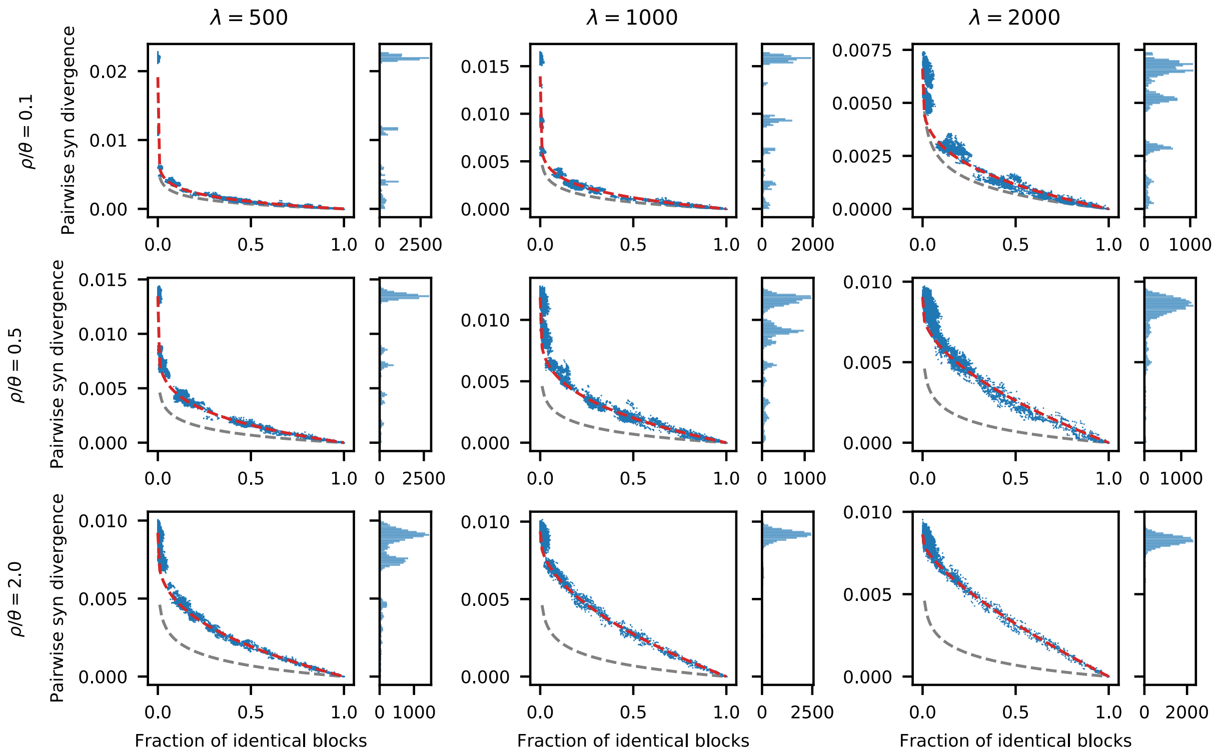

Fig B. Joint distributions of pairwise diversity statistics in a simple neutral model. Analogous versions of Fig 1C, E computed for simulated data from FastSimBac (S1 Text 5.3). Three sets of recombination lengths (λ) and scaled recombination rates (ρ) are shown. The partial recombination model (red lines) provides a good fit to the joint distribution of pairwise statistics in all parameter combinations, while larger values of $\lambda\rho/\theta$ (i.e. stronger recombination) leads to larger deviation from the random expectation (grey lines). The data underlying this Figure can be found in https://doi.org/10.5281/zenodo.10304481

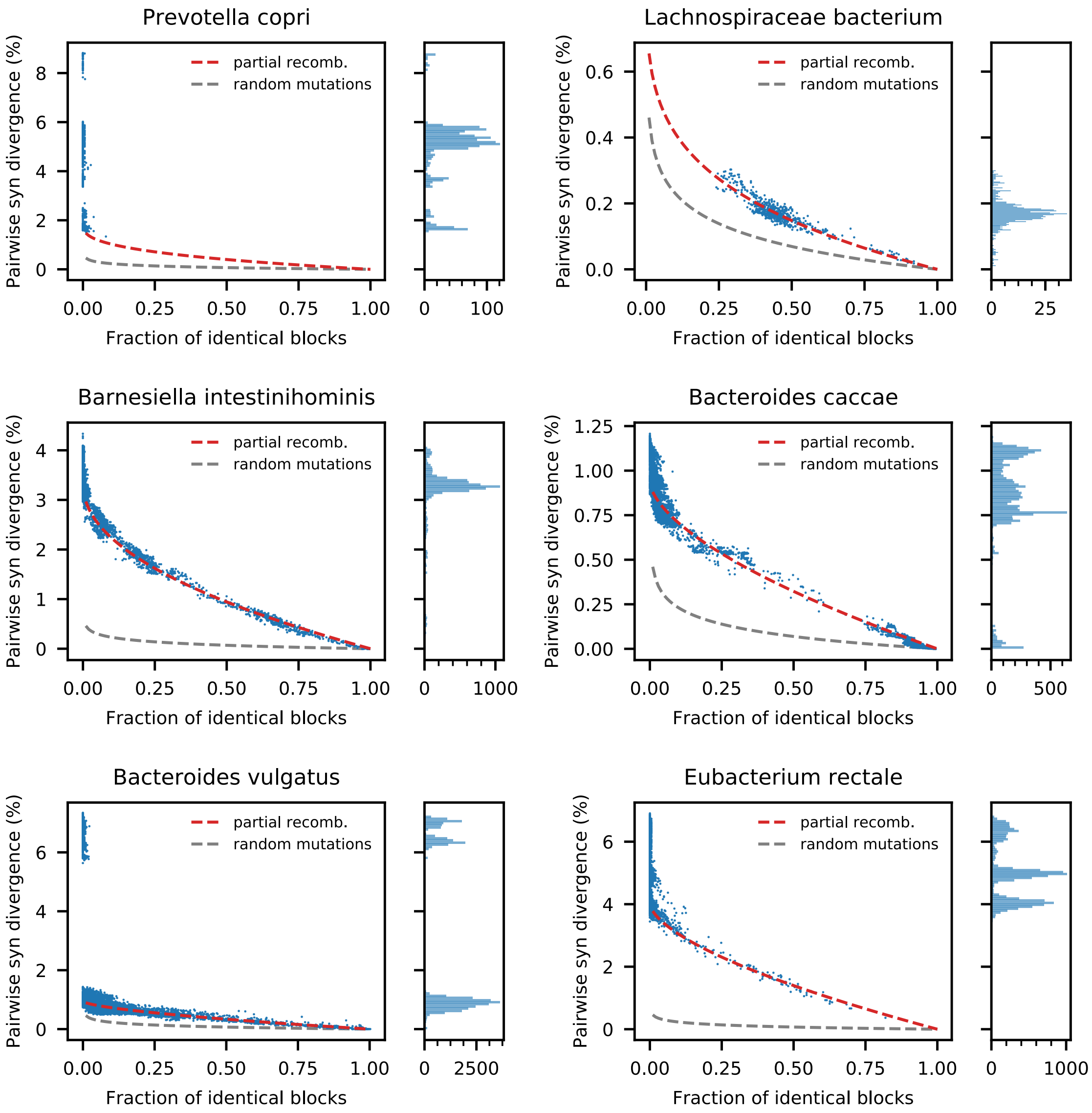

Fig C. Joint distributions of pairwise diversity statistics for six example species. Analogous versions of Fig 1C for six additional example species, which were chosen to illustrate a range of characteristic behaviors. Top panels show example species that have different distributions of partially recombined genomes: *Prevotella copri* lacks closely related pairs almost completely, while all pairs in *Lachnospiraceae bacterium* have identical blocks covering ¿25% of the genome. In this case, the joint distribution reveals distinct behaviors that are difficult to infer based on the genome-wide divergence distribution alone. Middle panels show example species with typical numbers of closely related pairs and minimal population structure: *Barnesiella intestinihominis* has a tightly clustered distribution, akin to *Alistipes putredinis* in Fig 1B, while *Bacteroides caccae* possesses uneven distribution with a significant fraction of extremely close pairs. Bottom panels show example species with different degrees of population structure: *Bacterides vulgatus* features two major clades with 7% synonymous divergence, while *Eubacterium rectale* shows a less clearly separable population structure. The data underlying this Figure can be found in https://doi.org/10.5281/zenodo.10304481

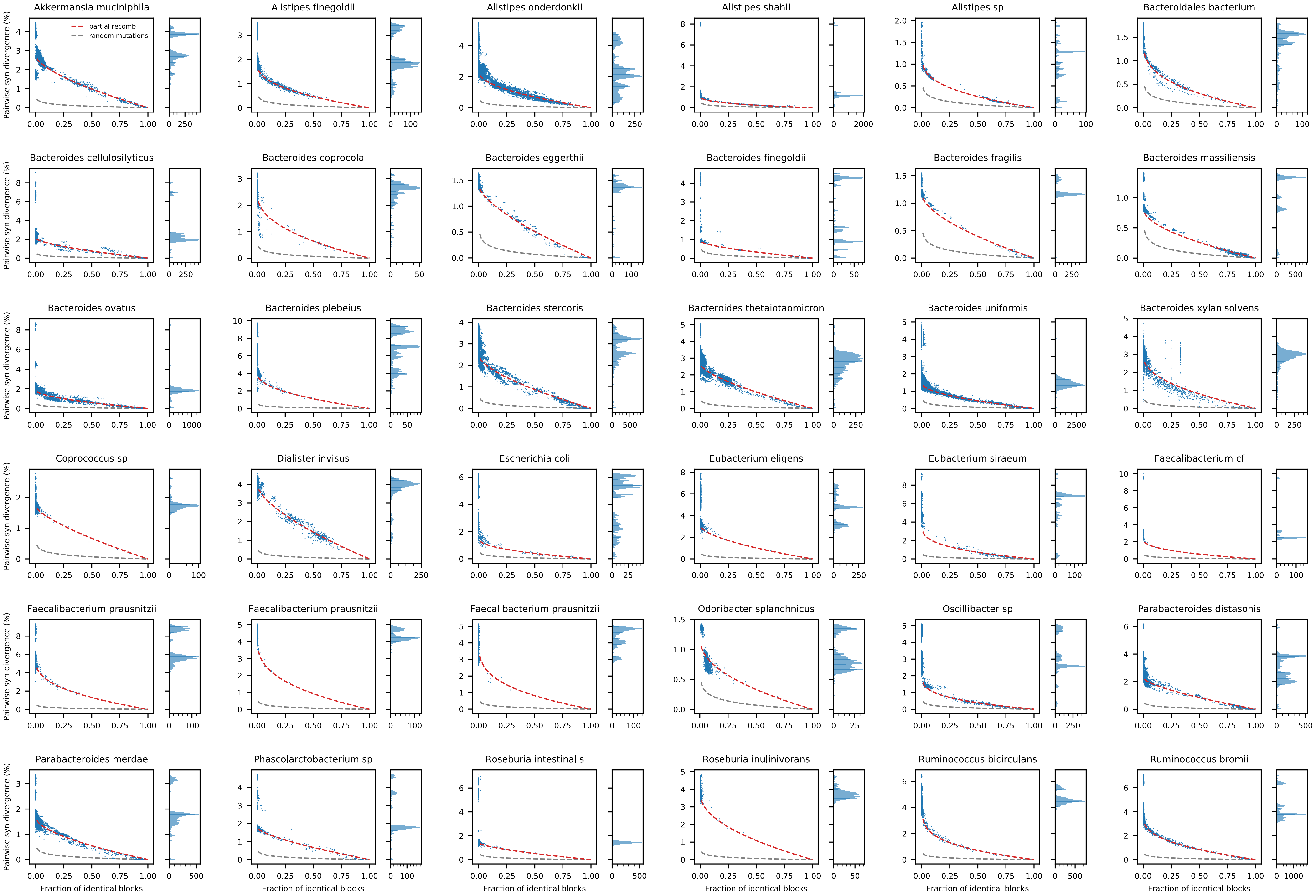
Akkermansia muciniphila
partial recomb.
random mutations
Alistipes finegoldii
Alistipes onderdonkii
Alistipes shahii
Alistipes sp
Bacteroidales bacterium
Bacteroides cellulosilyticus
Bacteroides coprocola
Bacteroides eggerthii
Bacteroides finegoldii
Bacteroides fragilis
Bacteroides massiliensis
Bacteroides ovatus
Bacteroides plebeius
Bacteroides stercoris
Bacteroides thetaiotaomicron
Bacteroides uniformis
Bacteroides xylanisolvens
Coprococcus sp
Dialister invisus
Escherichia coli
Eubacterium eligens
Eubacterium siraeum
Faecalibacterium cf
Faecalibacterium prausnitzii
Faecalibacterium prausnitzii
Faecalibacterium prausnitzii
Odoribacter splanchnicus
Oscillibacter sp
Parabacteroides distasonis
Parabacteroides merdae
Phascolarctobacterium sp
Roseburia intestinalis
Roseburia inulinivorans
Ruminococcus bicirculans
Ruminococcus bromii
Pairwise syn divergence (%)
Fraction of identical blocks

Fig D. Joint distributions of pairwise diversity statistics for all remaining species. Analogous versions of Figs. 1C & C for all species not previously shown in these figures. The data underlying this Figure can be found in https://doi.org/10.5281/zenodo.10304481

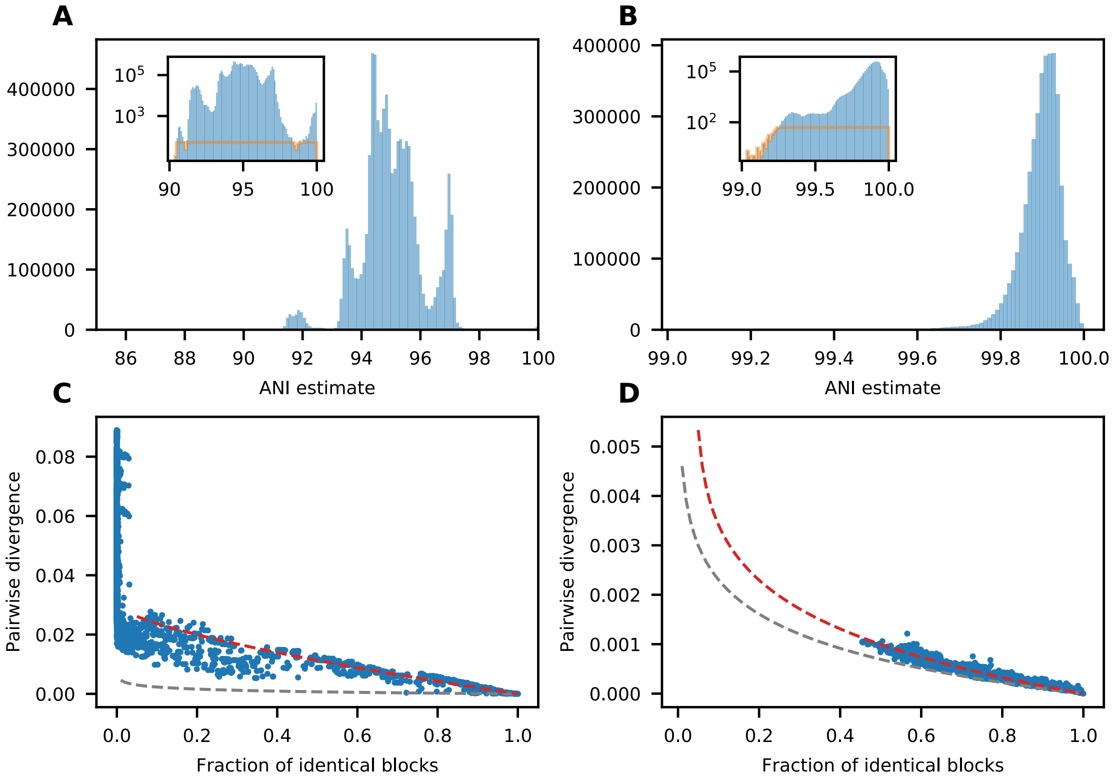

Fig E. Joint distributions of pairwise diversity statistics for two pathogen species shown in Fig 1F. (A-B) The distribution of pairwise ANI estimates for ∼ 2000 strains of *Helicobacter pylori* (A) and *Mycobacterium tuberculosis* (B). Inset: same data on log scale. Orange line denotes the distribution for the subset of data used in panels C & D below, which was downsampled to efficiently represent the full range of pairwise divergence (S1 Text 2.1). (C-D) Analogous versions of Fig 1C for *Helicobacter pylori* (C) and *Mycobacterium tuberculosis* (D). The data underlying this Figure can be found in https://doi.org/10.5281/zenodo.10304481

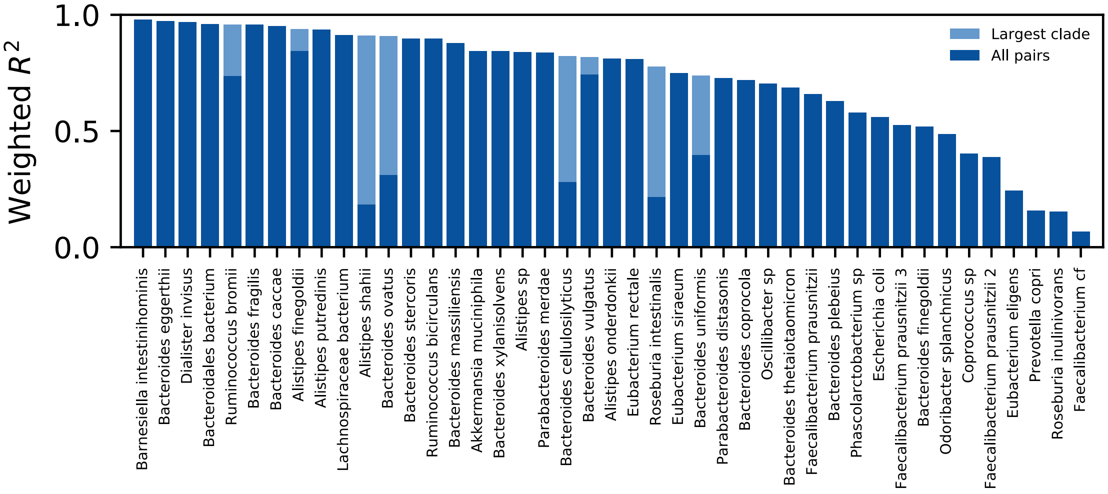

Fig F. Fraction of genome-wide divergence explained by the partial recombination model in different species. Bars show R^2 values obtained by fitting the red line in Figs. 1C & C & D, weighted by the local density of points (S1 Text 2). Dark bars are computed using all pairs of strains, while light bars are restricted to the largest clade in species with strong population structure. The data underlying this Figure can be found in https://doi.org/10.5281/zenodo.10304481

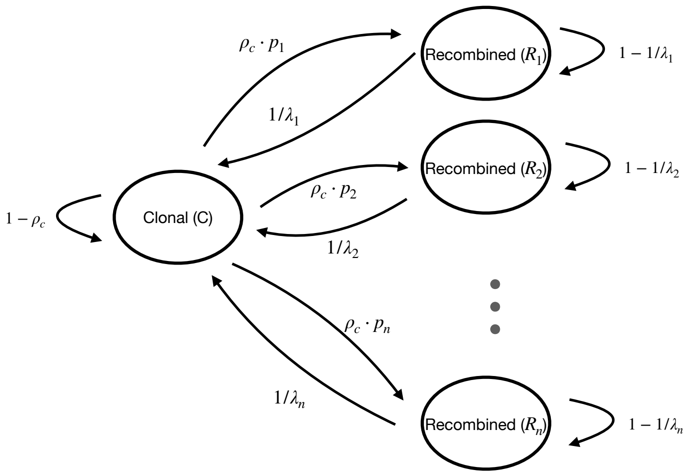

Fig G. Schematic of the hidden states and transition rates in the close-pair HMM. The allowed transitions are sparse, in that each recombined state is only connected to the clonal state. This simplified structure directly follows from the assumption that recombination events rarely overlap for a sufficiently close pair.

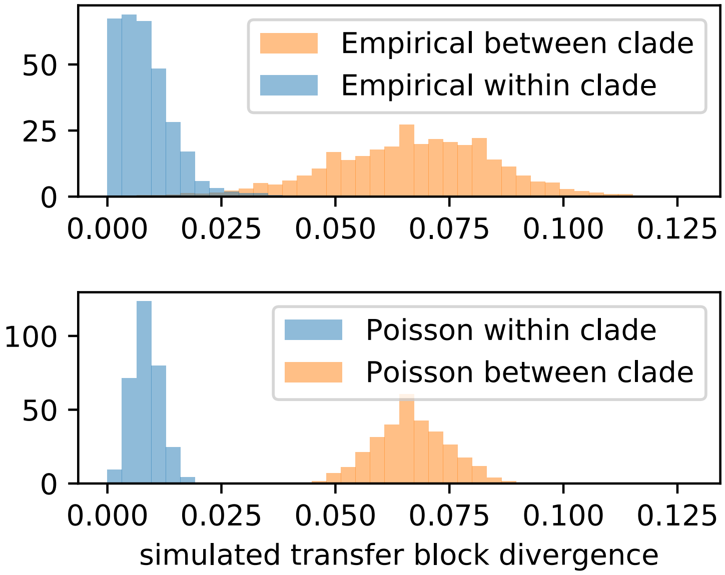

Fig H. Empirical distributions of local divergence are broader than matching Poisson distributions. The top panel shows the empirical distribution of local pairwise divergence in *B. vulgatus*, computed by sampling blocks of 1000 synonymous sites as described in S1 Text 3.1. The bottom panel shows two corresponding Poisson distributions whose means match the empirical distributions above. The Poisson distribution does not capture the broad variation observed in the empirical distribution. The data underlying this Figure can be found in https://doi.org/10.5281/zenodo.10304481

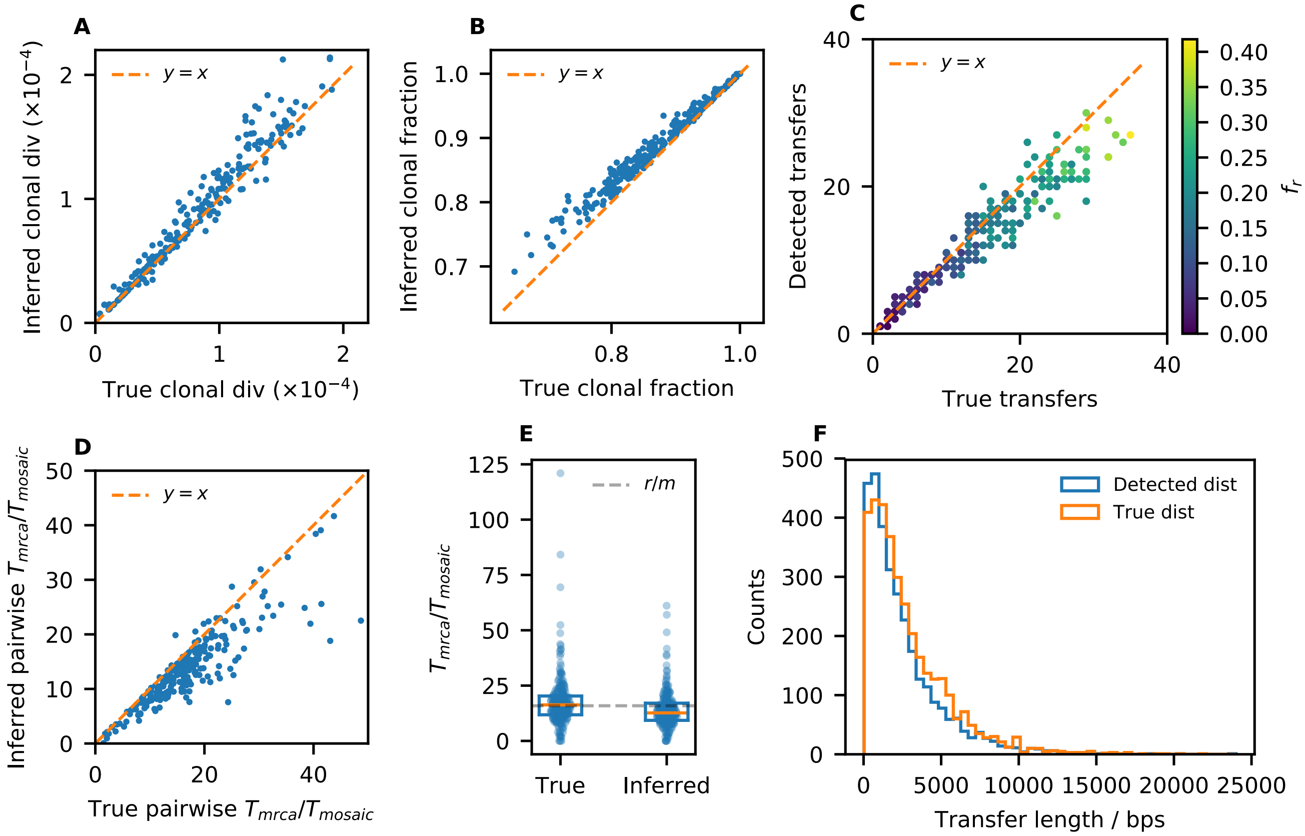

Fig I. Validation of Close-Pair HMM (CP-HMM) algorithm using simulated data. A total of 256 pairs of close strains were simulated across a range of divergence times, using parameters matching *Bacteroides vulgatus* (S1 Text 3.2). (A) Correlation between the inferred clonal divergence and the true clonal divergence, $2\mu T$. (B) Correlation between the inferred clonal fraction and the true clonal fraction. (C) Correlation between the number of detected transfers and the true number of transfers. Colors show the approximate fraction of recombined regions, defined as the true total length of recombination events divided by the genome length (overlapped regions will be counted more than once). (D) Correlation between the inferred $T_{\mathrm{mrca}}/T_{\mathrm{mosaic}}$ and the true $T_{\mathrm{mrca}}/T_{\mathrm{mosaic}}$ for each simulated pair. Panels (A-D) all show good agreement except for the most diverged pairs, which potentially reflect the influence of overlapping transfer events. (E) Distribution of true and inferred $T_{\mathrm{mrca}}/T_{\mathrm{mosaic}}$ for all simulated pairs. Box and orange lines indicate the interquartile range and median, respectively. Grey dashed line indicates the ground truth r/m value calculated from Eq. (S12) using the simulated parameters. These results show the pairwise estimates of r/m (or $T_{\mathrm{mrca}}/T_{\mathrm{mosaic}}$) can span a wide range due to Poisson sampling of mutations and recombination events, even in simulations with a single r/m value. (F) Distribution of detected transfer lengths vs the ground truth, showing good agreement. Detected transfers are expected to be slightly shorter than the ground truth, because the region between the last SNV and the end of a transfer (or between the start and the first SNV) is impossible for the HMM to detect. The data underlying this Figure can be found in https://doi.org/10.5281/zenodo.10304481

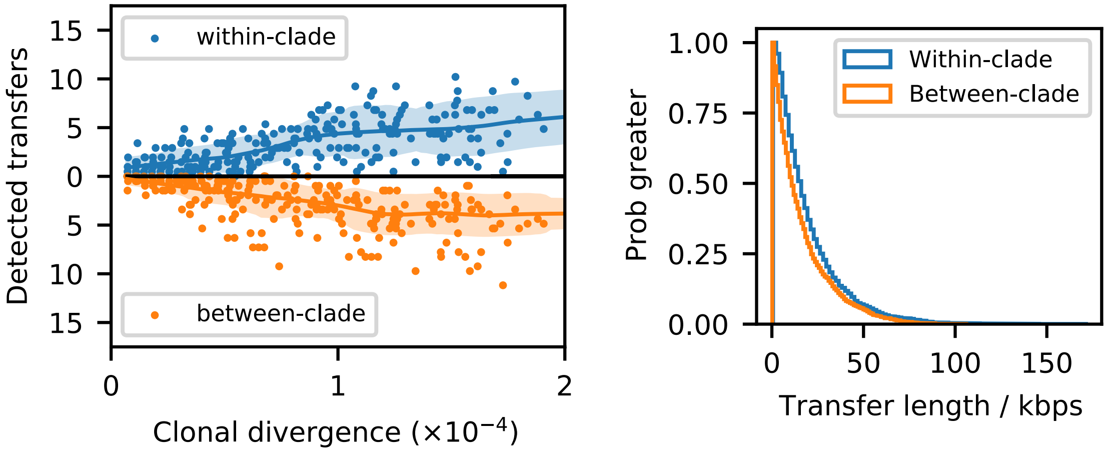

Fig J. Comparing recombination dynamics in *B. vulgatus* using simulated data. Analogous versions of Fig 2C-D created for the simulated data in Fig I (S1 Text 3.2). In the simulation, between-clade and within-clade transfers are set to have the same rate and transfer length distribution. The simulated data therefore allow us to test whether the patterns in Fig 2 were caused by effects such as a detection bias toward toward high divergence transfers, or the size difference between the two *B. vulgatus* clades. The number of detected transfers in the simulated data are comparable between the two transfer types, in contrast to the 5-fold difference in Fig 2C; similarly, there is only a small ($< 30\%$) bias in the length of the between-clade transfers, in contrast to the 7-fold reduction observed in data. The distinct patterns observed in *B. vulgatus* therefore cannot be explained by biases due to divergence or sampling of the two clades. These simulated data also show that using a single recombination rate does not generate large numbers of transfers at short divergence times, in sharp contrast with the observed data in Fig 2C. The data underlying this Figure can be found in https://doi.org/10.5281/zenodo.10304481

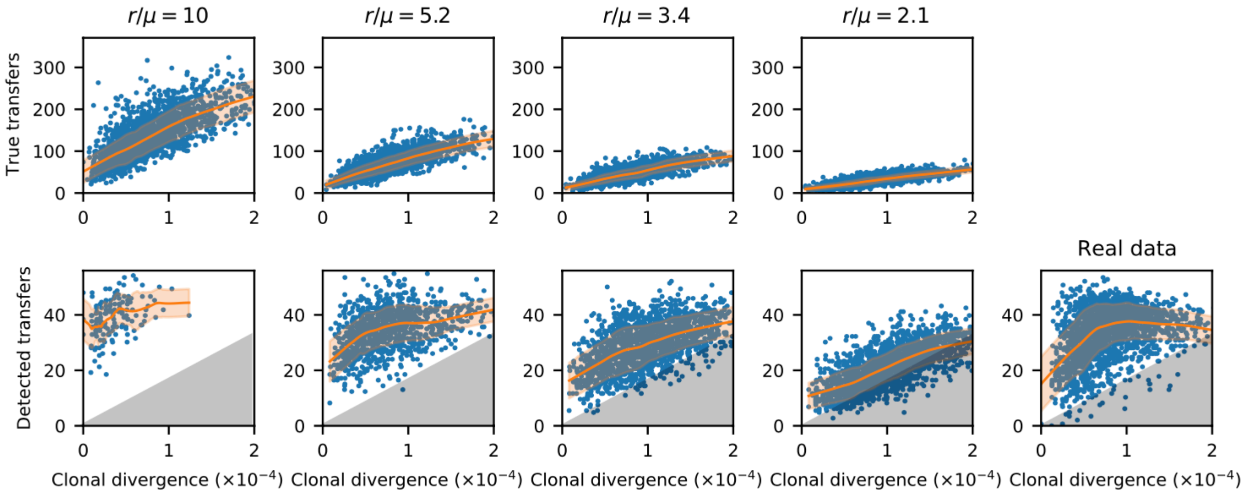

Fig K. CP-HMM results for simulations approximating *A. putredinis*. We performed simulations using three different recombination rates while matching the total number of samples, the genome length and the mean recombination length of *A. putredinis* (S1 Text 3.5). The bottom panels show analogous versions of Fig 3C computed from these simulated datasets; the original data from Fig 3C are reproduced on the right for comparison. The same region of low recombination rate are shaded to highlight the difference between $r/\mu = 5.2$ and real data. The top panels show the true number of transfers versus the true clonal divergence in the simulations (trend lines and confidence intervals are computed in the same manner as Fig 3C). None of the simulated datasets reproduces the broad variation observed in *A. putredinis*, which contains pairs with both large numbers of transfers at short times and small numbers of transfers at long times. This suggests that a single recombination rate cannot explain the observed data. The data underlying this Figure can be found in https://doi.org/10.5281/zenodo.10304481

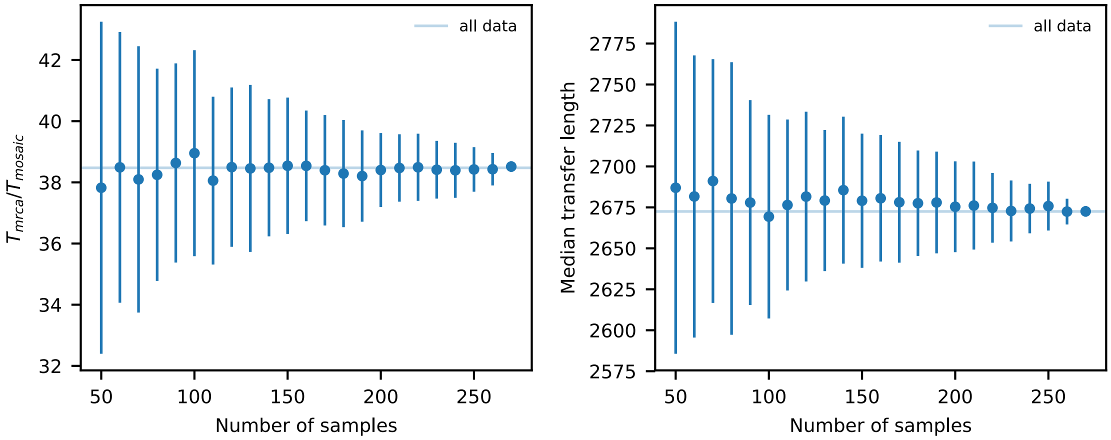

Fig L. Dependence of CP-HMM results on the sample size. A series of downsampling experiments were performed using the *Alistipes putredinis* dataset, and CP-HMM was applied to each of the downsampled dataset. Left and right panels show the inferred $T_{\mathrm{mrca}}/T_{\mathrm{mosaic}}$ ratios (left) and the median inferred transfer lengths (right). Bars denote one standard deviation calculated from 100 replicates. The parameters inferred from the downsampled datasets remain very close to the values obtained from the full dataset. The data underlying this Figure can be found in https://doi.org/10.5281/zenodo.10304481

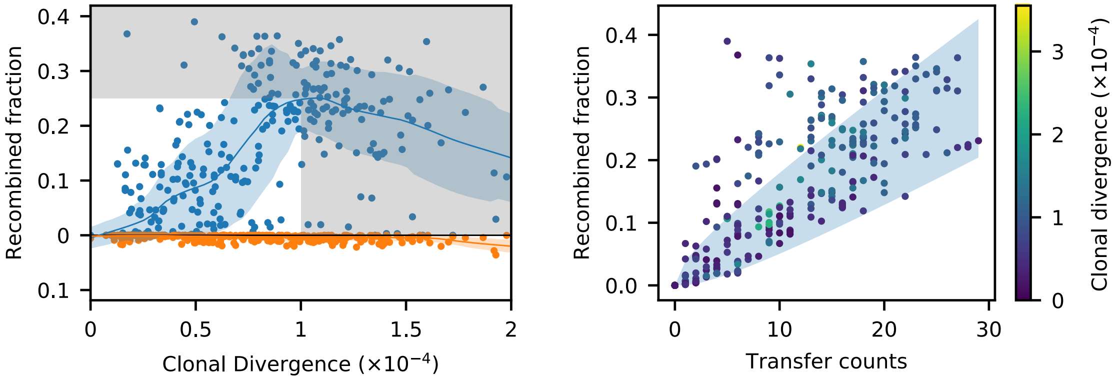

Fig M. Total recombined fraction vs divergence in *B. vulgatus*. Left: analogous version of Fig 2C showing the total fraction of the core genome composed of recombined regions as inferred by CP-HMM. In contrast to the number of transfers, this metric remains consistent when neighboring transfers overlap, providing an alternative check on the general patterns we observed in Fig 2C. Points show the CP-HMM results for all pairs of strains with at least ¿50% identical blocks. Grey regions denote the points that were excluded by our subsequent filtering steps (S1 Text 3.3). Comparing these results with Fig 2C shows that many important trends (e.g. the non-linear accumulation of transfers and the reduced recombination between clades) are also observed in this alternative metric. Right: Scatter plot showing the correlation between the number of detected transfers and the total recombined fraction. Each point is colored by the inferred clonal divergence for that pair. Blue shaded regions denote 95% confidence intervals for a null model where the length of each transfer is drawn from an exponential distribution with mean measured in Fig 2D. This comparison reveals that in some pairs, a small number of transfers account for an unusually large fraction of recombined genome. Examples of these outliers are shown in Fig N. The data underlying this Figure can be found in https://doi.org/10.5281/zenodo.10304481

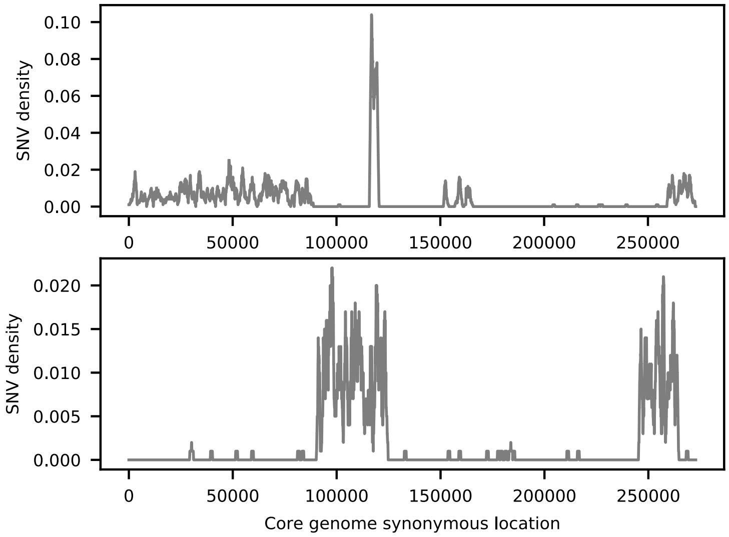

Fig N. Examples of extremely long recombination events. Top and bottom panels show the synonymous divergence profiles of two pairs of closely related *B. vulgatus* strains with abnormally long recombination events. Top panel shows an example of a recombined region covering nearly half of the genome, while leaving extended regions unmodified. This pattern is unlikely to be generated by the merging of multiple independent transfers: for a typical recombination length of ≈2,900 synonymous sites, ≈40 such transfers must occur exclusively within a particular half of the genome ($P < 10^{-12}$). Bottom panel shows an example of two isolated recombination events covering more than ≳50,000 synonymous sites (equivalent to $\sim$ 350kb in total genome length). Some of these long events appear to have multiple peaks in their associated SNV profiles, suggesting that they might be composed of several smaller (correlated) transfers. We also found similar long recombination events between pairs of cultured isolates (Fig O). The data underlying this Figure can be found in https://doi.org/10.5281/zenodo.10304481

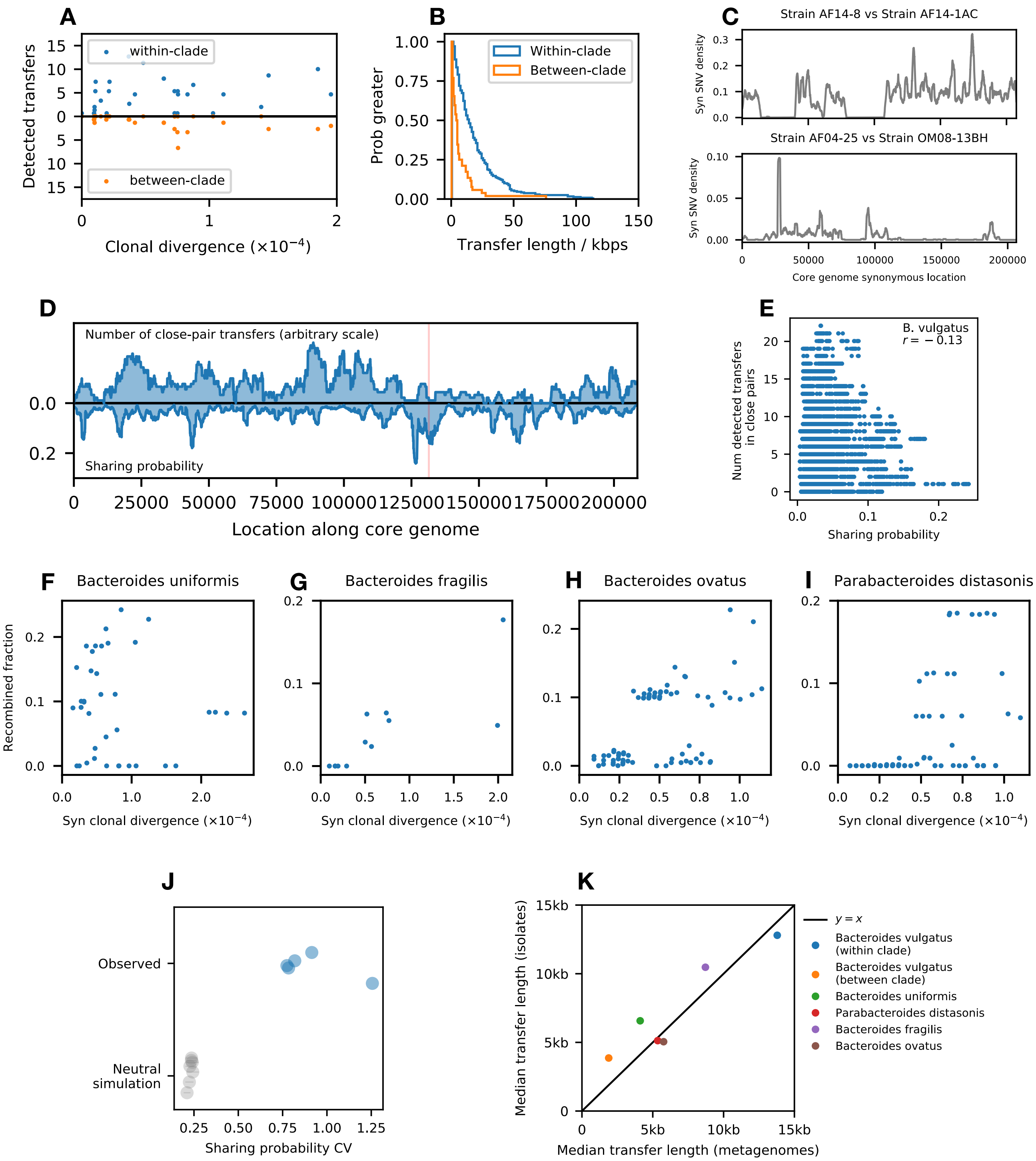

A
within-clade
between-clade
Detected transfers
Clonal divergence (×10^−4)
B
Within-clade
Between-clade
Prob greater
Transfer length / kbps
C
Strain AF14-8 vs Strain AF14-1AC
Strain AF04-25 vs Strain OM08-13BH
Syn SNV density
Core genome synonymous location
D
Number of close-pair transfers (arbitrary scale)
Sharing probability
Location along core genome
E
B. vulgatus
r = −0.13
Num detected transfers in close pairs
Sharing probability
F
Bacteroides uniformis
G
Bacteroides fragilis
H
Bacteroides ovatus
I
Parabacteroides distasonis
Recombined fraction
Syn clonal divergence (×10^−4)
J
Observed
Neutral simulation
Sharing probability CV
K
y = x
Bacteroides vulgatus (within clade)
Bacteroides vulgatus (between clade)
Bacteroides uniformis
Parabacteroides distasonis
Bacteroides fragilis
Bacteroides ovatus
Median transfer length (isolates)
Median transfer length (metagenomes)

Fig O. CP-HMM analysis of isolate genomes from commensal gut species. CP-HMM was applied to a total of 314 previously sequenced isolate genomes from 5 species of commensal gut bacteria (S1 Text 3.10). (A-B) Analogous versions of Fig 2C-D for *Bacteroides vulgatus* isolates. The reduction in the rate and length of between-clade transfers relative to within-clade is quantitatively reproduced in the isolate data. (C) Examples of long putative transfer events in *B. vulgatus*. Both panels show the density of 4D SNVs between a pair of strains along the core genome. All four strains were originally sampled in Ref. 55. Top: Two strains sampled from the same host share two anomalously long segments, while retaining high divergence in the rest of the genome. Bottom: Two closely related strains sampled from unrelated hosts exhibit recombined regions that are concentrated in only half of the genome. This pattern is very similar to Fig N, suggesting a very large recombination event in the time since they last shared a common ancestor. (D-E) Analogous version of Fig JJA-B for *B. vulgatus* isolates. The location of the within-host sweep event in Fig 5C, showing an elevated sharing fraction; note that the location along core genome is different because a different reference genome was used for the isolate analysis. (F-I) Analogous versions of Fig Q obtained from isolate data. The apparent rates of accumulation of recombination events are consistent with our previous results using metagenomic data. (J) Analogous version of Fig 6D. The CV of the sharing probability among isolate genomes is again much higher than neutral simulations. (K) Scatter plot of median transfer length inferred using metagenomic data vs isolates, showing good agreement. The data underlying this Figure can be found in https://doi.org/10.5281/zenodo.10304481

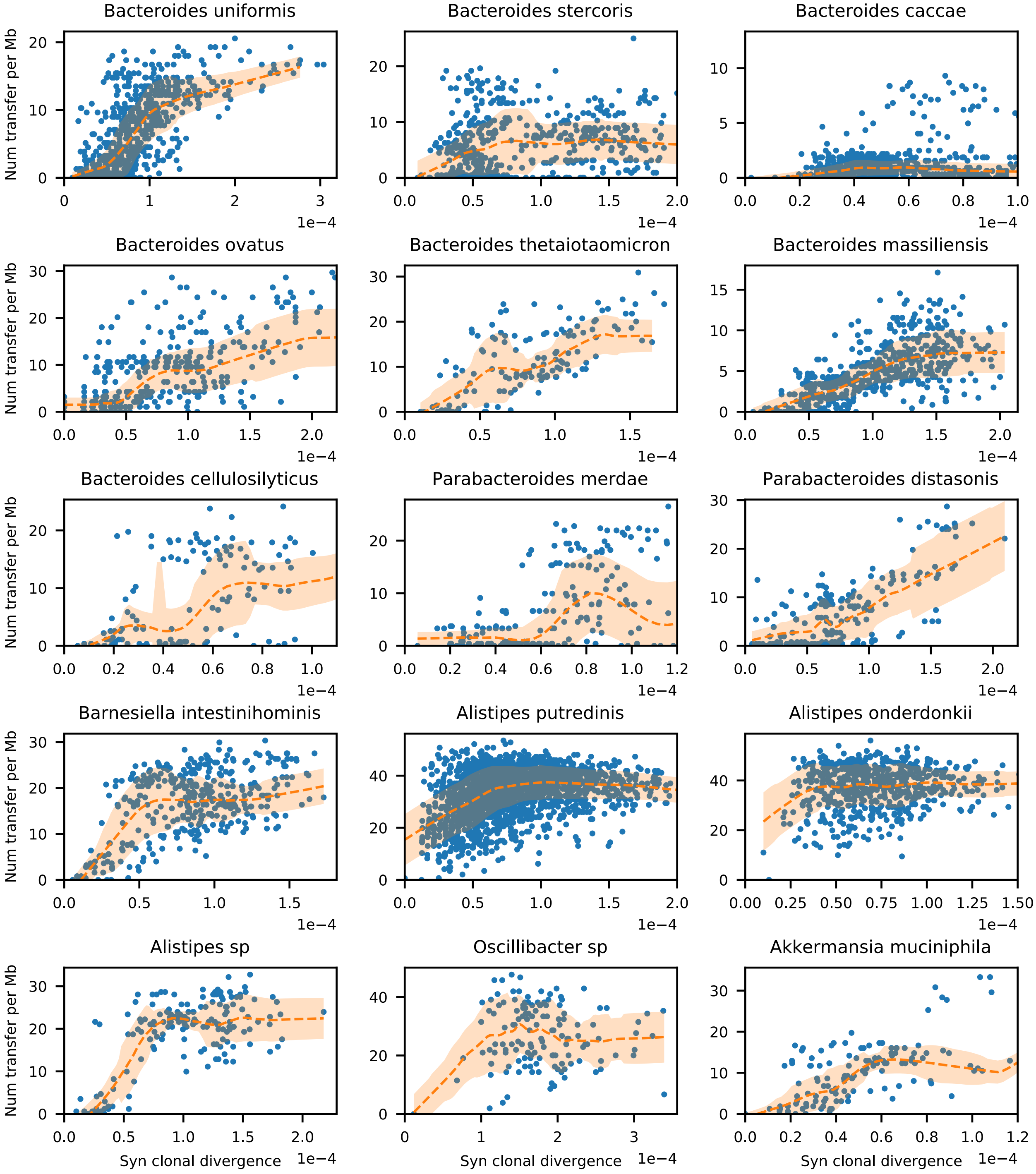

Fig P. Number of transfers vs divergence for other prevalent gut species. Analogous versions of Fig 3A-C for all species with > 100 closely related pairs (i.e. inferred clonal fraction > 75%). *B. vulgatus* is plotted separately in Fig 2. The data underlying this Figure can be found in https://doi.org/10.5281/zenodo.10304481

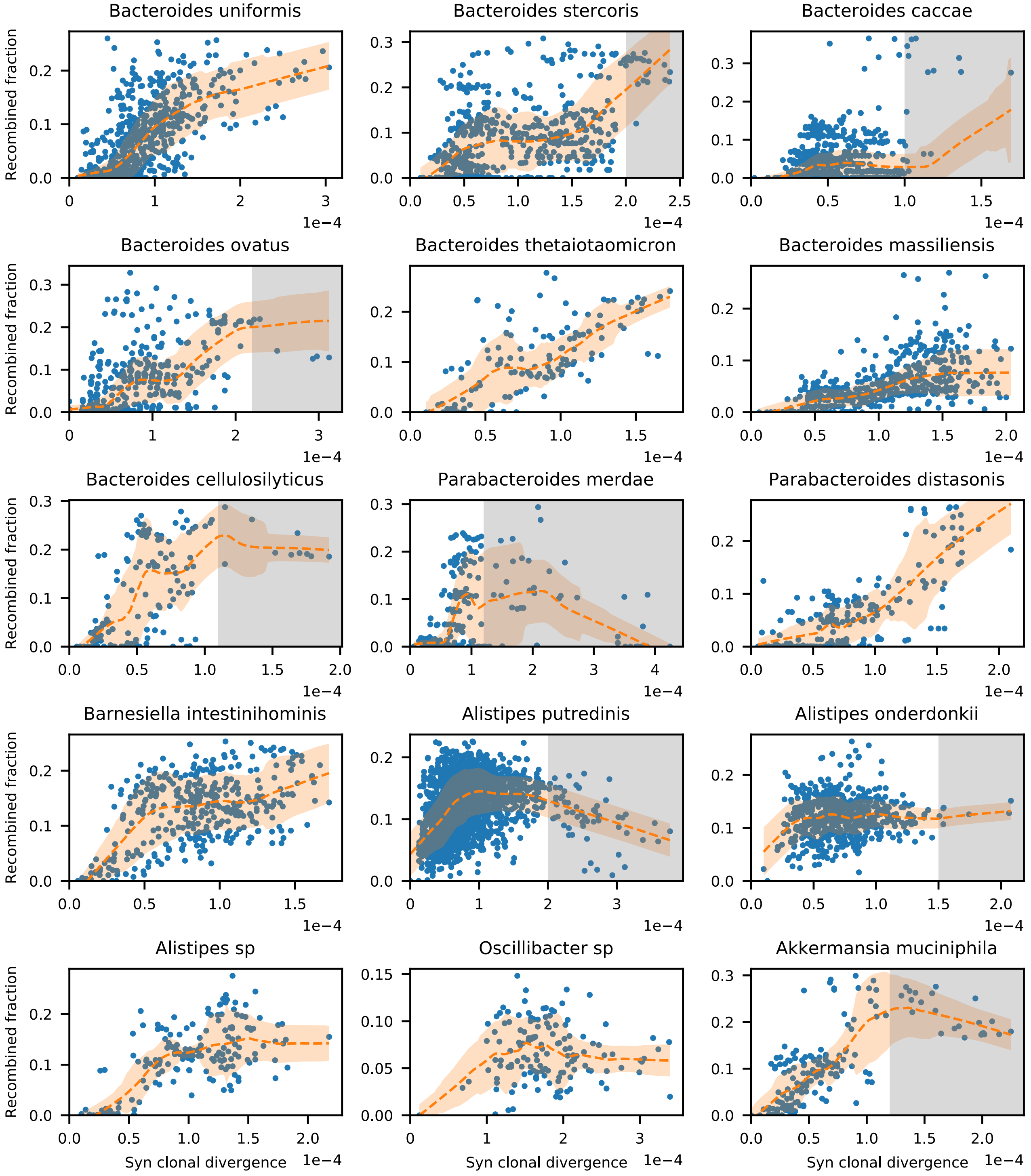

Fig Q. Total recombined fraction vs divergence for other prevalent gut species. Analogous version of Fig P showing the total length of all recombined regions (similar to the *B. vulgatus* example in Fig M). Comparisons with the total number of counts in Fig P show that the overall trends revealed by these two metrics are similar for most species, including key observations such as the high degree of variation in *A. putredinis*. Interestingly, *B. caccae* has a larger spread in this metric than in the number of transfers (Figs 3 & P), which is consistent with the its broad distribution of transfer lengths (Fig 3E). The data underlying this Figure can be found in https://doi.org/10.5281/zenodo.10304481

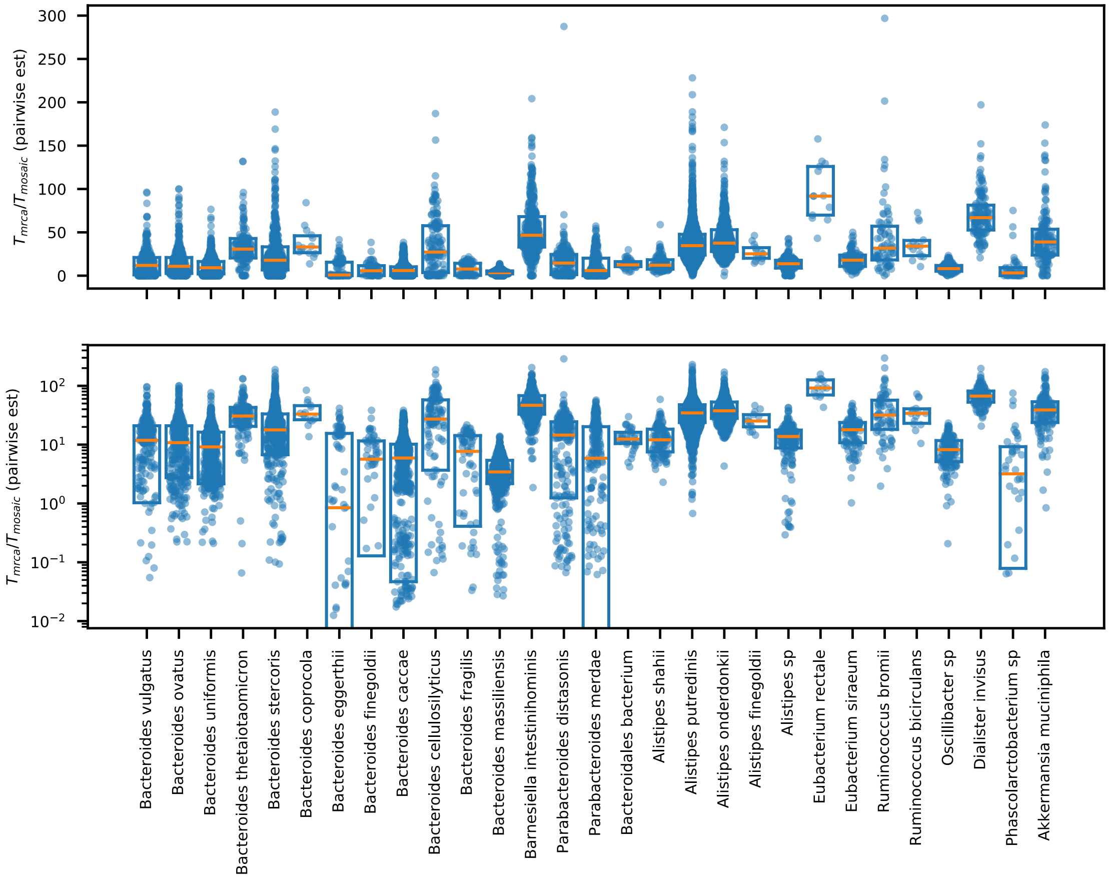

Fig R. Distribution of $T_{\mathrm{mrca}}/T_{\mathrm{mosaic}}$ estimates from different pairs of strains. Top: Symbols show the estimated values of $T_{\mathrm{mrca}}/T_{\mathrm{mosaic}}$ for all close pairs using the expression in Eq. (S20); box plots indicate the median and interquartile range. This metric is roughly equivalent to the "r/m" metric in the context of simple neutral models [15, 16]. Bottom: same data on a log scale. The data underlying this Figure can be found in https://doi.org/10.5281/zenodo.10304481

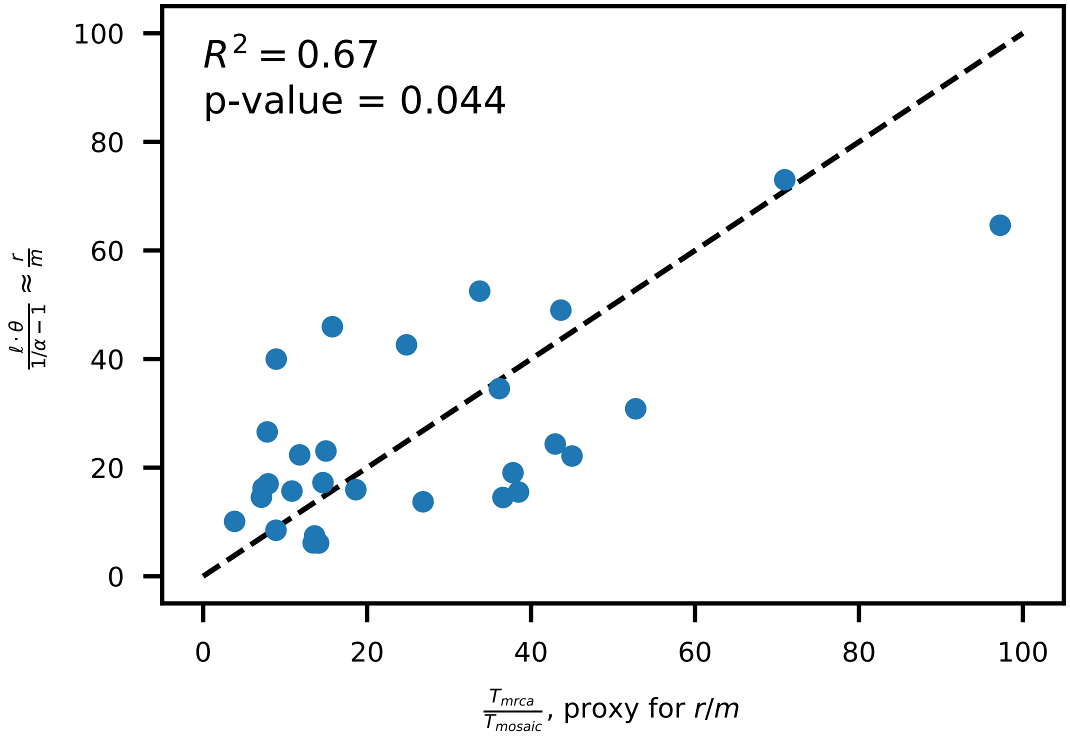

Fig S. Correlation between $T_{\mathrm{mrca}}/T_{\mathrm{mosaic}}$ estimates from two different methods. Horizontal axis shows the $T_{\mathrm{mrca}}/T_{\mathrm{mosaic}}$ estimates from the CP-HMM algorithm in Fig R (S1 Text 3.7), while the vertical axis shows the estimates from the partial recombination model in Fig 1F (S1 Text 2.1). The estimates from the two methods are broadly correlated with each other, but there are quantitative differences that reflect the differences in their underlying averaging schemes. The data underlying this Figure can be found in https://doi.org/10.5281/zenodo.10304481

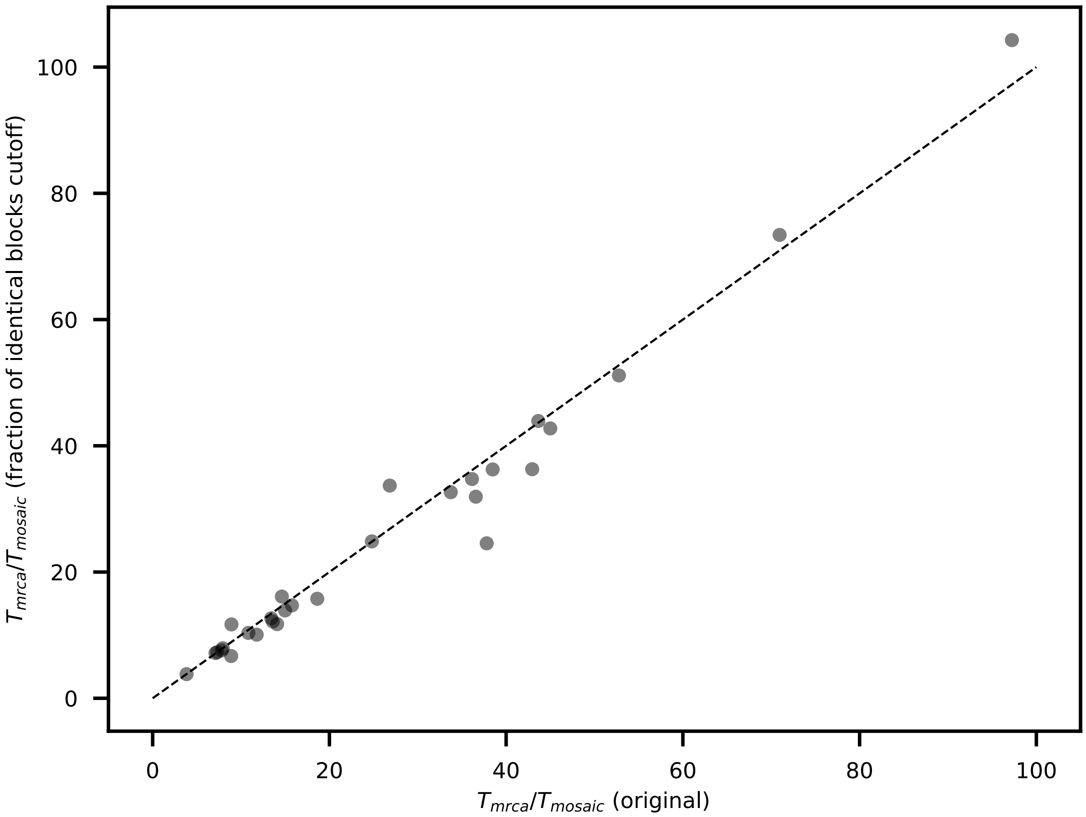

Fig T. Correlation between $T_{\mathrm{mrca}}/T_{\mathrm{mosaic}}$ estimates. Horizontal axis indicates the values of average $T_{\mathrm{mrca}}/T_{\mathrm{mosaic}}$ for each species in Fig R. The vertical axis indicates the average $T_{\mathrm{mrca}}/T_{\mathrm{mosaic}}$ from the same analysis, but instead of using the inferred clonal divergence to filter out pairs where CP-HMM does not perform well, we used fraction of identical blocks, which requires no CP-HMM inference. The two estimates show good agreement. The data underlying this Figure can be found in https://doi.org/10.5281/zenodo.10304481

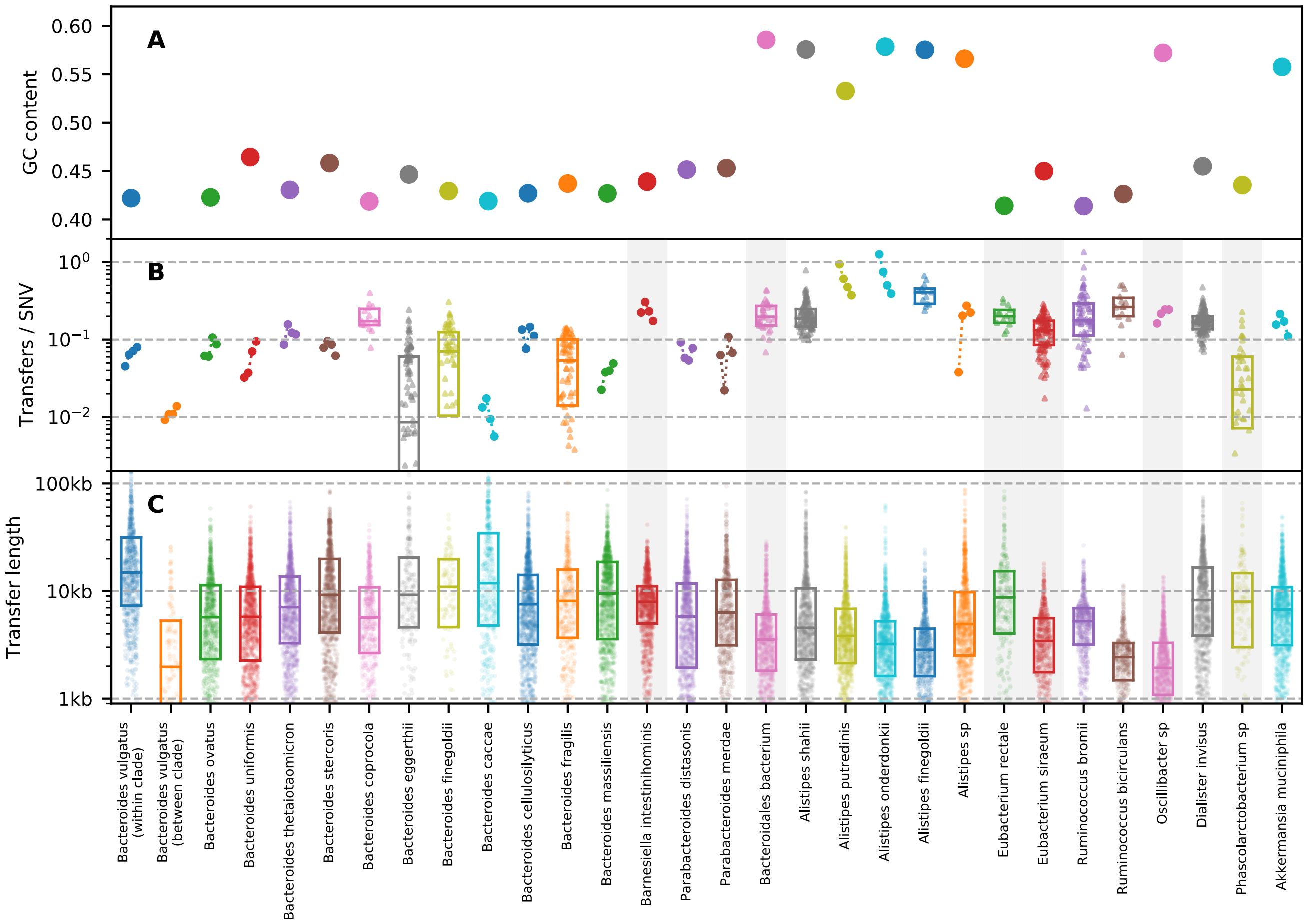

Fig U. Correlation between GC content and recombination paramters. (A) Genome-wide average GC content of gut bacterial species. (B-C) Same as Fig 3D-E. These data show no systematic correlation between GC content and recombination parameter for these species. The data underlying this Figure can be found in https://doi.org/10.5281/zenodo.10304481

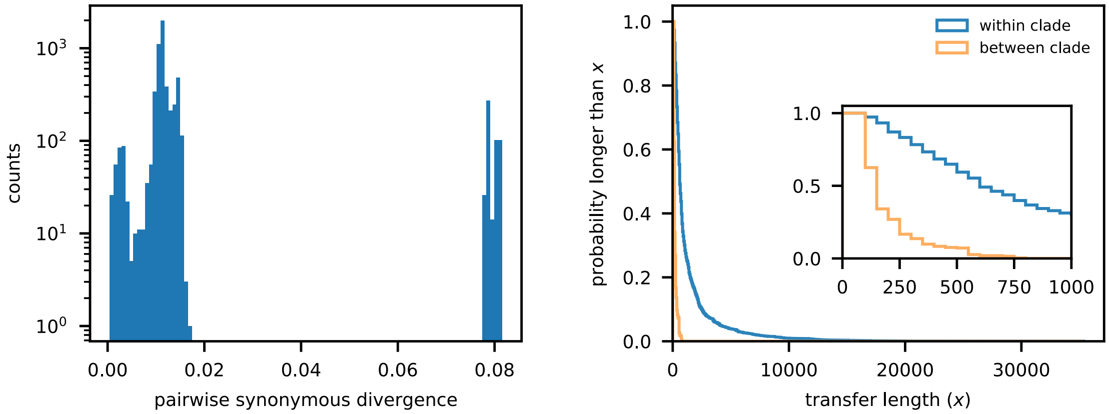

Fig V. Within- and between-clade transfers in *A. shahii*. Left: Pairwise divergence distribution of *A. shahii*. Strains separate clearly into two major clades, similar to *B. vulgatus* (Fig C). Right: Analogous version of Fig 2D constructed for *A. shahii*. As in Fig 2D, within- vs between-clade transfers are inferred based on divergence of the transferred region. Inset shows a zoomed-in version to highlight the between-clade distribution. This shows that the reduction in transfer length for between-clade transfers is not specific to *B. vulgatus*. The data underlying this Figure can be found in https://doi.org/10.5281/zenodo.10304481

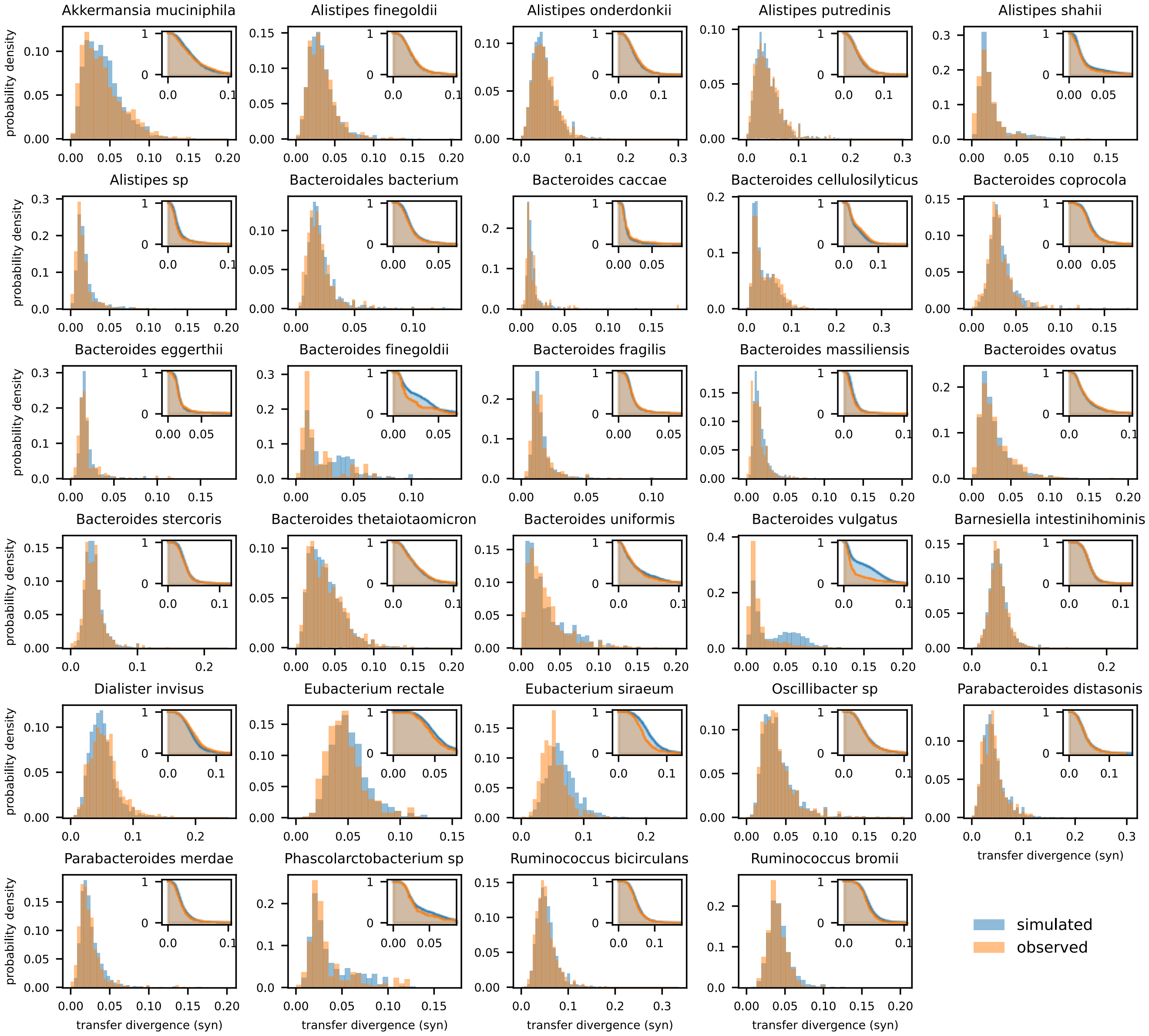

Fig W. Distribution of divergence of detected transfers for each species. Orange histograms show the synonymous divergence of all detected transfers for all species shown in Fig 3. For comparison, the blue distributions show the expectation for a null model in which we simulated transfers in the same set of close pairs by randomly sampling donated sequences from the larger set of genomes in our cohort. To preserve potential variation along the genome, the locations and lengths of the simulated transfers were chosen to match the actual detected events. For most species, the empirical distribution of divergence closely follows this null model, consistent with the picture that the accumulated transfers were obtained from random strains in the population. However, certain species (e.g. *B. vulgatus*, *B. finegoldii*) that have apparent population structure, as indicated by a second peak in the simulated histogram, show a significant decrease in transfers donated from the other clade. Importantly, this decrease of transfer efficiency is not seen for other closely related species with a similar range of transfer divergences (e.g. *B. thetaiotaomicron*). The data underlying this Figure can be found in https://doi.org/10.5281/zenodo.10304481

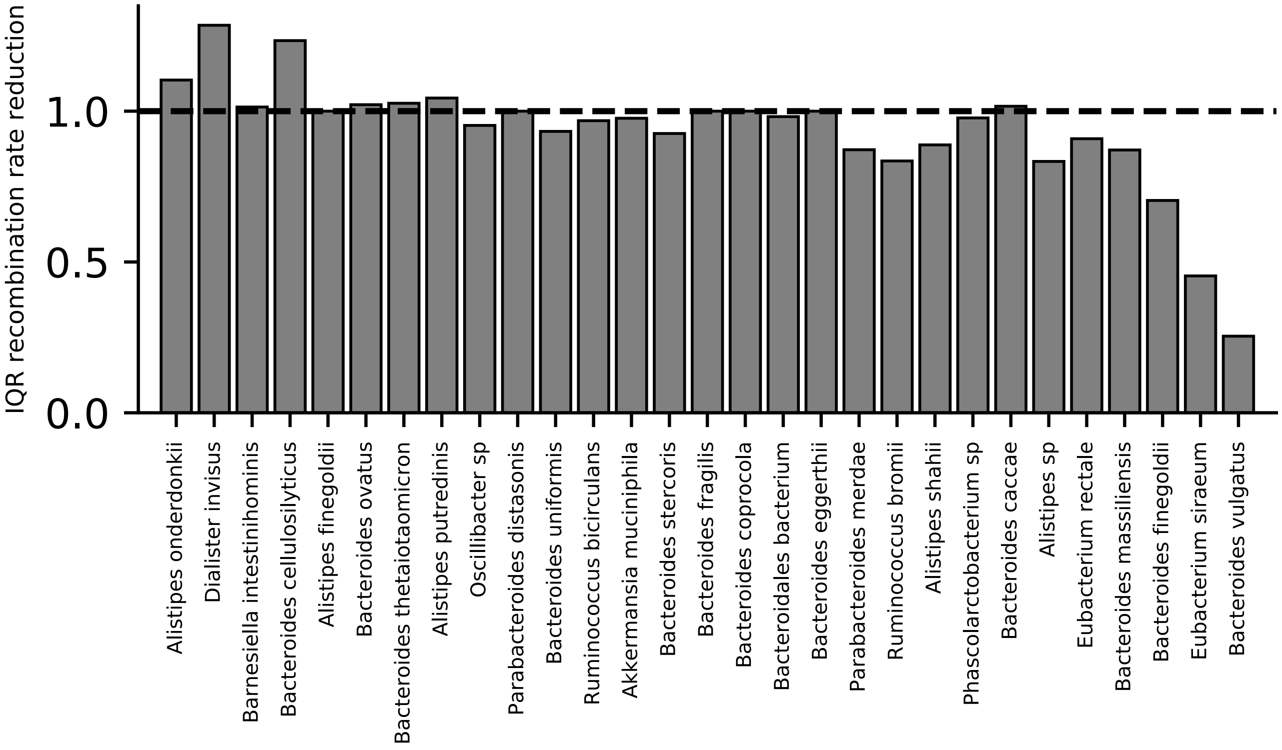

Fig X. Estimates of the reduction in the realized recombination rate as a function of the divergence of the transferred fragment. The observed and expected divergence distributions in Fig W were fit to a simple model of a recombination barrier, where the recombination rate declines exponentially with the local sequence divergence (S1 Text 3.6.1). Vertical axis shows the predicted reduction over the typical spread of transfer divergence (IQR of the simulated distributions in Fig W), which are generally well correlated with the K-S distances in Fig 4. These data suggest that sequence divergence alone does not impose a strong barrier to gene flow within species for most of the gut commensals in Fig 4. The data underlying this Figure can be found in https://doi.org/10.5281/zenodo.10304481

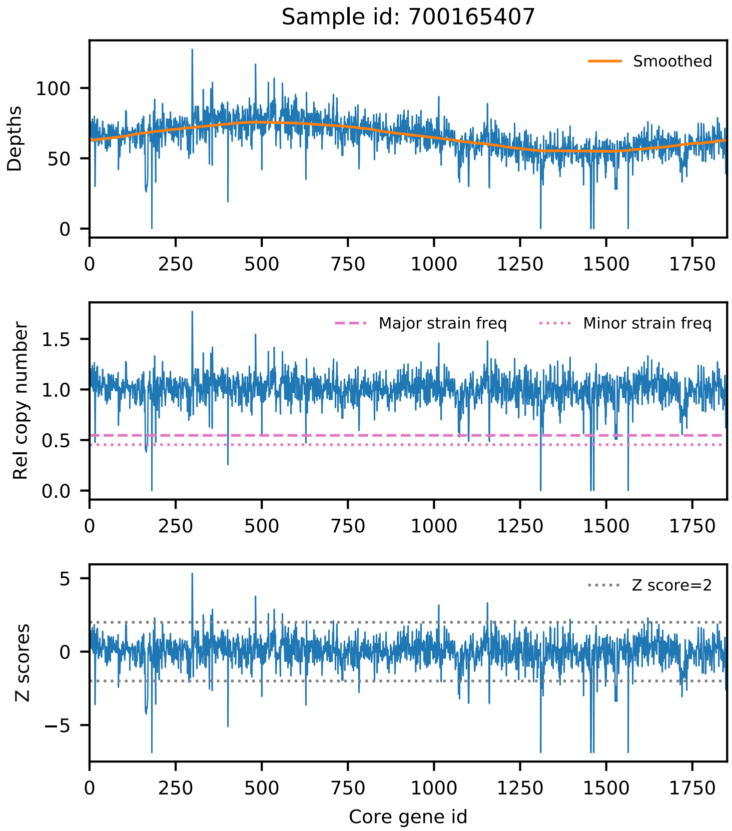

Fig Y. Filtering within-host gene deletion events using read depths. An example of our filtering algorithm in S1 Text 4.1 applied to a single sample that is co-colonized by two *B. vulgatus* strains. Top: Median read depth for all core genes, sorted according to their location on the reference genome. Orange line shows a moving average of 500 genes. Mid: Relative copy number of all core genes, computed as the median read depth of each gene divided by the moving average in the top panel. Dashed and dotted lines represent the inferred major and minor strain frequencies, respectively. If a gene is only present in the major or minor strain, then its relative copy number will be approximately equal to that strain's frequency. If a gene is missing from both strains, the relative copy number will be zero. Bottom: Z-score of all genes computed using the relative copy number in the middle panel. Genes with $|z| > 2$ are flagged as potential gene deletion events and are filtered from all downstream analyses. The data underlying this Figure can be found in https://doi.org/10.5281/zenodo.10304481

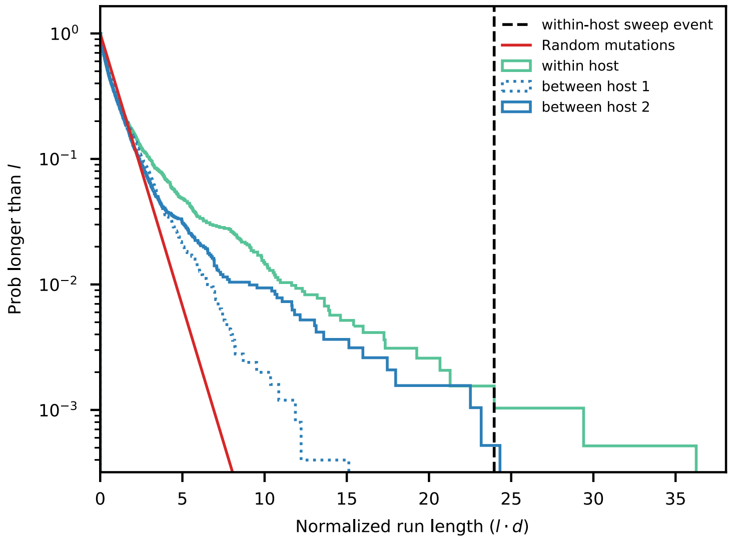

Fig Z. Distribution of shared fragment lengths for the genomes in Fig 5D. All homozygous runs of zero SNVs are identified for the pairs shown in Fig 5D, and the cumulative distribution of run lengths is shown here. The run length is normalized by the pairwise divergence d. Only the second time point of the within-host example is plotted. For comparison, the black dashed line indicates the length of the within-host sweep event in Fig 5C, while the red line shows the expected distribution if the SNVs were randomly distributed along the genome (S1 Text 4.2). These data show that random pairs of strains can sometimes share fragments that are even longer than the within-host sweep event (e.g. between-host pair 2). The data underlying this Figure can be found in https://doi.org/10.5281/zenodo.10304481

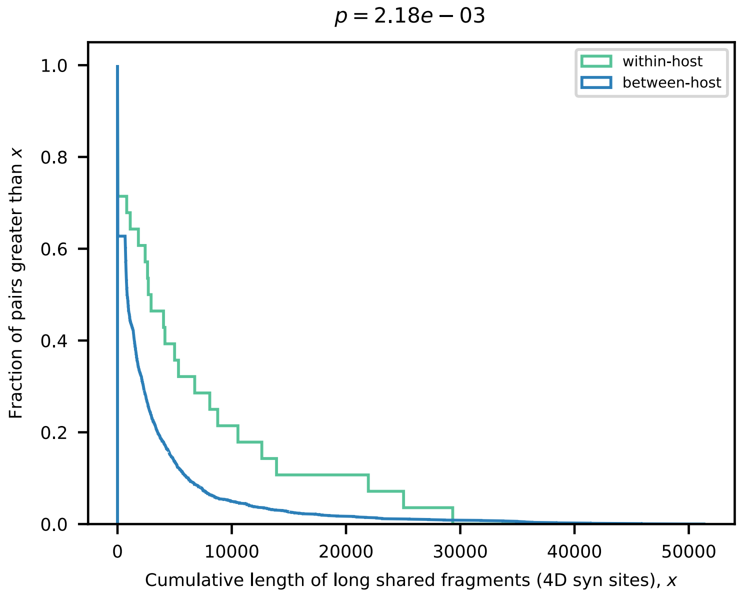

Fig AA. Distribution of the total length of long homozygous runs for *Eubacterium rectale*. Analogous version of Fig 5E using an alternative test statistic, based on the total length of runs longer than 600 synonymous sites (equivalent to $\ell^* \cdot \overline{d} \approx 30$). Similar to the "max run" statistic in Fig 5E, this new metric also demonstrates that *E. rectale* is statistically enriched for within-host sharing. The data underlying this Figure can be found in https://doi.org/10.5281/zenodo.10304481

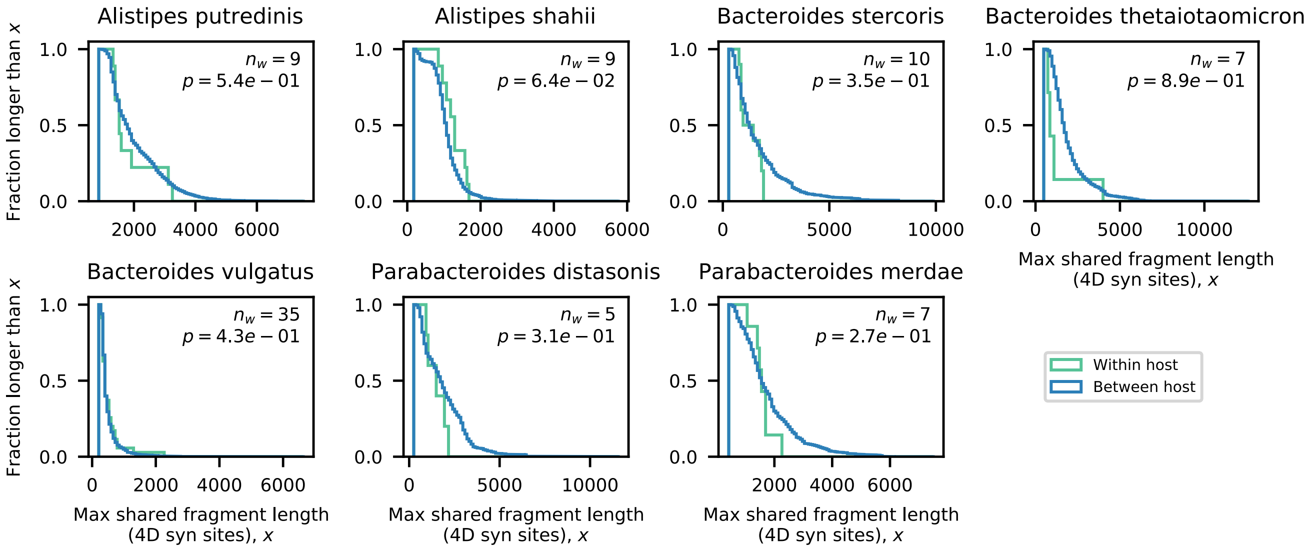

Fig BB. Distribution of the longest homozygous runs within hosts. Analogous versions of Fig 5E for the remaining species that had at least 5 hosts that passed our filtering criteria (S1 Text 4.1). None of the species here, including highly recombinogenic ones such as *A. putredinis*, are statistically enriched for within-host sharing. The data underlying this Figure can be found in https://doi.org/10.5281/zenodo.10304481

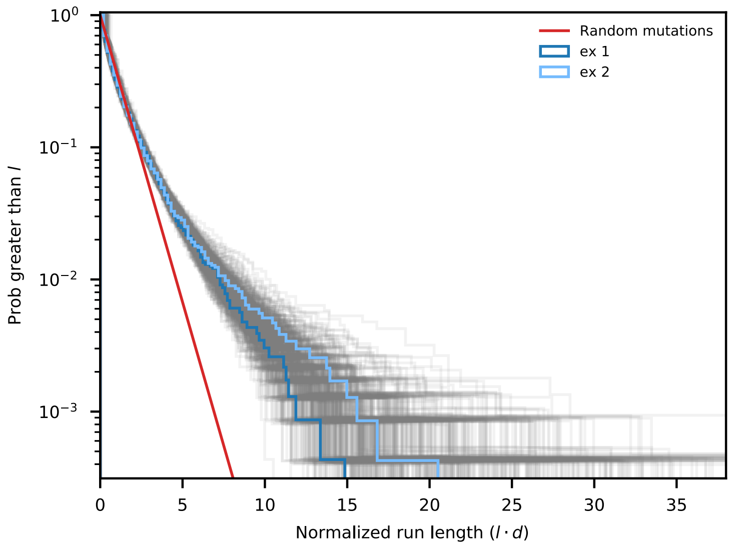

Fig CC. Distribution of shared fragment lengths in neutral simulations. Analogous version of Fig Z constructed from 500 simulated genomes from FastSimBac. Simulation parameters are identical to the lower right panel of Fig B. Only pairs with $< 10\%$ of identical blocks are shown here. The distribution of two random pairs are highlighted in blue as examples, and all others are plotted in grey. As expected from our approximate formula for the probability of observing long sharing fragments (Eq. S32), it is possible to find pairs in this neutral simulation that share a fragment as long as the within-host sweep event in Fig Z. The data underlying this Figure can be found in https://doi.org/10.5281/zenodo.10304481

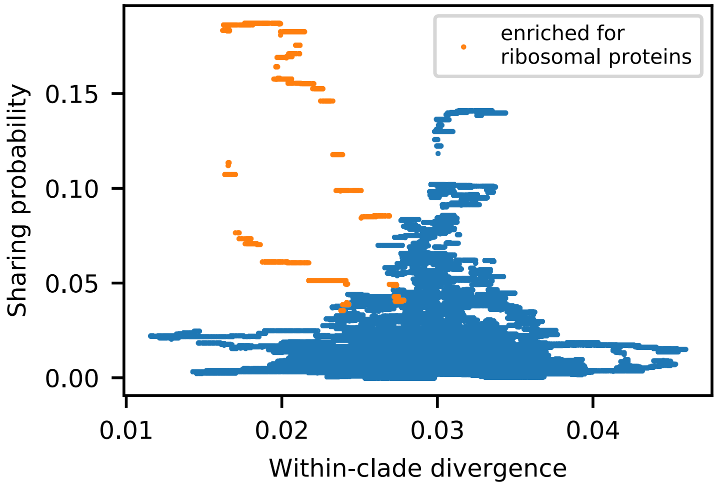

Fig DD. Local variation in the sharing of long fragments does not correlate with local divergence. Joint distribution the local sharing probability and divergence from Fig 6B. These two variables are largely uncorrelated except for the region highlighted in orange, which corresponds to the major sharing peak around position 60,000. Interestingly, this region has the highest concentration of ribosomal proteins along the genome, which could potentially be the driver of this sharing hotspot. The data underlying this Figure can be found in https://doi.org/10.5281/zenodo.10304481

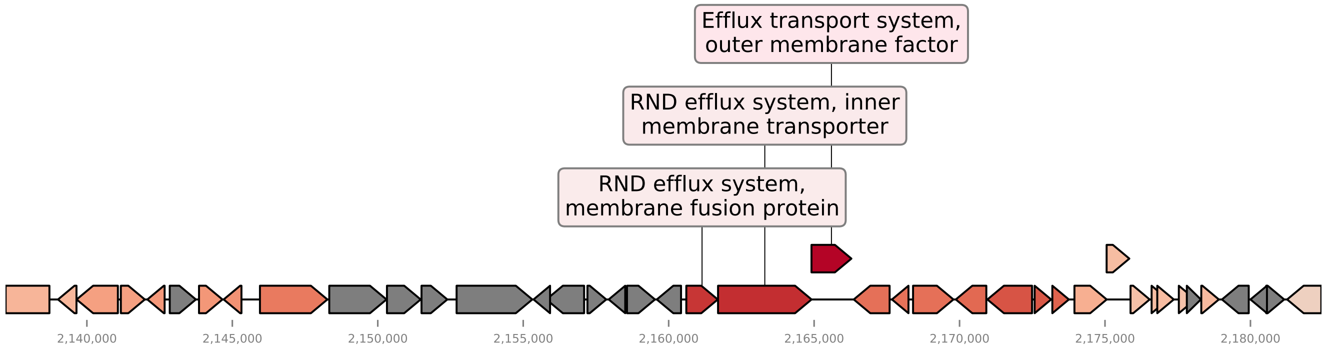

Fig EE. Global sharing probability of genes involved in the within-host sweep event in Fig 5C. Shown here is a portion of the *B. vulgatus* reference genome, roughly corresponding to the genes in the putative within-host sweep event in Fig 5C (Table S5). Three genes encoding a RND efflux system are annotated with text boxes. Core genes are colored according to their global sharing probabilities computed in Fig 6B, with warmer color representing higher frequency. Non-core genes are shown in grey. This detailed view reveals that the RND efflux system has the highest sharing probability in this region. This suggests that selection for different genetic variants of this efflux pump could be driving both the within-host sweep event and the global sharing hotspot. The data underlying this Figure can be found in https://doi.org/10.5281/zenodo.10304481

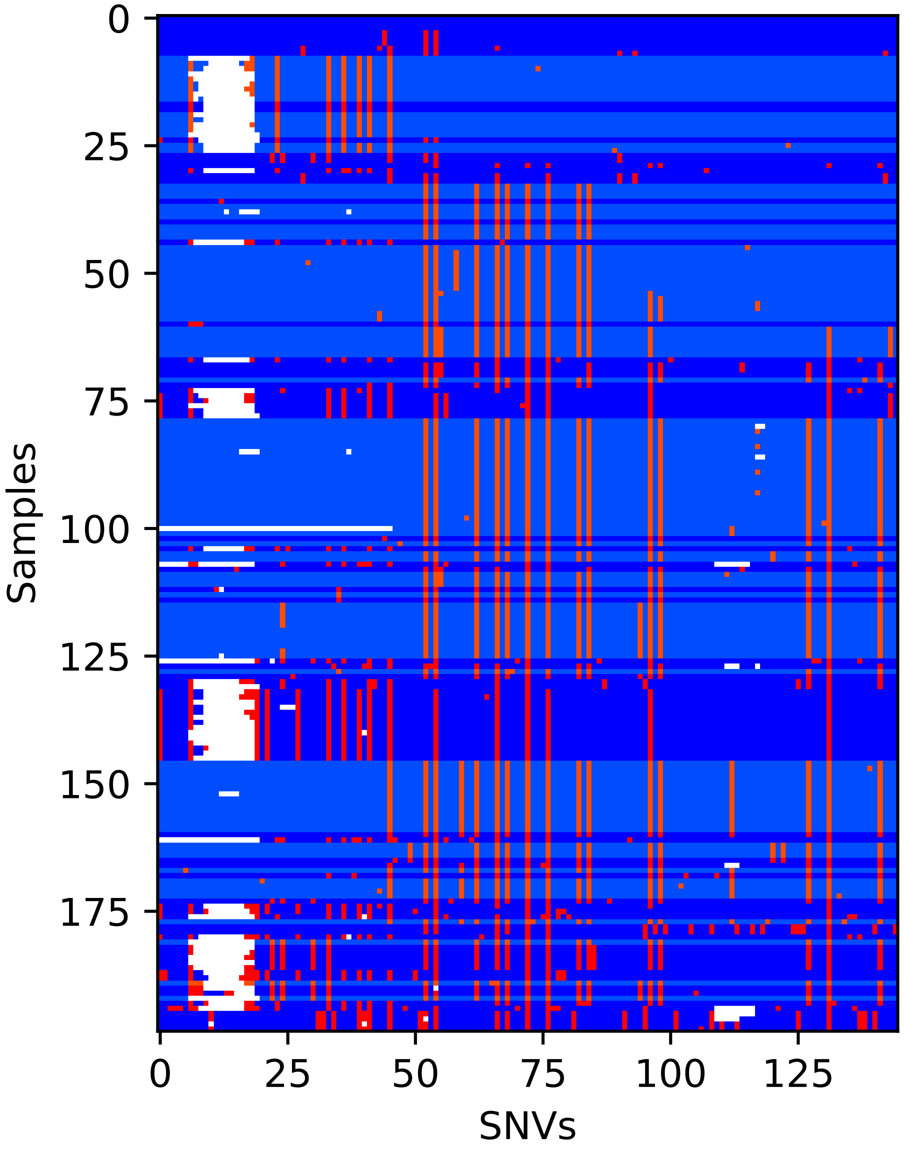

Fig FF. Multiple haplotypes contribute to a global sharing hotspot. Visualization of the observed haplotyes within the global sharing hotspot in Fig EE. Columns represent all variable sites in three genes encoding an efflux pump in Fig EE. Rows represent the "quasi-phased" strains sampled from unrelated hosts that share the same major clade as the within-host strains in Fig 5C. Sites that match the within-host alleles are shown in blue, while those with different alleles are shown in red; white tiles denote missing data. Strain rows are sorted according to their sequence divergence relative to the within-host sequence in Fig 5C. Strains rows are highlighted in lighter colors if they are in the pairs that contribute to the global sharing probability at these genes (Fig 6). These data show that while a few strains share the same haplotype as the within-host strains (completely blue rows on the top end), the majority of strains contributing to the sharing hotspot do not share the same sequence. In addition, the highlighted regions show that multiple distinct haplotypes (light colored blocks with the same red locations) contribute to this sharing hotspot. This suggests that the sharing hotspots may be driven by soft selective sweeps of transferred genome segments. The data underlying this Figure can be found in https://doi.org/10.5281/zenodo.10304481

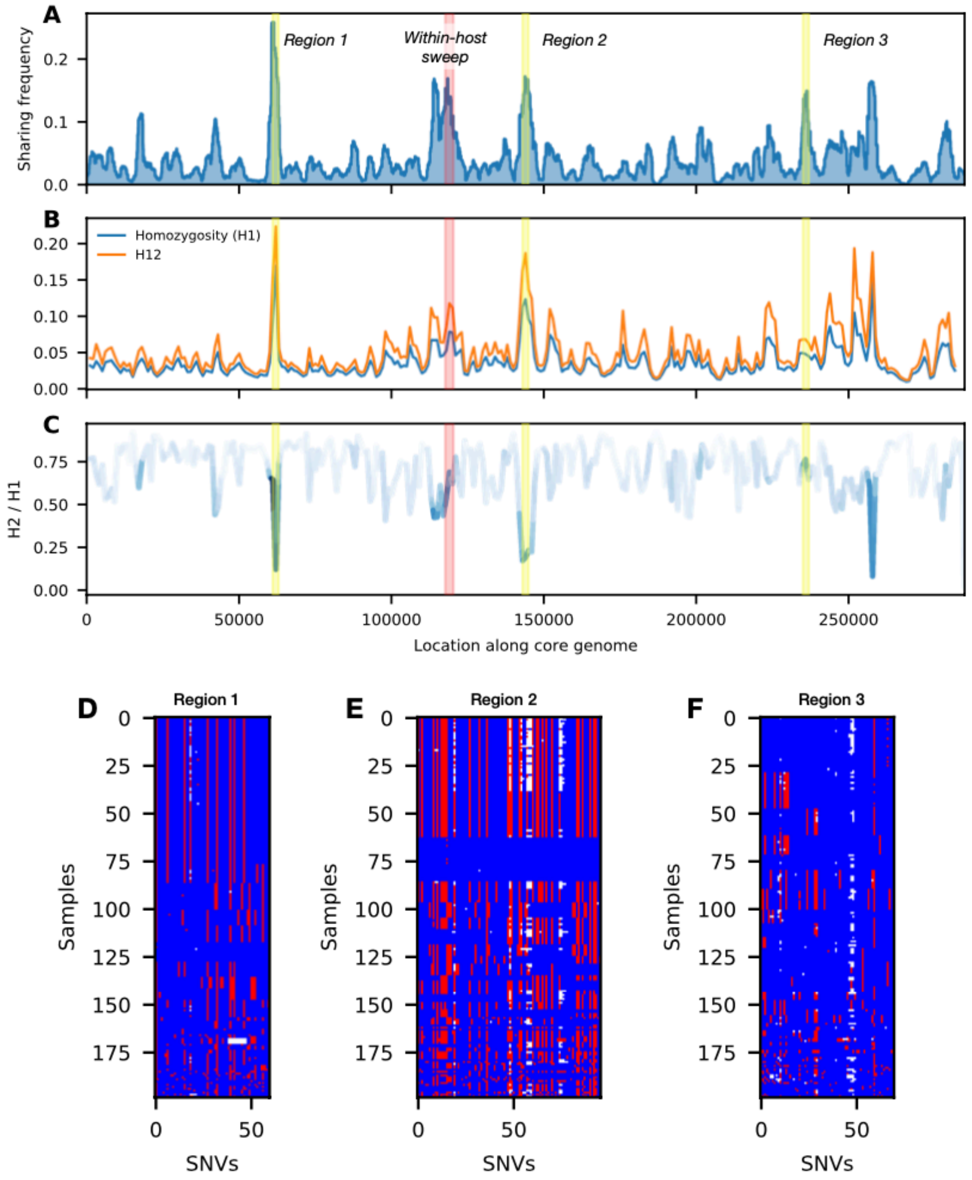

Fig GG. Comparison between sharing probability and haplotype homozygosity. The within-clade sharing landscape of *Bacteroides vulgatus* is compared with various selection tests based on haplotype homozygosity. (A): Same data as Fig 6B. In addition to the within-host sweep event (Fig 5C, three more 2kb regions corresponding to sharing hotspots are highlighted in yellow. (B) Haplotype homozygosity scan along the core genome. H1 is the conventional haplotype homozygosity, while H12 is the analogous statistic with the largest two haplotypes combined [51]; elevated regions of H1 or H12 indicate candidate regions undergoing selective sweeps, with H12 having better power for detecting soft sweeps. Scans were performed in 2kb windows, and haplotypes were defined by clustering samples with < 2 SNV differences in the window. (C) Ratio between H2 (the haplotype homozygosity after excluding the largest haplotype [51]) and H1. This ratio quantifies the hardness of a sweep, with lower values indicating the presence of one dominant haplotype. (D-F) Visualization of haplotypes in the highlighted regions detected by sharing probability, analogous to Fig FF. Regions 1 and 2 are examples of hard sweeps dominated by one haplotype; in these cases, the sharing probability metric gives comparable values to H1 and H12. Region 3 is a candidate of a very soft sweep, which is hard to detect for both H1 and H12. These results suggest that the sharing probability metric is capable of recovering signals of existing selection tests based on haplotype homozygosity, and has enhanced power for detecting soft sweeps. The data underlying this Figure can be found in https://doi.org/10.5281/zenodo.10304481

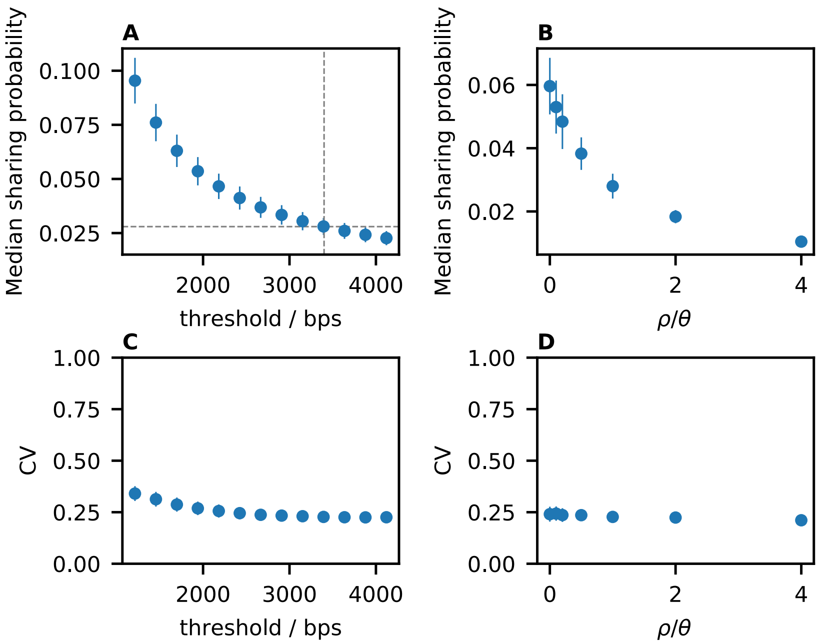

Fig HH. Sharing landscape statistics in neutral simulations. Data shown here are generated using FastSimBac (S1 Text 5.3). (A) Median sharing probability across all genome positions for a range of threshold lengths ($\rho/\theta = 1$, same data as Fig 6B). Crosshair shows the threshold length that matches the median within-clade sharing probability of *B. vulgatus*. (B) Median sharing probability for a range of recombination rates. (C,D) Coefficient of variation (CV) of the sharing landscape for each of the datapoints in panels A and B, respectively. In all panels, error bars represent one standard deviation among 100 replicates. These results show that neutral models predict small fluctuations in the sharing probability across the genome, regardless of the threshold length or ρ/θ. The data underlying this Figure can be found in https://doi.org/10.5281/zenodo.10304481

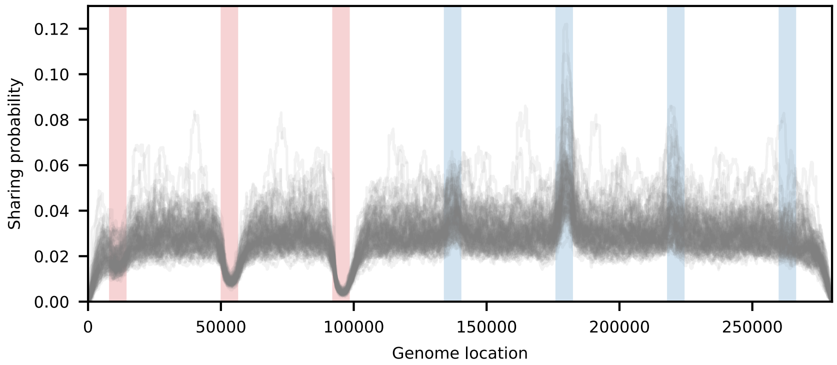

Fig II. Effects of recombination rate variation on the sharing of long segments. FastSimBac simulations of a neutral population with various recombination hotspots/coldspots along the genome, highlighted in red/blue. Data shown here are 100 simulations using the same base parameters as Fig 6B, with local modifications of the recombination rate in the highlighted regions; from left to right, the recombination rates relative the default value are $\{2,\ 5,\ 10,\ 0.5,\ 0.1,\ 0.01,\ 0\}$. These simulations show that the sharing probability decreases dramatically in recombination hotspots across all replicates. Conversely, lowering the recombination rate increases the sharing probability slightly, but not enough to reproduce sharing hotspots observed in real data. Curiously, this enhancement of sharing due to reduced recombination is non-monotonic. The data underlying this Figure can be found in https://doi.org/10.5281/zenodo.10304481

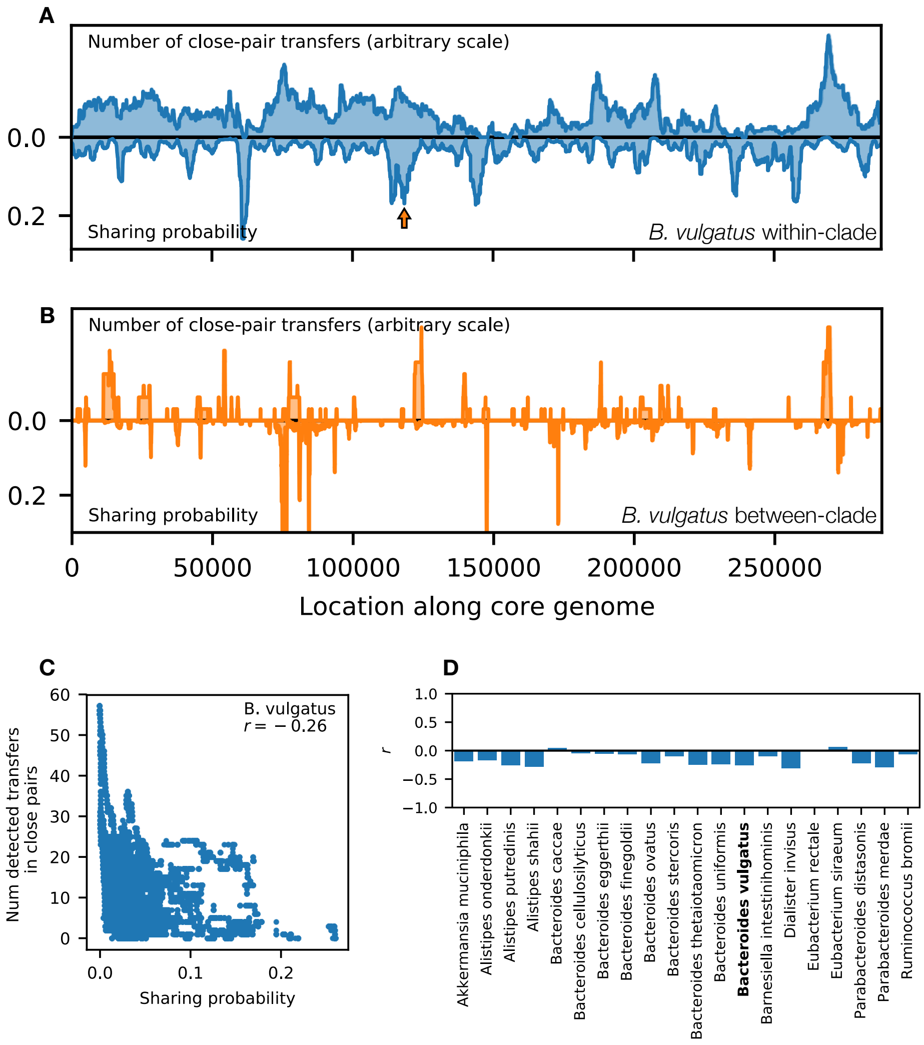

Fig JJ. Comparing the landscapes of detected transfers and recent haplotype sharing. (A) Example landscapes computed for strains within the major clade of *Bacteroides vulgatus*. Top: the number of detected transfers between closely related pairs at each location. A deduplication step was performed to avoid overcounting transfer events (S1 Text 3.9). Bottom: same data as Fig 6B (within-clade). Only four-fold degenerate (4D) sites along the core genome are shown. Visually, there is only limited correlation between the two landscapes. Although the highest peaks in either landscape corresponds to the lowest trough in the other, we also observe regions with high values of both haplotype sharing and detected transfers (e.g. the sweep region in Fig 5, marked by orange arrow). (B) Analogous version of panel A for recombination between the major clades of *B. vulgatus*. (C) Scatter plot showing weak correlation between the values of two landscapes in panel A at each site along the core genome. Inset shows the Pearson correlation coefficient, r. (D) Correlation coefficient of landscape values for all species with sufficient data, analogous to panel B. The limited correlation in *B. vulgatus* is shared across all species. This is consistent with the simulation results in Fig II, which suggest that the haplotype sharing hotspots are driven by selection on transferred fragments, rather than local variation in the recombination rate. The data underlying this Figure can be found in https://doi.org/10.5281/zenodo.10304481

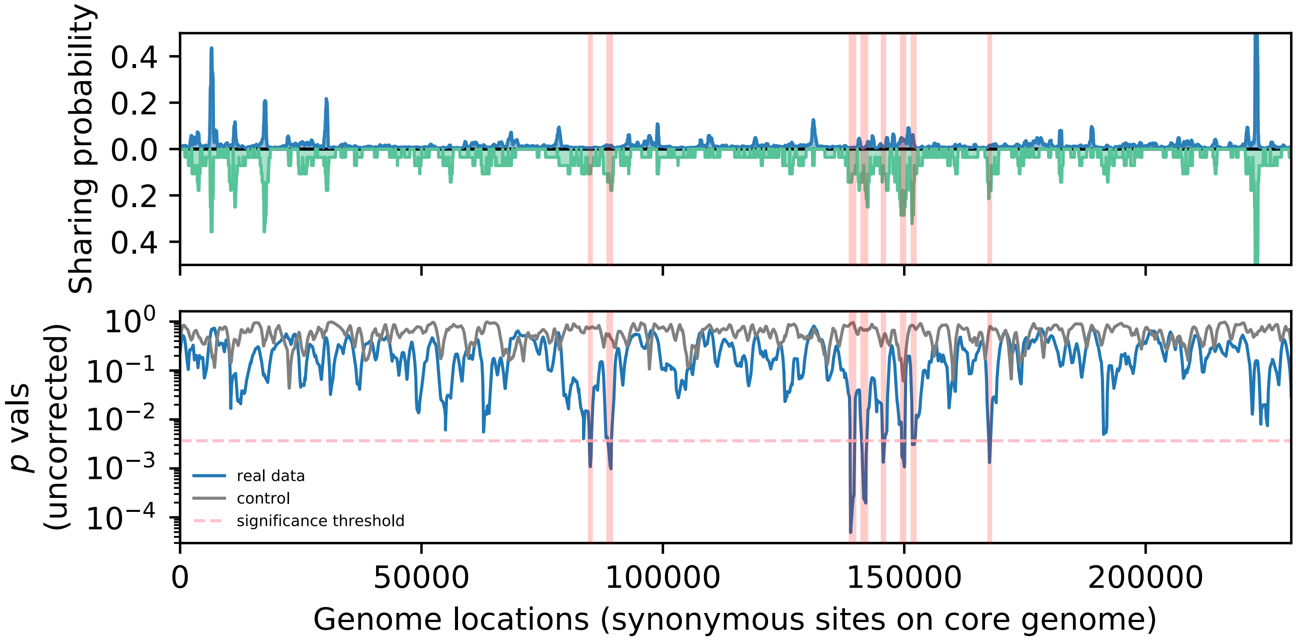

Fig KK. Within-host enrichment in the sharing landscape of *Eubacterium rectale*. Top panel: Analogous version of Fig 6C, that highlights the regions that were identified to be enriched in within-host sharing (S1 Text 5.4). Bottom panel: First level P values from double-bootstrap permutation test (S1 Text 5.4), averaged in sliding windows of 1000 sites. Grey curve shows the result of a negative control, obtained by applying the test to an independent permutation. Pink dashed line represents the genome-wide significance level ($P < 0.05$) for first level P values. Regions with statistically significant differences in within-host sharing are highlighted in both panels. The data underlying this Figure can be found in https://doi.org/10.5281/zenodo.10304481

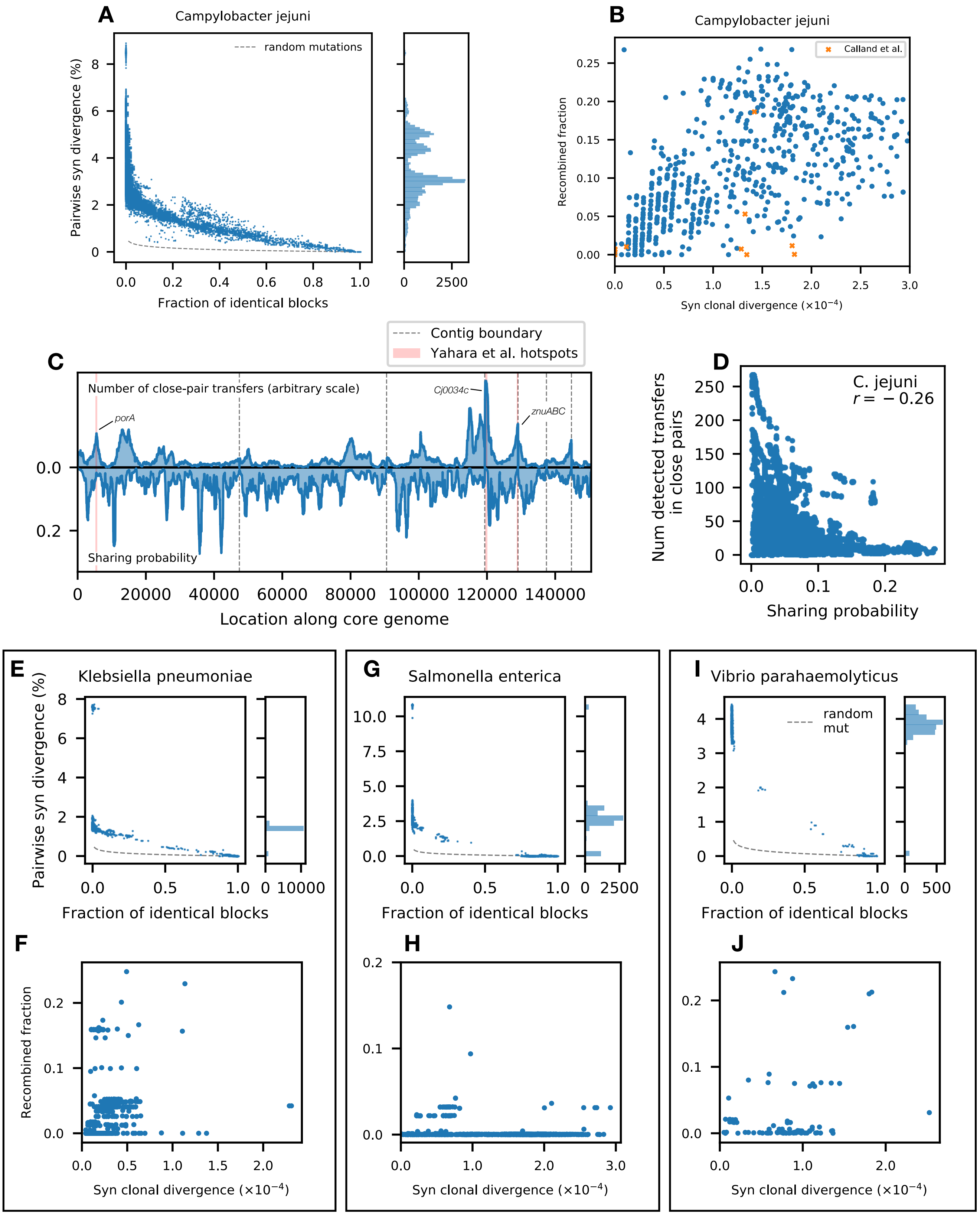

A
Campylobacter jejuni
random mutations
Pairwise syn divergence (%)
Fraction of identical blocks
B
Campylobacter jejuni
Calland et al.
Recombined fraction
Syn clonal divergence (×10⁻⁴)
C
Contig boundary
Yahara et al. hotspots
Number of close-pair transfers (arbitrary scale)
porA
Cj0034c
znuABC
Sharing probability
Location along core genome
D
C. jejuni
r = −0.26
Num detected transfers in close pairs
Sharing probability
E
Klebsiella pneumoniae
Pairwise syn divergence (%)
Fraction of identical blocks
G
Salmonella enterica
Fraction of identical blocks
I
Vibrio parahaemolyticus
random mut
Fraction of identical blocks
F
Recombined fraction
Syn clonal divergence (×10⁻⁴)
H
Syn clonal divergence (×10⁻⁴)
J
Syn clonal divergence (×10⁻⁴)

Fig LL. CP-HMM analysis of isolate genomes from bacterial pathogens. CP-HMM was applied to a total of 685 previously sequenced isolate genomes from 4 commonly studied bacterial pathogens (S1 Text 3.10). (A) Analogous version of Fig 1C for *Campylobacter jejuni*, showing a large number of closely related pairs. (B) Analogous version of Fig 3A-C. Orange crosses show the analogous data points reported in Ref. 41, where recombined regions were inferred for a smaller sample of 12 strains using Gubbins [22]. These data demonstrate that the apparent recombination rates reported in Ref. 41 are consistent with the results inferred using the CP-HMM algorithm. (C-D) Analogous versions of Fig JJA-B. The peaks in the landscape of detected transfers reproduce the "recombination hot regions" previously reported in Ref. 39. However, the sharing landscape (C, bottom) reveals a different set of locations that are enriched for recent haplotype sharing. This suggests that these two landscapes reflect different aspects of the interplay between recombination and natural selection. (E-J) Analogous versions of A-B for three other pathogen species. It is worth noting that *Salmonella enterica* contains both a large number of pairs with minimal recombination and many pairs with significant recombination. This is consistent with previous studies showing that r/m in *S. enterica* can differ by 10-fold between sublineages [46]. The data underlying this Figure can be found in https://doi.org/10.5281/zenodo.10304481

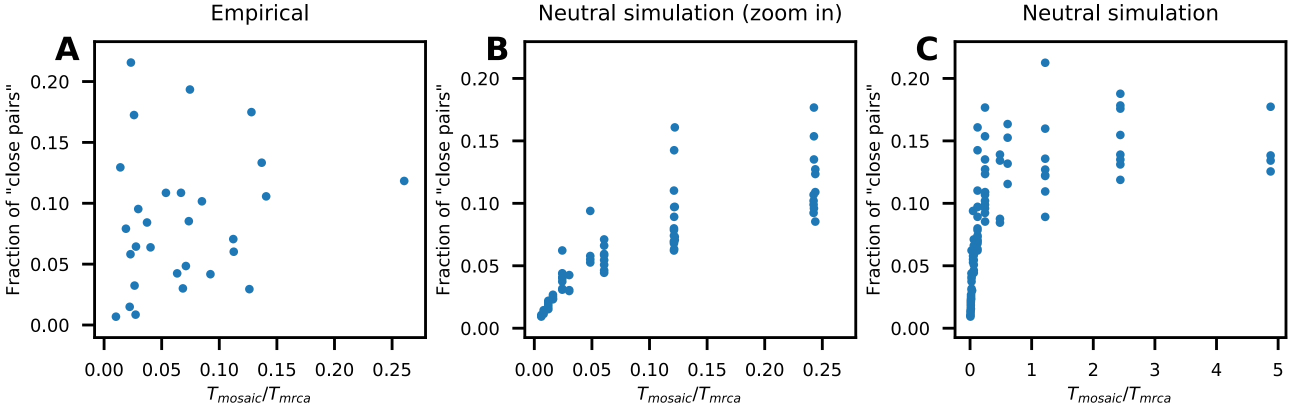

Fig MM. Correlation between $T_{\mathrm{mosaic}}/T_{\mathrm{mrca}}$ and the number of closely related pairs in different species. (A) Horizontal axis shows the average $T_{\mathrm{mosaic}}/T_{\mathrm{mrca}}$ estimates for each species in Fig R, while the vertical axis shows the fraction of genome pairs that have $> 20\%$ identical blocks (S1 Text 2). (B) Correlation between the true value of $T_{\mathrm{mosaic}}/T_{\mathrm{mrca}}$ and the number of closely related pairs in neutral simulations. We simulated 100 populations using FastSimBac (S1 Text 5.3) with $\rho/\theta = \{0.1, 0.5, 1, 1.5, 2\}$, $\theta \approx 0.008$, $\lambda = \{500, 1000, 2000, 5000, 10000\}$, sample size 200, covering a wide range of $T_{\mathrm{mosaic}}/T_{\mathrm{mrca}}$ values. (C) Analogous version of panel B showing a wider range of $T_{\mathrm{mosaic}}/T_{\mathrm{mrca}}$ values. Unlike the neutral simulations, the observed data show little correlation between the fraction of close pairs and $T_{\mathrm{mosaic}}/T_{\mathrm{mrca}}$. Moreover, some species can have high fractions of close pairs at very low $T_{\mathrm{mosaic}}/T_{\mathrm{mrca}}$ values, in sharp contrast with the predictions of the neutral simulations. The data underlying this Figure can be found in https://doi.org/10.5281/zenodo.10304481

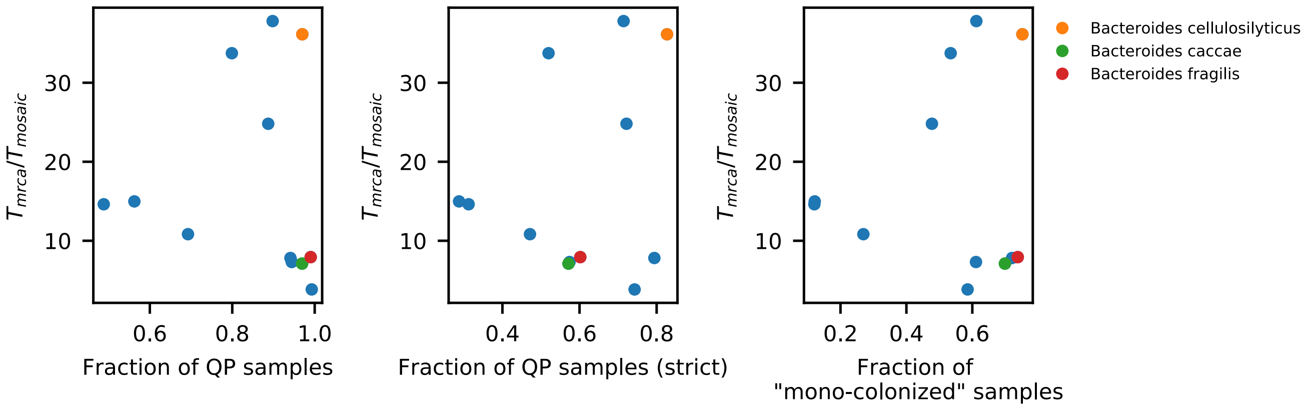

Fig NN. Colonization strategies are not correlated with recombination rates inferred from close pairs. Data shown here are from 12 species in the *Bacteroides* genus, with each point representing a species. In all three panels, the vertical axis shows the estimated $T_{\mathrm{mrca}}/T_{\mathrm{mosaic}}$ (Fig R). Left panel: Horizontal axis shows the fraction of "quasi-phaseable" (QP) samples for that species, which serves a proxy for the degree of single- vs multi-colonization (Fig A). For example, *B. fragilis*, a species known to have mostly clonal within-host colonization structure [53], has a QP fraction of $> 99\%$. Middle panel: horizontal axis shows the fraction of QP samples under a stricter definition of quasi-phaseability – instead of defining alleles with frequencies between 0.2 and 0.8 as polymorphic [1], this stricter version expands the frequency range to between 0.05 and 0.95. Right panel: horizontal axis shows an even stricter definition of single-colonization – any site with more alternative alleles that what is expected under a Poisson error model will be considered a polymorphic site, and a sample is "single-colonized" if the polymorphism rate is lower than 0.1%. Interestingly, all three panels show little correlation between the apparent degree of multi-colonization and inferred recombination rates. In particular, species that exhibit the lowest levels of multi-colonization can have drastically different rates of recombination (e.g. *B. cellulosilyticus* vs *B. caccae*). This lack of correlation suggests that physical proximity may not be the main driver of the variation in the inferred recombination rates between species. The data underlying this Figure can be found in https://doi.org/10.5281/zenodo.10304481

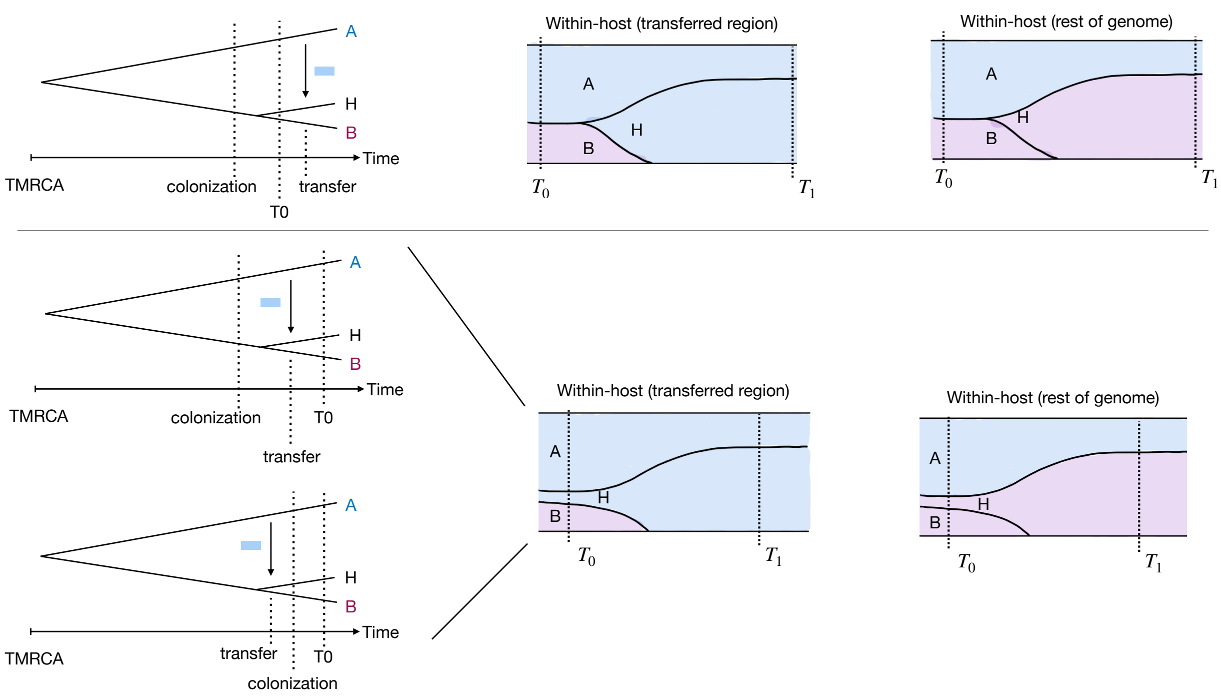

Fig OO. Different genealogical scenarios that can explain the within-host sweep event in Fig 5C. Top left: an example where the transfer event occurs within the sampling window (i.e. between T_0 and T_1), and subsequently sweeps to fixation. The fixation of the hybrid strain leads to different ancestry patterns in the transferred region (middle) vs the rest of the genome (right), which manifests as a depletion of within-host SNVs in the transferred region (Fig 5C). Bottom: alternative scenarios where the transfer occurs before T_0, or even prior to the colonization of the focal host (e.g. if the strains both transmitted from a close relative). All three scenarios lead to a gene-specific sweep between T_0 and T_1, as long as the hybrid strain starts at a sufficiently low frequency at T_0.
